# Comparative Analysis of NADPH-Cytochrome P450 Reductases From Legumes for Heterologous Production of Triterpenoids in Transgenic *Saccharomyces cerevisiae*

**DOI:** 10.3389/fpls.2021.762546

**Published:** 2021-12-16

**Authors:** Pramesti Istiandari, Shuhei Yasumoto, Pisanee Srisawat, Keita Tamura, Ayaka Chikugo, Hideyuki Suzuki, Hikaru Seki, Ery Odette Fukushima, Toshiya Muranaka

**Affiliations:** ^1^Department of Biotechnology, Graduate School of Engineering, Osaka University, Osaka, Japan; ^2^Industrial Biotechnology Initiative Division, Institute for Open and Transdisciplinary Research Initiatives, Osaka University, Osaka, Japan; ^3^RIKEN Center for Sustainable Resource Science, Wako, Japan; ^4^Department of Research and Development, Kazusa DNA Research Institute, Kisarazu, Japan; ^5^Plant Translational Research Group, Universidad Regional Amazónica IKIAM, Tena, Ecuador

**Keywords:** legumes, cytochrome P450 monooxygenases (CYP), NADPH-cytochrome P450 reductases (CPR), triterpenoids, heterologous production, *Saccharomyces cerevisiae*

## Abstract

Triterpenoids are plant specialized metabolites with various pharmacological activities. They are widely distributed in higher plants, such as legumes. Because of their low accumulation in plants, there is a need for improving triterpenoid production. Cytochrome P450 monooxygenases (CYPs) play critical roles in the structural diversification of triterpenoids. To perform site-specific oxidations, CYPs require the electrons that are transferred by NADPH-cytochrome P450 reductase (CPR). Plants possess two main CPR classes, class I and class II. CPR classes I and II have been reported to be responsible for primary and specialized (secondary) metabolism, respectively. In this study, we first analyzed the CPR expression level of three legumes species, *Medicago truncatula*, *Lotus japonicus*, and *Glycyrrhiza uralensis*, showing that the expression level of CPR class I was lower and more stable, while that of CPR class II was higher in almost all the samples. We then co-expressed different combinations of CYP716As and CYP72As with different CPR classes from these three legumes in transgenic yeast. We found that CYP716As worked better with CPR-I from the same species, while CYP72As worked better with any CPR-IIs. Using engineered yeast strains, CYP88D6 paired with class II GuCPR produced the highest level of 11-oxo-β-amyrin, the important precursor of high-value metabolites glycyrrhizin. This study provides insight into co-expressing genes from legumes for heterologous production of triterpenoids in yeast.

## Introduction

Triterpenoids are one of the most diverse groups of plant-specialized metabolites, with more than 20,000 compounds known to exist in nature (Oldfield and Lin, 2012). One of the triterpenoid groups, soyasaponin, is a structurally complex oleanane triterpenoid commonly found in legume plants. Soyasaponins have diverse biological functions such as anti-inflammatory, anti-carcinogenic, and cardiovascular-protective activities (Guang et al., 2014). Some triterpenoid saponins are unique in certain species within the legume family, such as glycyrrhizin, which is found only in the *Glycyrrhiza* species. Glycyrrhizin shows high pharmacological activities and is used as a natural sweetener because its sweetness is 150 times higher than that of sucrose (Kitagawa, 2002).

**FIGURE 1:**
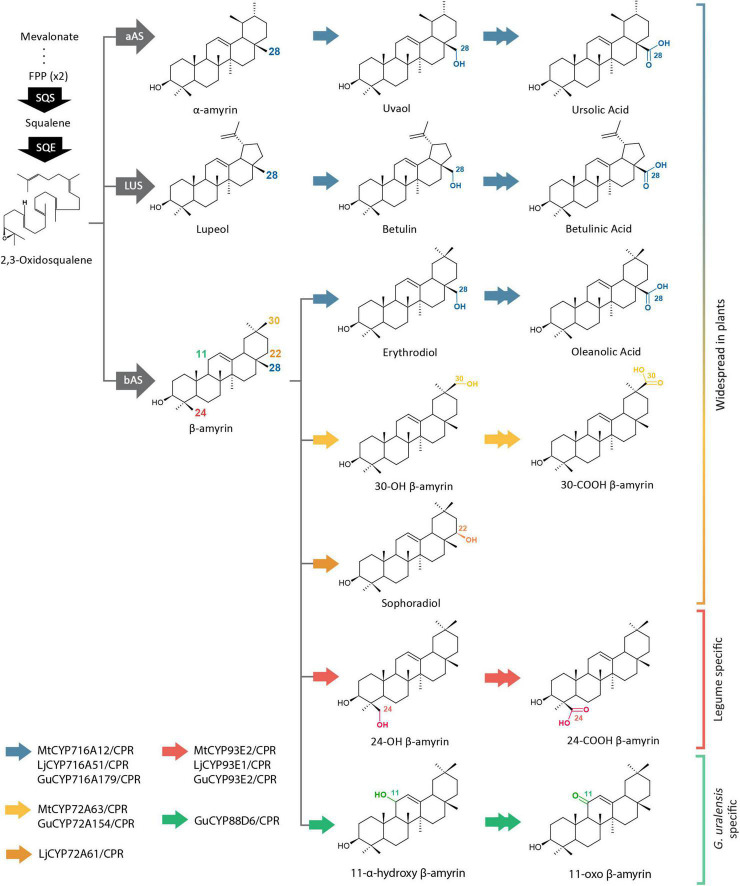
Triterpenoid biosynthetic pathways of several CYP families from *Medicago truncatula* (Mt), *Lotus japonicus* (Lj), and *Glycyrrhiza uralensis* (Gu). Single and double arrows indicate one and two oxidation steps, respectively. CYP, cytochrome P450; FPP, farnesyl pyrophosphate; SQS, squalene synthase; SQE, squalene epoxidase; bAS, β-amyrin synthase; aAS, α-amyrin synthase; LUS, lupeol synthase.

Cytochrome P450 monooxygenases (CYPs) are the most important enzymes responsible for the structural diversity of triterpenoids (Xu et al., 2004). CYPs are classified based on their sequence similarities (Moktali et al., 2012). The CYP716A and CYP72A subfamily are widespread in plants and show diverse functions in triterpenoid biosynthesis, while other CYP subfamilies such as CYP93E and CYP88D are known to be unique to legumes (Seki et al., 2015; Figure 1). To perform catalysis, CYPs require the electrons that are transferred by nicotinamide adenine dinucleotide phosphate (NADPH)-cytochrome P450 reductases (CPRs). Electrons from NADPH flow through flavin adenine dinucleotide (FAD) to the flavin mononucleotide (FMN) domains of CPRs, which are finally transferred to the heme center of CYPs (Paine et al., 2005). CPR and CYP are both membrane-bound proteins that have been reported to be present in microsomes in a ratio of 1:15 (Shephard et al., 1983). This high ratio of CYP to CPR implies that CYPs compete for CPRs and that they must be particularly organized in order to ensure electron supply from CPRs to huge numbers of CYPs at once (Shephard et al., 1983). A previous study on human CPR (hCPR) showed that specific mutations in the FMN domain improved interactions with a specific CYP (Esteves et al., 2020). Several point mutations were found to be co-localized with mutations found in naturally occurring hCPR variants that cause CYP isoform-dependency (Pandey and Flück, 2013; Burkhard et al., 2017) and negatively charged residues that have been previously suggested to be important in CPR:CYP interactions (Hamdane et al., 2009; Hong et al., 2010; Jang et al., 2010).

Unlike mammals and fungi with one CPR gene, plants have multiple CPR genes, depending on the species (Mizutani and Ohta, 1998; Jensen and Møller, 2010). Plant CPRs are branched into two classes, CPR class I and class II (Qu et al., 2015), which in this paper will be referred to as CPR-I and CPR-II, respectively. CPR-I generally has a shorter N-terminal membrane sequence than CPR-II (Parage et al., 2016). CPR-I is reported to be constitutively expressed and plays a role in primary or basal constitutive metabolism, while CPR-II is inducible by environmental stimuli and involves more defense mechanisms through plant secondary metabolism (Ro et al., 2002; Yang et al., 2010; Rana et al., 2013; Qu et al., 2015; Parage et al., 2016). As reported in previous study that knocking down the *CPR2* gene in *Catharanthus roseus* resulted in a significant decrease in the monoterpene indole alkaloid content. In contrast, knocking down the *CPR1* gene did not result in any modification of the monoterpene indole alkaloid content (Parage et al., 2016).

Many CYPs from legumes have already been used for heterologous production of triterpenoids. Recently, glycyrrhizin has been successfully produced *de novo* in yeast by the combinatorial expression of different enzymes (Chung et al., 2020). Pairing different CPRs with different CYPs has also been reported to result in different productivity of plant specialized metabolites in heterologous yeast (Zhu et al., 2018). In previous studies, CPRs were randomly selected without consideration of the classes (Zhu et al., 2018; Jin et al., 2019). Therefore, the CPR screening was still a subject of trial and error, which might lead to an ineffective strategy for improving heterologous production.

In this study, we performed phylogenetic and gene co-expression analysis of plant CPR classes from two model legumes, *Medicago truncatula* and *Lotus japonicus*, and a medicinal legume, *Glycyrrhiza uralensis*, to clarify the differences in sequence and gene expression patterns. Through co-expression of different CPR classes and CYP families in heterologous yeast, we observed different preferences of plant CPR classes toward specific CYPs. This is the first comparative analysis of different CPR classes of legumes, which gave insight into the differential functions of CPR classes toward the heterologous production of triterpenoids.

## Materials and Methods

### Phylogenetic and Structural Analysis of Plant CPR Classes

Plant CPR sequences from 20 plant species were obtained from the web-based resource for Arabidopsis P450, cytochrome b5, NADPH-cytochrome P450 reductases, and family 1 glycosyltransferases^1^, NCBI^2^, and each plant genome database. Accession numbers of CPR genes and genome databases used in this study are listed in Supplementary Table 1. Multiple sequences were aligned using ClustalW and were used for tree construction using MEGA7 with the neighbor-joining method. The position of protein helices was predicted using an *in silico* transmembrane helix prediction tool^3^ (Supplementary Figure 1). The conserved membrane anchor, FMN-, FAD-, CYP-, and NADPH-binding domains were predicted as described by Qu et al. (2015). Motif analysis of the FMN domain of legume CPR classes I and II was performed using the MEME Suite online software^4^ using a minimum of 37 sequences for each CPR class from 24 different legume species (Supplementary Figure 2). The structure model of MtCPRs was constructed using 5gxu.1.A (crystal structure of Arabidopsis ATR2) as a template with >71% identity in the SWISS-MODEL (Arnold et al., 2006). PyMol (DeLano, 2020) was used to visualize the amino acids at the active site of the enzyme.

### Chemicals

β-amyrin, erythrodiol, oleanolic acid, α-amyrin, uvaol, ursolic acid, and lupeol were purchased from Extrasynthese (Genay, France). Betulin, methyl jasmonate, and salicylic acid were purchased from Sigma-Aldrich (St. Louis, MO, United States). Betulinic acid and gibberellin were purchased from Tokyo Chemical Industry (Tokyo, Japan). Sophoradiol, 24-hydroxy-β-amyrin, 24-carboxy-β-amyrin, 11-oxo-β-amyrin, 30-hydroxy-β-amyrin, and 11-deoxoglycyrrhetinic acid were kindly gifted by Dr. Kiyoshi Ohyama (Tokyo Institute of Technology).

### RNA Sequencing

Methods of *G. uralensis* sample preparations, RNA extraction, and library construction for *de novo* assembly have been described in Supplementary Methods.

### Co-expression Analysis

Co-expression analysis of *M. truncatula* and *L. japonicus* was performed using an online transcriptomic database from the gene expression atlas web servers https://lipm-browsers.toulouse.inra.fr/pub/expressionAtlas/app/mtgeav3 and https://lotus.au.dk/expat/ (Miyakojima MG20 v 3.0), respectively. Transcriptomic data for *M. truncatula, L. japonicus*, and *G. uralensis* were obtained from 274, 74, and 37 different samples, respectively. Pearson’s correlation coefficients (PCCs) of different CPRs and CYPs were calculated using Excel (Microsoft Corp., Redmond, WA, United States).

### N-Terminal Domain Swapping of CPR-I and II

The position of protein helix inside, transmembrane, and outside of the endoplasmic reticulum (ER) were predicted using *in silico* transmembrane helix prediction (see foot note 3) (Supplementary Figure 1). In this study, the N-terminal domain are represented by the protein located inside ER together with the transmembrane helix. To swap the N-terminal domain of MtCPR1 and MtCPR2, the first 46 amino acids of MtCPR1 are swapped with the first 60 amino acids of MtCPR2 using NEBuilder^®^ HiFi DNA Assembly cloning to produce N-terminal sequence of MtCPR1 fused with truncated-N-terminal MtCPR2 (M1N-M2C) and N-terminal sequence of MtCPR2 fused with truncated-N-terminal MtCPR1 (M2N-M1C). Two different fragments of each designed chimeric CPRs (M2N-M1C and M1N-M2C) were amplified using pENTR-MtCPR1 and pENTR-MtCPR2 as template and primers listed in Supplementary Table 2. A minimum of 20-bp overlapping nucleotides of each CPR mutant and pENTR™ as vector backbone were designed for fusion cloning.

### Cloning and Vector Construction

Cytochrome P450 reductases class I and II genes from *M. truncatula*, *L. japonicus*, and *G. uralensis* were amplified from the respective cDNA using PCR with PrimeSTAR^®^ Max DNA Polymerase (Takara Bio, Kyoto, Japan) and named *MtCPR1*, *MtCPR2*, *LjCPR1, LjCPR2*, *GuCPR1*, and *GuCPR2*. The primers used in this study are listed in Supplementary Table 2. Wild-type and chimeric *CPR* genes were cloned into the pENTR™ vector using the D-TOPO^®^ (Thermo Fisher Scientific, Waltham, MA, United States) or NEBuilder^®^ HiFi DNA Assembly (Ipswich, MA, United States) cloning method, to produce entry clones of CPR genes. Yeast expression clones of CPR, using pAG415-GAL-ccdB (plasmid number 14145, Addgene, Watertown, MA, United States) as the destination vector, were constructed using LR reaction with LR Clonase™ II Enzyme mix (Thermo Fisher Scientific), to produce pAG415-CPR. pAG415GAL-ccdB was a gift from Susan Lindquist. Yeast expression clones of *MtCYP716A12*, *MtCYP72A63*, *LjCYP716A51*, *LjCYP93E1*, *LjCYP72A61*, and *GuCYP88D6* were constructed using LR reaction with LR Clonase™ II Enzyme mix (Thermo Fisher Scientific) into pYES-DEST52 (Invitrogen, Carlsbad, CA, United States) and a Gateway™-compatible version of pESC-HIS generated previously in our laboratory (Yasumoto et al., 2016) as the destination vectors, to produce pYES-DEST52-CYP and pESC-HIS-CYP (Seki et al., 2008, 2011; Fukushima et al., 2011; Suzuki et al., 2019).

### Yeast Strain Construction

Two different yeast strains were used in this research, INV*Sc1* (MATa his3D1 leu2 trp1-289 ura3-52; Thermo Fisher Scientific) and PSIII strain. PSIII strain was generated by transforming pESC-TRP[P*_*GAL*10_/*t*HMG1-T2A-upc2-1*][P*_*GAL*1_*/Lj*bAS*]) as described in Supplementary Methods 2 into PSI strain (BY4742/P*_*erg*7_*:P*_*MET*3_*-*ERG7*/*trp1*:P*_*ACT*1_*-*Gal4dbd-ER-VP16*) constructed in a previous study (Srisawat et al., 2020). INV*Sc*1 strain carrying pYES3-ADH-aAS, pYES3-ADH-OSC1, or pYES3-ADH-LUS (Fukushima et al., 2011) and PSIII strain were then co-transformed with pAG415-CPR, pYES-DEST52-CYP, and pESC-HIS-CYP consecutively using Frozen-EX Yeast Transformation II™ (Zymo Research, Irvine, CA, United States).

### Yeast Cultivation

In case of INV*Sc*1, yeast sample with cell density at an OD_600_ value in the range of 2.2–2.5 (late logarithmic phase) were cultured in 5 mL appropriate synthetic defined medium (Clontech, Palo Alto, CA) containing 2% glucose at 30°C and 220 rpm for 24 h. Yeast cells were collected by centrifugation, resuspended in 5 ml of appropriate synthetic defined medium (Clontech) containing 2% galactose and then incubated at 30°C and 220 rpm for 48 h. In case of the PSIII strain, yeast strains with cell density at an OD_600_ value in the range of 1.4–1.6 (mid-logarithmic phase) were cultured in 5 mL appropriate synthetic defined medium (Clontech) containing 2% glucose, 100 nM β-estradiol, and 1 mM L-methionine and then incubated at 30°C and 220 rpm for 48 h. For the feeding assay, INV*Sc*1 yeast was cultured as described previously and supplemented with 10 μM of erythrodiol at the same time as galactose addition, and yeast were then cultured at 30°C and 220 rpm for 60 h.

### Metabolite Extraction

Before extraction, 20 ppm of ursolic acid or uvaol were added to 5 mL yeast culture as internal standards. Triterpenoid metabolite extraction was performed as previously reported (Fanani et al., 2019). The dried extract was dissolved in 500 μL of chloroform:methanol 1:1 (v/v). Upon Gas chromatography–mass spectrometry (GC-MS) analysis, 100 μL of the sample was evaporated to dryness and trimethylated with 50 μL of *N*-methyl-*N*-(trimethylsilyl) trifluoro acetamide (Sigma-Aldrich) for 30 min at 80°C.

### GC-MS Analysis

GC-MS was performed as previously reported (Fanani et al., 2019). For peak identification, authentic standards were used to confirm the peak in the sample and compare the retention time and mass fragmentation patterns. The relative amounts of triterpenoids in each strain were compared by calculating the area of each peak from the extracted chromatogram based on m/z values of 203 (Kim et al., 2018). Each sample of the different CYP-CPR combinations was analyzed using three biological replicates.

### Statistical Analysis

The significance of difference between two yeast strains harboring different CPR classes from same species was determined by single-factor analysis of variance (ANOVA). One-way ANOVA was used to test the difference of triterpenoid levels among different yeast strains and the significance for the means were separated by Tukey’s test. *p*-values less than 0.05 (*P* < 0.05) were considered to be significant for this study. All the statistical analyses were performed with SPSS 20.0 for Windows (IBM, Chicago, IL, United States).

## Results

### Phylogenetic and Structural Analysis of Plant CPRs

**FIGURE 2:**
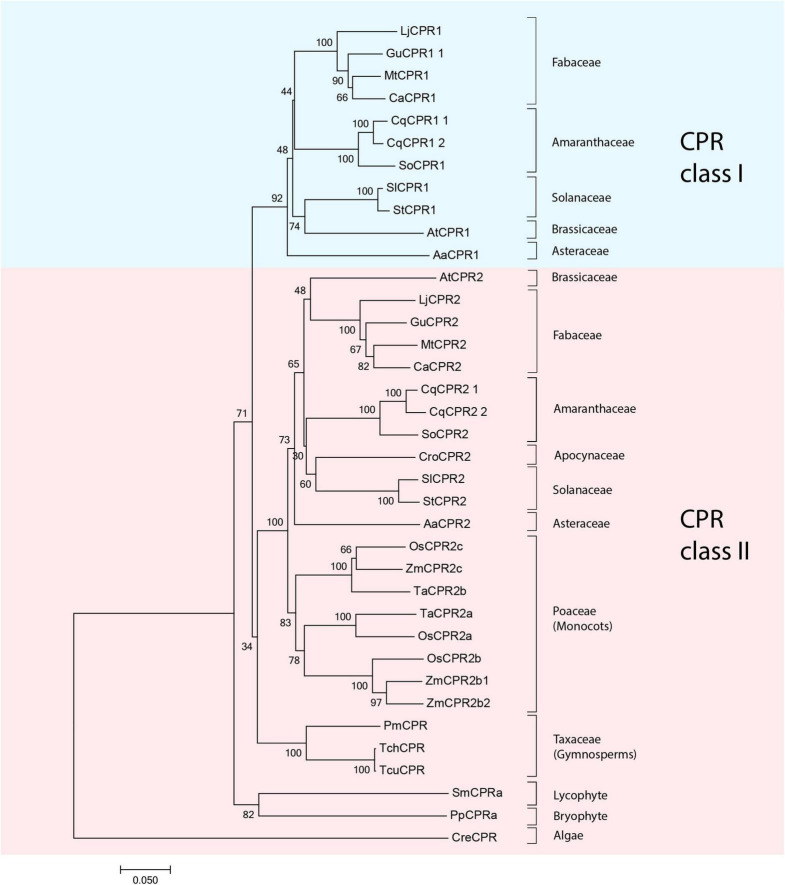
Molecular phylogenetic tree of 42 CPR amino acid sequences from 19 different plant species shows clear branching of class I (blue shade) and II (pink shade) CPRs in higher plants. Sequences of CPR classes I and II from *Medicago truncatula*, *Lotus japonicus*, and *Glycyrrhiza uralensis* that were used for further analysis in this study are indicated as black dots. CPR, cytochrome P450 reductase; Aa, *Artemisia annua*; ATR1, *Arabidopsis thaliana CPR1*; ATR2, *Arabidopsis thaliana CPR2*; Ca, *Cicer arietinum*; Cre, *Chlamydomonas reinhardtii*; Cro, *Catharanthus roseus*; Cq, *Chenopodium quinoa*; Gu, *Glycyrrhiza uralensis*; Lj, *Lotus japonicus*; Mt, *Medicago truncatula*; Os, *Oryza sativa*; Pm, *Pseudotsuga menziesii*; Pp, *Physcomitrella patens*; Sl, *Solanum lycopersicum*; Sm, *Selaginella moellendorffii*; St, *Solanum tuberosum*; Ta, *Triticum aestivum*; Tch, *Taxus chinensis*; Tcu, *Taxus cuspidata*; Zm, *Zea mays.*

The phylogenetic analysis results from 20 different plant species showed clear branching of CPR class I and II sequences (Figure 2). Each dicot plant species was found to have a minimum of one CPR sequence in each class. Monocots, gymnosperms, and mosses possess several copies of CPR-II genes, but no CPRs belonging to CPR class I have been found to date (Jensen and Møller, 2010). Amino acid sequences of plant CPRs from different plant families were compared to create a table of similarity matrices (Supplementary Table 3). Same CPR classes of the same plant family were found to have 75–95% similarity, while same CPR classes of different plant families were found to have 64–75% similarity. On the other hand, different CPR classes of the same or different plant families were found to have 55–65% similarity. Motif analysis was performed on different plant CPR classes (Supplementary Figure 2).

**FIGURE 3:**
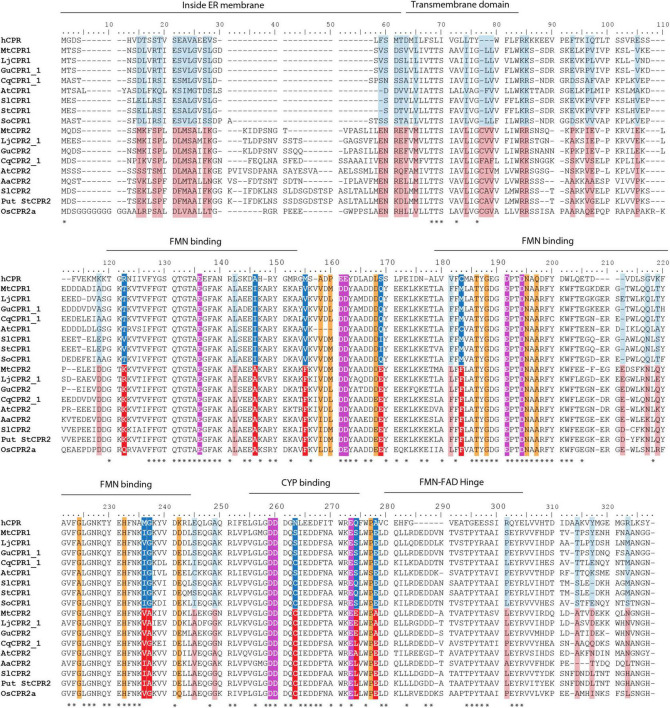
Multiple sequence alignment of first 327 amino acids sequence of CPR class I and II containing N-terminal and FMN domains using the ClustalX color scheme for amino acid alignments. Dark blue, red, light blue, and pink colors indicate the different conserved amino acids in CPR class I and II in legume family based on motif analysis (Supplementary Figure 2). Dark blue and red colors are the eight highlighted key residue in this study. Magenta color indicates acidic residue formerly reported to be important in CYP:CPR interaction in human CPR (hCPR). Orange color indicates point mutations in hCPR that are reported to improve interaction with a specific CYP. Asterisks indicate the positions which have a single, fully conserved residue. CPR, cytochrome P450 reductase; Aa, *Artemisia annua*; ATR1, *Arabidopsis thaliana CPR1*; ATR2, *Arabidopsis thaliana CPR2*; Ca, *Cicer arietinum*; Cq, *Chenopodium quinoa*; Gu, *Glycyrrhiza uralensis*; Lj, *Lotus japonicus*; Mt, *Medicago truncatula*; Os, *Oryza sativa*; Sl, *Solanum lycopersicum*; St, *Solanum tuberosum.*

**FIGURE 4:**
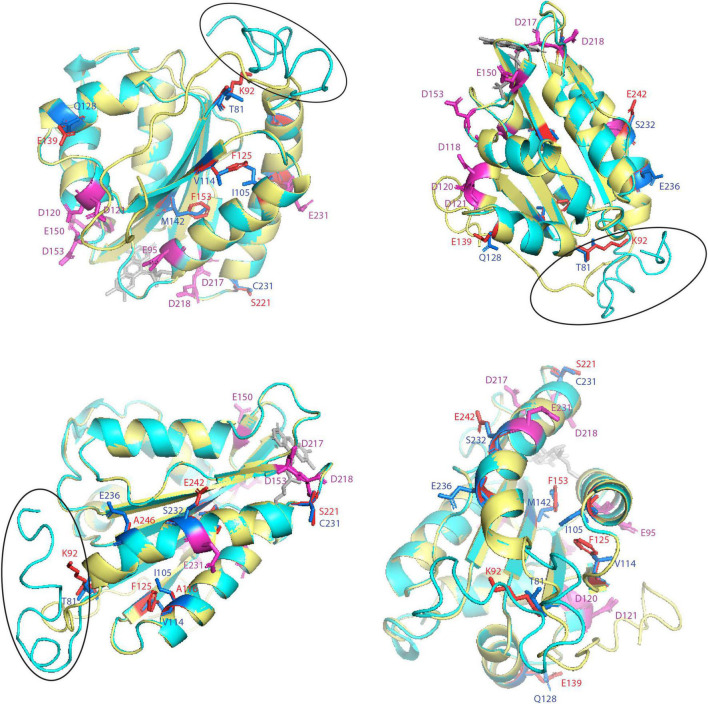
Structural analysis of key amino acid residues in the FMN-domain of MtCPRs model from four different angles. FMN-domain of MtCPR1 is colored in yellow and MtCPR2 in cyan. Specific residues are depicted as stick with the following color code: magenta for acidic residues formerly indicated to be involved in CPR:CYP interactions, blue for key residue in MtCPR1, and red for key residue in MtCPR2. The FMN molecule is colored in gray. Black circle indicates the FMN-FAD hinge.

Multiple sequence alignments from several plant CPRs showed that CPR-I has approximately 10–20 amino acids shorter N-terminal membrane sequences than CPR-II (Figure 3). In this study, we focused on the structural analysis of the FMN domain of legume CPRs using CPR from a model legume, MtCPR. Based on motif analysis of amino acids from CPR classes I and II from 24 legume species, there were some differences in the amino acid residues between CPR classes I and II in the FMN domain, which are conserved in each CPR class (Supplementary Figure 2). By multiple sequence alignment with hCPR, we highlighted eight amino acid residues of MtCPR1 and MtCPR2, which are co-localized with the position of hCPR mutations and negatively charged amino acid residues in the FMN domain. These amino acids have been reported to play a role in CPR:CYP-specific interactions (Campelo et al., 2018; Esteves et al., 2020; Figure 3). The location of each highlighted amino acid in the protein structure was observed through structural analysis of MtCPRs (Figure 4).

### Gene Co-expression Analysis of Legume CPR Classes I and II

**FIGURE 5:**
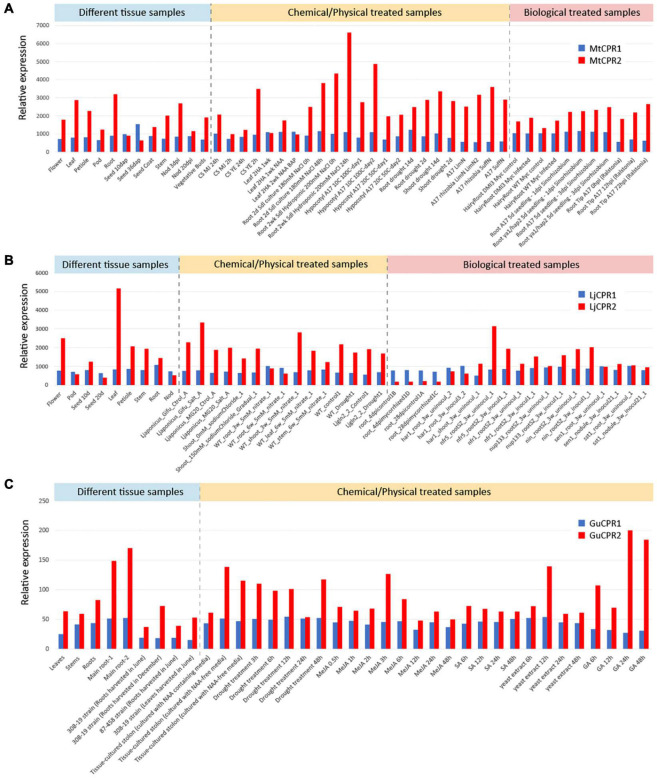
Co-expression analysis of CPR class I and II from **(A)** *M. truncatula*, **(B)** *L. japonicus*, and **(C)** *G. uralensis* using selected transcriptomic data-sets representing samples from different tissue, chemical/physical treatments, and biological treatments. Transcriptomic data-sets of *M. truncatula* and *L. japonicus* were obtained from https://mtgea.noble.org/v3/index.php and https://lotus.au.dk/ of MG-20 version 3.0. The *G. uralensis* transcriptomic data-set was obtained using RNA-seq analysis in this study.

We selected two model legumes, *Medicago truncatula* and *Lotus japonicus*, and one of the high-value medicinal legumes, *Glycyrrhiza uralensis*, to analyze the gene expression levels of different CPR classes in legumes. Available transcriptomic data of *M. truncatula* and *L. japonicus* were retrieved from the respective transcriptome database, while transcriptome data of *G. uralensis* were obtained in this study (Supplementary Tables 4.1–4.3). RNA-seq analysis from 37 data-sets of *G. uralensis* resulted in 351,138,706 reads and were assembled into 226,599 unigenes (Supplementary Table 5). Co-expression analysis of *CPR-I* and *CPR-II* genes of *M. truncatula*, *L. japonicus*, and *G. uralensis* showed distinct expression patterns (Figure 5). *CPR-I*s were found to be constitutively expressed with lower and more stable expression levels (Figure 5). In contrast, *CPR-IIs* were generally found to have higher expression levels than *CPR-Is*, which varied depending on tissues and treatments (Figure 5). Overall, *CPR-I* showed higher expression levels than that of *CPR-II* in roots or nodules, while CPR-II showed higher expression levels than of *CPR-I* in chemically or biologically treated samples (Supplementary Table 6). Global analysis of the genes from each legume species was performed by calculating the PCC of each CPR class with each of the transcripts found in the assembly (Supplementary Table 7). *CPR-I* showed strong correlation (PCC > 0.70) with genes involved in basal metabolism or molecular functions, such as ATP-binding/-carrier proteins (Supplementary Table 7). In contrast, *CPR-II* correlated strongly with receptor genes such as the ubiquitin, glutamate, and jasmonate receptors, which are known to play a role in plant defense responses (Guo et al., 2018; Qiu et al., 2020; Doroodian and Hua, 2021) (Supplementary Table 7). Interestingly, despite its critical role in providing electrons for CYPs, the global transcriptomic analysis of genes correlated with CPRs showed no correlation with CYPs, at a PCC > 0.4 (Supplementary Table 7).

In order to analyze the correlation strength between the different CPR classes and CYPs in each species, we calculated the PCC values of different CPR classes in combination with CYP families 716A, 72A, and 93E from the three legume species, in addition to CYP88D6 from *G. uralensis*. MtCYP716A12, LjCYP716A51, and GuCYP716A179 catalyze the same reaction of three steps of oxidation at the C-28 position of α-amyrin, β-amyrin, and lupeol to produce the final product of ursolic acid, oleanolic acid, and betulinic acid, respectively, (Fukushima et al., 2011; Tamura et al., 2017; Suzuki et al., 2019; Figure 1). Both MtCYP72A63 and GuCYP72A154 oxidize the C-30 position of β-amyrin to produce 30-OH β-amyrin, but only MtCYP72A63 catalyzes the further oxidation to produce 30-COOH β-amyrin (Seki et al., 2011). LjCYP72A61 catalyzes the one-step oxidation at the C-22 position of β-amyrin to produce sophoradiol (Ebizuka et al., 2011). MtCYP93E2, LjCYP93E1, and GuCYP93E3 catalyze the same reaction of three steps of oxidation on the C-24 position of β-amyrin to produce the final product of 24-COOH β-amyrin (Seki et al., 2008; Fukushima et al., 2011; Suzuki et al., 2019). GuCYP88D6 catalyzes the two-step oxidation at the C-11 position of β-amyrin to produce 11-oxo β-amyrin (Seki et al., 2008).

The PCC table shows that different CPR classes had different correlation values with different CYPs (Table 1). The probeset ID used for PCC calculation is presented in Supplementary Table 8. *CYP716A12* had a higher positive correlation value with *MtCPR1*, while *CYP72A63* had a higher positive value with *MtCPR2*. However, from the data-set, no significant difference was observed in the correlation of *MtCPRs* with *CYP93E2* (Table 1A). From the *L. japonicus* database MG20 v3.0, *LjCPR1* showed higher correlation values with *CYP716A51, CYP72A61*, and *CYP93E1*, as compared to *LjCPR2* (Table 1B). Based on the transcriptome data of *L. japonicus* in the Gifu version 2.0, *LjCPR1* also showed higher positive correlation value with *CYP716A51* and *CYP93E1*, as compared to *LjCPR2*. However, *CYP72A61* did not have strong correlation with any of the LjCPRs, but showed very high correlation value (>0.5) with *CYP93E1* (Supplementary Table 9). *GuCPR2* showed higher correlation with *CYP716A179*, *CYP72A154, and CYP93E3*, as compared to *GuCPR1.* In contrast, *CYP88D6* correlated negatively with *GuCPR1* (Table 1C).

**TABLE 1:** Correlation strength^a^ between different CPR classes and CYP families in **(A)** *M. truncatula*, **(B)** *L. japonicus*, and **(C)** *G. uralensis*.

(A)				
**Gene**	***MtCYP716A12***	***MtCYP72A63***	***MtCYP93E2***	
*MtCPR1*	0.12	0.22	0.25	
*MtCPR2*	–0.03	0.41	0.17	

**(B)**				

**Gene**	***LjCYP716A51***	***LjCYP72A61***	***LjCYP93E1***	

*LjCPR1*	0.54	0.59	0.70	
*LjCPR2*	–0.35	–0.35	–0.29	

**(C)**				

**Gene**	***GuCYP716A179***	***GuCYP72A154***	***GuCYP93E3***	***GuCYP88D6***

*GuCPR1*	–0.23	–0.33	0.34	–0.49
*GuCPR2*	0.33	0.29	0.70	–0.02

*^a^Correlation strength of different CPR classes with different CYP families based on PCC. PCC values range from −1 to 1. Positive values were positively correlated, whereas negative values were negatively correlated. PCC, Pearson’s correlation coefficient; Mt, Medicago truncatula; Lj, Lotus japonicus; Gu, Glycyrrhiza uralensis.*

### CYP Shows Preference Toward Certain CPR Class

**FIGURE 6:**
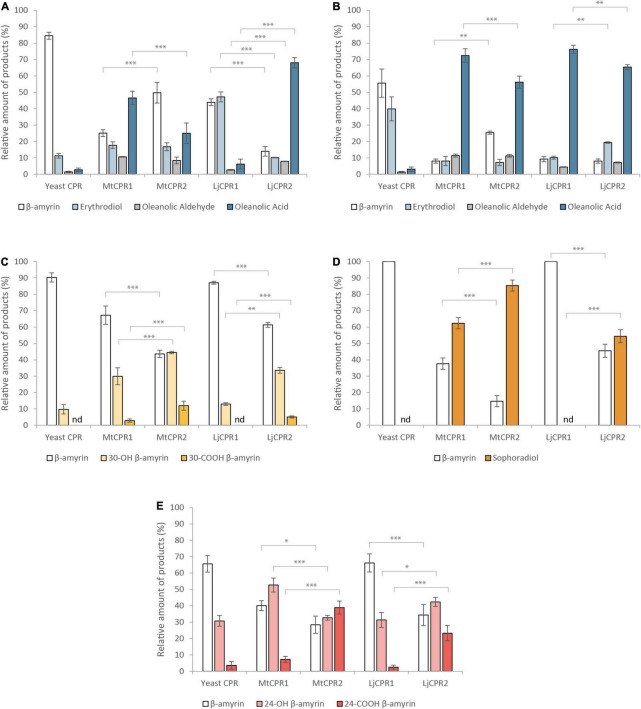
The relative amount of triterpenoid produced using different combinations of CYPs and CPRs from *M. truncatula* and *L. japonicus* in β-amyrin-producing yeast (INV*Sc1* strain). Triterpenoid profiles from **(A)** MtCYP716A12, **(B)** LjCYP716A51, **(C)** MtCYP72A63, **(D)** LjCYP72A61, and **(E)** LjCYP93E1 co-expressed with different CPRs in INV*Sc1*. Triterpenoids content were measured relative to uvaol as internal standard. Data have been presented as mean ± SE (*n* = 3). nd, signal below detection limit. Single-factor ANOVA was used for statistical comparisons. Values were considered statistically significant at *P < 0.05, **P < 0.01, and ***P < 0.001.

**FIGURE 7:**
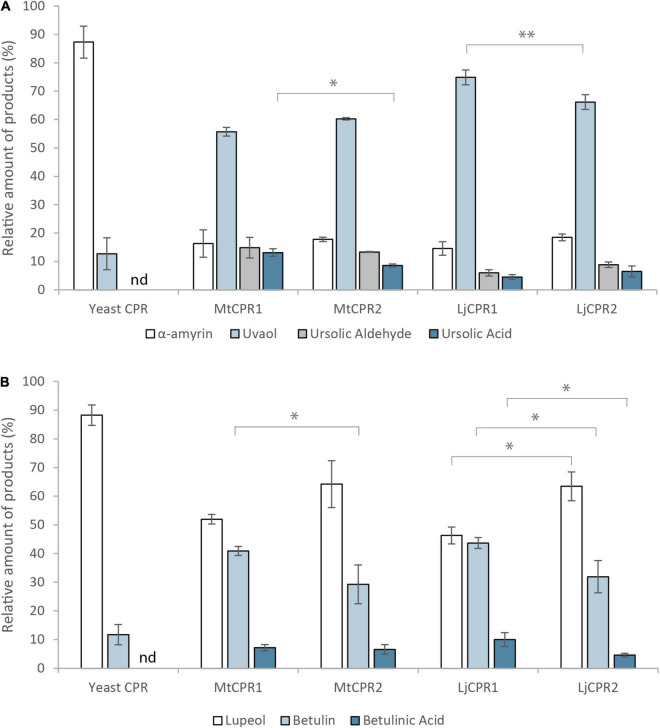
The relative amount of triterpenoid produced by co-expressing MtCYP716A12 with different CPRs from *M. truncatula*, *L. japonicus*, and *G. uralensis* in **(A)** α-amyrin-producing and **(B)** lupeol-producing yeast (INV*Sc1* strain). Triterpenoids content were measured relative to erythrodiol as internal standard. Data have been presented as mean ± SE (*n* = 3). nd, signal below detection limit. Single-factor ANOVA was used for statistical comparisons. Values were considered statistically significant at *P < 0.05 and **P < 0.01.

To analyze the effect of different CPR classes on heterologous triterpenoid production, different CYPs and CPR classes from *M. truncatula* and *L. japonicus* were co-expressed in transgenic *S. cerevisiae* INV*Sc*1, a commonly used strain for recombinant protein expression. By comparing the relative value of each metabolite using a semi-quantitative method with an internal standard, we compared the accumulation of oxidized triterpenoids resulting from the heterologous expression of different CPR-CYP pairs in transgenic yeast (Figure 6). The amount of triterpenoid backbone in the control samples was considered to be 100%. In yeast strains expressing MtCYP716A12 and LjCYP716A51, the intermediate compounds oleanolic and ursolic aldehyde could be detected based on the mass spectrum profile (Supplementary Figure 3) compared to previously reported mass spectra of oleanolic and ursolic aldehyde authentic standards (Misra et al., 2017). However, betulinic aldehyde could not be detected in the yeast extract and was co-eluted with betulinic acid in the yeast extract; (Suzuki et al., 2018) therefore, the amount of betulinic acid shown in Figure 7 corresponds to the sum of both compounds. C-30 and C-24 aldehydes were excluded because of the instability of the compound, lack of authentic standards, and no previous reports on their mass spectrum profile.

The results show that upon using INV*Sc*1 β-amyrin-producing yeast, MtCYP716A12 and LjCYP716A51 showed higher relative amount of oleanolic acid when paired with MtCPR1, as compared to when they were paired with MtCPR2, with a comparable amount of erythrodiol and oleanolic aldehyde (Figures 6A,B). Pairing MtCYP716A12 with LjCPR1 showed higher conversion of β-amyrin into erythrodiol compared to pairing it with LjCPR2. However, pairing MtCYP716A12 with LjCPR2 showed a higher conversion rate of erythrodiol into oleanolic aldehyde and oleanolic acid than with LjCPR1 (Figure 6A). Conversely, pairing LjCYP716A51 with LjCPR2 resulted in a higher conversion rate of β-amyrin into erythrodiol than with LjCPR1, while the conversion rate of erythrodiol into oleanolic acid was higher when pairing LjCYP716A51 with LjCPR1 than LjCPR2 (Figure 6B). These trends were also similar in the yeast feeding assay results of INV*Sc*1 strain supplemented with erythrodiol as a substrate, although the difference was not as significant as in the *in vivo* assay (Supplementary Figure 4). In contrast, pairing MtCYP72A63 with CPR-II resulted in significantly higher conversion of β-amyrin into 30-OH β-amyrin and 30-COOH β-amyrin levels (Figure 6C) than pairing it with CPR-I. Similar to MtCYP72A63, pairing LjCYP72A61 with CPR-IIs resulted in significantly higher conversion of β-amyrin into sophoradiol (Figure 6D). In case of the legume-specific CYP subfamily, CYP93E1 paired with CPR-II showed higher conversion of β-amyrin into 24-COOH β-amyrin, as compared to CYP93E1 paired with CPR-I (Figure 6E).

CYP716As can also catalyze oxidations on different triterpenoid backbone. Therefore, to confirm that the effect of the CPR on the conversion rate can also be applied to different triterpenoid backbone, we then co-expressed MtCYP716A12 in α-amyrin and lupeol-producing strains (Figure 7). The result showed that MtCYP716A12 paired with MtCPR1 and LjCPR1 showed higher conversion of α-amyrin into ursolic acid and uvaol, respectively (Figure 7A). A similar trend was observed in the lupeol-producing strain in which MtCYP716A12 paired with MtCPR1 showed a higher conversion rate of betulin into betulinic acid compared to that with MtCPR2, and MtCYP716A12 paired with LjCPR1 showed a higher conversion rate of lupeol to betulin and betulinic acid compared to LjCPR2 (Figure 7B). All the chromatograms of yeast *in vivo* are shown in Supplementary Figures 5–14.

This yeast *in vivo* assay might provide insight into CPR-CYP preferences, which are better justified by their protein expression levels. Quantifying the CPR protein itself would not be sufficient because triterpenoids are the products of CYP. However, protein quantification of CYP remains challenging. We propose this study to analyze the effect of CPR class on heterologous production in yeast, but not on the catalytic activity of CPR or CYP. We admit that this is a limitation of yeast *in vivo* studies. Therefore, further in-depth *in vitro* analysis of the effect of CPR class on CYP activity might be needed in future experiments to provide more justifiable results.

### N-Terminal Domain Swapping of MtCPR1 and MtCPR2

Based on the multiple sequence alignment result, the main difference of CPR class I and II is located in the N-terminal domain. CPR class I has shorter amino acid sequence than CPR class II (Figure 3). To analyze whether this N-terminal domain is responsible for the differences in the conversion rate of β-amyrin when CPR was co-expressed with different CYPs in transgenic yeast (Figure 6), we investigated the effect of this domain by swapping the N-terminal membrane sequence of MtCPR1 and MtCPR2. MtCPRs were used as the representative since *M. truncatula* is a model legume. We speculated if the N-terminal domain of CPR is indeed responsible for the CPR activity, swapping the N-terminal domain of CPR class I and II will affect the triterpenoids profile.

**FIGURE 8:**
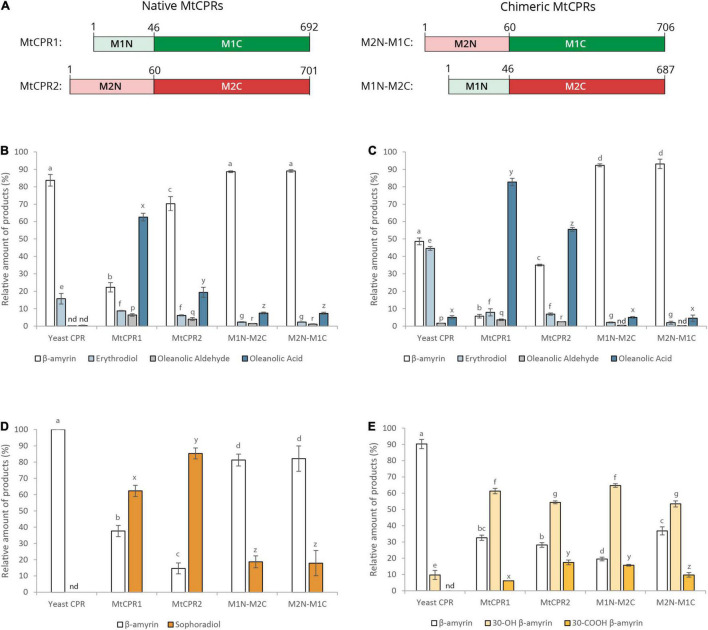
The relative amount of triterpenoid produced by co-expressing different CYPs with N-terminal-swapped chimeric CPRs. **(A)** N-terminal domain of MtCPR classes I and II were swapped to generate two chimeric CPRs, M2N-M1C, and M1N-M2C. The numbers indicate the position of amino acid residue. These chimeric CPRs were then co-expressed with **(B)** MtCYP716A12, **(C)** LjCYP716A51, **(D)** LjCYP72A61, and **(E)** MtCYP72A63 in β-amyrin-producing yeast (INV*Sc1* strain). Triterpenoids content were measured relative to uvaol as internal standard. Data have been presented as mean ± SE (*n* = 3). nd, signal below detection limit. Single-factor ANOVA was used for statistical comparisons. The different letters indicate significant differences (*P* < 0.05, one-way ANOVA followed by Tukey’s test). Letter a–d are used to indicate significancy for β-amyrin, letter e–g are for erythrodiol and 30-OH β-amyrin, letter p–r are for oleanolic aldehyde, and letter x–z are for oleanolic acid, sophoradiol, and 30-COOH β-amyrin.

The location of transmembrane helix of MtCPR1 and MtCPR2 was predicted *in silico* (Supplementary Figure 1). Amino acids of MtCPR1 number 1–26 are located inside the ER, number 27–46 are the transmembrane helix, and number 47–692 are located outside the ER (Supplementary Figure 1A). Amino acids of MtCPR2 number 1–40 are located inside the ER, number 41–60 are the transmembrane helix, and number 61–701 are located outside the ER (Supplementary Figure 1B). In this study, the first 46 amino acids of MtCPR1 located inside the ER together with the transmembrane helix were swapped with the first 60 amino acids of MtCPR2. The resulting chimeric MtCPRs were N-terminal membrane sequence of MtCPR1 fused with truncated-N-terminal MtCPR2 (M1N-M2C) and N-terminal membrane sequence of MtCPR2 fused with truncated-N-terminal MtCPR1 (M2N-M1C) (Figure 8A).

Yeast *in vivo* assay was then performed by co-expressing the chimeric MtCPRs with CYP716As and CYP72As to observe its effect on triterpenoids conversion rate (Figures 8B–E). The results showed that by swapping the N-terminal domain of CPR class I with that of class II, the product from CYP716A12 and CYP716A51 were significantly reduced (Figures 8B,C). The production of erythrodiol by CYP716A51 co-expressed with mutant CPRs was even lower than the background level observed in native yeast CPR (Figure 8C). A similar effect was also seen in CYP72A61, where the chimeric CPR caused a significant decrease in conversion of β-amyrin into sophoradiol (Figure 8D). Interestingly, only CYP72A63 was not affected by the chimeric MtCPRs. Moreover, the results showed that CYP72A63 paired with the N-terminal membrane sequence of MtCPR2 fused with truncated N-terminal MtCPR1 (M2N-M1C) showed higher conversion of β-amyrin into 30-OH β-amyrin and 30-COOH β-amyrin (Figure 8E).

### Improving Triterpenoid Production Using an Engineered Yeast

**FIGURE 9:**
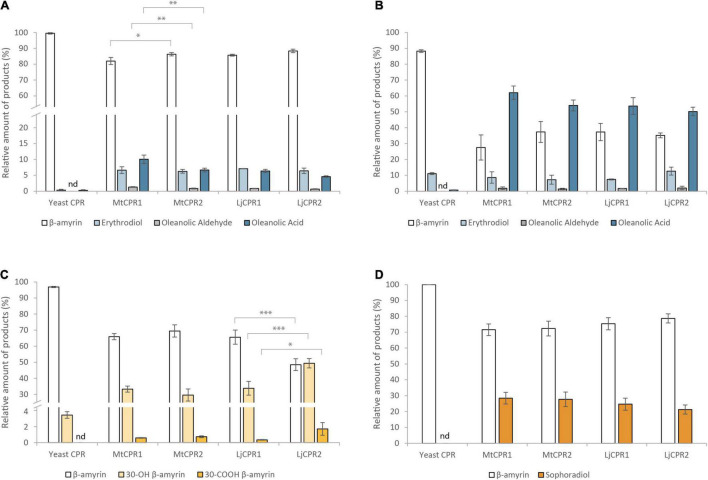
The relative amount of triterpenoid produced using different combinations of CYPs and CPRs from *M. truncatula* and *L. japonicus* in the engineered PSIII yeast strain. Triterpenoid profiles from **(A)** *MtCYP716A12*, **(B)** *LjCYP716A51*, **(C)** *MtCYP72A63*, and **(D)** *LjCYP72A61* co-expressed with different CPRs. Triterpenoids content were measured relative to uvaol as internal standard. Data have been presented as mean ± SE (*n* = 3). nd, signal below detection limit. Single-factor ANOVA was used for statistical comparisons. Values were considered statistically significant at *P < 0.05, **P < 0.01, and ***P < 0.001.

To further analyze the effect of different CPR classes on improving heterologous production, different CPR classes were co-expressed with different CYP families in the PSIII strain. PSIII is an engineered yeast strain that has been optimized for triterpenoid biosynthesis by means of some modifications in the upstream mevalonate pathway. This strain is modified from the PSII strain constructed in previous research, which can produce a 100 × times higher triterpene backbone as a substrate than its parental strain (Srisawat et al., 2020). In contrast to the INV*Sc*1 results, in the PSIII strain, almost all the triterpenoid sapogenin production profiles had a lower ratio of β-amyrin, and the trends of CYP-CPR preferences observed in the INV*Sc*1 strain seem to be less obvious in this strain (Figure 9). Nevertheless, some notable changes were observed in the PSIII strain. MtCYP716A12 paired with LjCPR1 that previously showed different conversion ratios of triterpenoids with LjCPR2 in the INV*Sc*1 strain, showed similar conversion ratio profiles in the PSIII strain (Figure 9A). The ratio of oleanolic acid to erythrodiol was lower in PSIII strain co-expressing MtCYP716A12 (Figure 9A). The PSIII strain co-expressing MtCYP72A63 resulted in much lower 30-COOH β-amyrin to 30-OH β-amyrin ratio, as compared to the INV*Sc*1 strain (Figure 9C). Co-expressing all CPRs with LjCYP72A61 and MtCYP716A51 resulted in similar conversion ratio in PSIII strain (Figure 9D). However, similar to the INV*Sc*1 strain, co-expressing MtCYP716A12 with MtCPR1 in the PSIII strain showed higher oleanolic aldehyde and oleanolic acid conversion rates than MtCPR2. In addition, co-expressing MtCYP72A63 with LjCPR2 in the PSIII strain showed higher 30-OH β-amyrin and 30-COOH β-amyrin conversion rates compared to that with LjCPR1.

**FIGURE 10:**
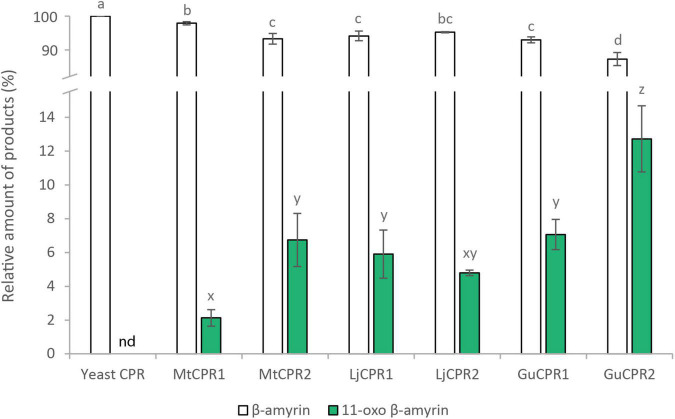
Improving 11-oxo-β-amyrin production using the engineered PSIII yeast strain. The relative amount of triterpenoid in PSIII strain co-expressing different combinations of GuCYP88D6 with different CPRs. 11-oxo-β-amyrin content was measured relative to an internal standard (uvaol). Data have been presented as mean ± SE (*n* = 3). nd, signal below detection limit. The different letters indicate significant differences (*P* < 0.05, one-way ANOVA followed by Tukey’s test). Letter a–d and x–z are used to indicate significancy for β-amyrin and 11-oxo-β-amyrin content, respectively.

Co-expression of different CYP and CPR classes from model legumes, *Medicago truncatula* and *Lotus japonicus*, have suggested that CYP shows a preference for certain CPR classes. Therefore, to further study the effect of CYP-CPR pairs, we added CPRs from a non-model legume species, *Glycyrrhiza uralensis*, which produces glycyrrhizin, a highly valuable metabolite found only in Glycyrrhiza sp. Furthermore, unlike CYP716As and CYP72As, which are commonly found in plant species, GuCYP88D6 uniquely presents in *Glycyrrhiza* sp. and is very specific in function. Therefore, it is interesting to facilitate contrasting results. GuCYP88D6 catalyzes the two-step oxidation at the C-11 position of β-amyrin to produce 11-oxo β-amyrin, the precursor for glycyrrhizin (Seki et al., 2008). The PCC value of *GuCYP88D6* with *GuCPRs* also indicates that *GuCYP88D6* might have different preferences for *GuCPR* classes I and II, which shows a negative correlation with *GuCPR1* (Table 1C). Corresponding to this hypothesis, the results showed that co-expressing GuCYP88D6 with class II GuCPR in the PSIII strain showed the highest conversion of β-amyrin into 11-oxo β-amyrin, as compared to the other CPRs tested in this study (Figure 10).

## Discussion

Based on the phylogenetic results of plant CPRs, it was identified that dicotyledons possess both CPR-I and CPR-II; thus far, only CPR-II genes have been identified in monocotyledons, gymnosperms, and mosses. Among angiosperms, as compared to dicotyledons, monocotyledons are well known to be more tolerant to abiotic stresses (Agrawal and Agrawal, 1999; Rao et al., 2006; Wright et al., 2010). CPR-II is believed to play a more important role in defense and adaptation mechanisms compared to CPR-I (Rana et al., 2013; Parage et al., 2016). The fact that multiple copies of CPR-II were found in all monocotyledons, while some dicotyledons might only possess a single CPR-II (Figure 2; Jensen and Møller, 2010), suggest that the role of CPR-II is very prominent in supporting abiotic resistance in monocots. In addition, the more sophisticated translocation and root system of dicotyledons (Scarpella and Meijer, 2004) might be supported by the presence of CPR-I found in dicotyledons. Nevertheless, it remains to be determined whether monocots possess only CPR-II. However, this might also explain why the expression of CPR-I is more abundant in roots and nodules with higher meristematic activity, while the expression of CPR-II is higher in leaf, shoot, and treated plants (Supplementary Table 4.4). Global co-expression analysis results also support this deduction, that CPR-I supports primary or basal metabolism, while CPR-II plays a more important role in defense mechanisms (Supplementary Table 5).

The FMN domain of CPRs has several conserved patches of acidic residues located on its solvent-exposed surface, suggesting that they are involved in the electrostatic interaction with its redox partners, including CYPs (Waskell and Kim, 2015). It was shown that in humans, every CYP interacts with CPR in a specific manner, based on the difference in the binding motifs located in the FMN domain (Esteves et al., 2020). The motif analysis of 24 different legume species showed that there are some differences in the conserved amino acid residues between legume CPR classes I and II in the FMN domain. Among them, eight residues either located on the conserved binding domain or near the previously suggested residue to be important for CYP:CPR interactions in humans were highlighted. CPR from a model legume, MtCPRs, was used for 3D structural analysis. The first highlighted amino acids, T81 in MtCPR1 and K92 in MtCPR2, are located on the first residue in the FMN binding domain (Figure 4). This difference in the residues is conserved in CPR I and II of legumes and other species. Based on the structural analysis, these residues were located near the FMN-FAD hinge (Figure 4). Interflavin electron transfer (ET) has been previously reported to be the rate-limiting step in plant CPR (Simtchouk et al., 2013; Whitelaw et al., 2015). This highly flexible hinge also has been suggested to play a role in the formation of multiple open conformations to form complexes with CYP in an isoform-dependent manner (Campelo et al., 2018). Therefore, the difference in size and charge properties of T81 in MtCPR1 and K92 in MtCPR2 might greatly affect the conformational flexibility of the interflavin hinge during ET. The next highlighted residues are I105, V114, and M142 in MtCPR1 and A116, F125, and F153 in MtCPR2 (Figure 4). Even though these amino acid residues are all hydrophobic and only exhibit small differences in the chain length, these different residues of MtCPR1 and MtCPR2 are conserved in CPR I and II of legumes and other plant families (Figure 3). These residues are located in the α-helix of the FMN-binding domain, are close to the acidic residues important for ET, and face each other (Figure 4). The difference in these residues might cause minor changes in the geometrical shape of the α-helix of the FMN-binding domain, leading to improved interactions with specific CYPs, in which small conformational perturbations of the α-carbon backbone might accommodate changes in substrate binding (Bart and Scott, 2018). One of the most important residues is Q128 in MtCPR1 and E139 in MtCPR2 (Figure 4), which are also conserved in legumes and in other plant families (Figure 3). These residues are located in the acid-rich residues on the solvent-exposed surface, which are highly likely to serve as binding motifs for CPR:CYP interactions (Figure 4). The glutamate (E139) residue in CPR II provides a stronger negative charge to form ionic bonds with the respective CYP. Changing this to an uncharged glutamine residue, as in CPR I (Q128), will greatly affect the CYP interaction. The last three highlighted residues are S221, S232, and E236 in MtCPR1 and C231, E242, and A246 in MtCPR2 (Figure 4). Interestingly, these residues seem to be conserved only in CPR I and II of the legume family (Figure 3 and Supplementary Figure 2). These residues are located on the solvent-exposed surface of the FMN domain and near the acidic residue important for CPR:CYP interaction (Figure 4). C231 in MtCPR2 can form disulfide bridges with the interacting CYP, which cannot be achieved by S221 in MtCPR1. Serine-to-cysteine mutations have also been reported to have stabilizing effects because of optimization of van der Waals interactions and increased packing (Santos et al., 2007). Hence, C231 in CPRII might account for more stable interactions with specific CYPs, compared to that of CPRI. The last two residues are acidic residues of E242 and A246 in MtCPR2 and S232 and E236 in MtCPR1. The difference between the negatively charged glutamate (E) residue and short aliphatic alanine (A) or uncharged serine residue (S) greatly affects the ionic interaction between CPR and its ET partner. The positions of the acidic residues in CPR I and II are different but parallel in the α-helix (Figure 4), which is highly likely to lead to CYP isoform-specific interactions. The fact that these positions are conserved only in legume CPRs suggests that CPR from each plant family might exhibit unique CYP binding motifs that interact with specific CYPs.

This study emphasizes that different CYPs show a preference for certain CPR classes. Based on the yeast *in vivo* assay in the INV*Sc*1 β-amyrin-producing strain, CYP716As paired with CPR-I generally showed a higher conversion ratio of triterpenoid backbone when they were paired with CPR-II, except that MtCYP716A12 paired with LjCPR1 showed a lower conversion ratio of triterpenoids compared to LjCPR2 (Figure 6). This might be due to the incompatibility of CYP716A12 from *M. truncatula* with LjCPR1 owing to species differences. However, the preference of CPR-I over CPR-II of MtCYP716A12 was also observed in the INV*Sc*1 α-amyrin and lupeol-producing strains (Figure 7). On the other hand, CYP72A63, CYP72A61, and CYP93E1 showed higher conversion ratio when co-expressed with CPR-II (Figure 6). It has previously been reported that the N-terminal of ATR2 (*Arabidopsis thaliana* CPR2), which contains high number of Ser/Thr residues, significantly enhances the ATR2 activity (Urban et al., 1997), which might lead to higher conversion rate. However, N-terminal domain swapping of MtCPR1 and MtCPR2 showed that the longer N-terminal membrane sequence of MtCPR2 did not result in increase of conversion rate of β-amyrin. In contrast, these chimeric CPRs caused a reduction in the β-amyrin conversion rate of the CYP716As and CYP72A61, while only showed little or no effect when co-expressed with CYP72A63. Interestingly, chimeric CPR M2N-M1C showed a higher conversion ratio of 30-COOH β-amyrin than native MtCPR1, implying that the N-terminal domain of MtCPR2 might correspond to an increase in the conversion ratio. This result suggests that there might be a specific CYP-CPR protein-protein interaction that was affected by the sequence-structural relationship in the N-terminal domain of CPRs. A previous study on human CPR-FMN-domain mutants has also shown that different CYP isoforms interact with CPR in a specific manner due to docking elements in the binding motifs of the FMN-domain, which affect the CYP-CPR affinity (Ritacco et al., 2019). The N-terminal domain swapping between MtCPR1 and MtCPR2 might cause a structural change in the CYP:CPR-specific binding motif, as shown in Figure 3. Further studies, such as mutagenesis experiments on CPR, are needed to understand the mechanisms underlying this phenomenon.

There are several aspects that might affect heterologous gene expression between INV*Sc*1 and PSIII strain. The INV*Sc*1 strain is a diploid, whereas BY4742, the parental strain of PSIII, is a haploid strain. Diploid yeast co-expressing similar plasmids resulted in more stable and approximately two times higher copy number than the haploid strain (Karim et al., 2013). The engineered pathway of PSIII strain also significantly affects triterpenoid production. Benefiting from the GEV chimeric transcriptional activator system, this strain can utilize glucose as a carbon source, while the triterpenoid biosynthetic genes are being activated by β-estradiol (McIsaac et al., 2011). Meanwhile, INV*Sc*1 utilizes galactose as its growth medium to active GAL promoters, which presents the drawback of catabolic repression in the presence of glucose (Escalante-Chong et al., 2015). Upon growing in glucose, the PSIII strain exhibited a much higher growth rate than INV*Sc*1, which might affect its ability to produce triterpenoids. One of the factors affecting enzyme activity is substrate concentration (Zhu et al., 2018). In the PSIII strain, the abundant β-amyrin concentration might drive the CYP-CPR pairs, which otherwise show lower conversion ratio of β-amyrin in the INV*Sc*1 strain. The fact that CYP716As and CYP72As are both widespread in plants and possess diverse functions in triterpenoid site-specific oxidations might be the reason they work well with all CPR classes in the engineered yeast strain.

Despite many variables affecting gene expression and metabolic regulation in yeast, this study achieved the highest conversion of β-amyrin into 11-oxo-β-amyrin by pairing *GuCYP88D6* with *GuCPR2*. Previous research has attempted to screen different CPRs for glycyrrhetinic acid production and reported that *GuCPR1* is the best fit for *CYP88D6* (Zhu et al., 2018). However, this study revealed that pairing *CYP88D6* with *GuCPR2* showed 1.8 times higher conversion ratio of β-amyrin into 11-oxo-β-amyrin than pairing with *GuCPR1.* This result also reveals that the conversion of β-amyrin in PSIII is still inefficient, as compared to the INV*Sc*1 strain, as shown in the lower ratio of the oxidized products of β-amyrin. This implies that there is still an opportunity to improve triterpenoid production in yeast by increasing the conversion efficiency of β-amyrin.

In conclusion, this study revealed that pairing different plant CYP families with different plant CPR classes results in different triterpenoid conversion ratio in heterologous yeast. This is the first study to compare different pairs of CPR classes and CYPs from legumes in transgenic yeast. The comparison data of this study did not cover enough CPR-CYP pairs from different species to generalize which CPR class is better for which CYP. Nevertheless, this study highlighted that CYP does have a preference for certain CPR classes, rejecting the previous notion that CPR-II is responsible for specialized metabolism. A strategy that could be proposed through this study is that performing screening for both CPR classes from the same species as their target CYPs would increase the chance of finding the best CPR pair for their heterologous production system. This study cannot be generalized to other species, but at least for legume species, the CYP72A family has a higher chance of working better with CPR class II. Furthermore, CYP716A has a higher probability of working better with CPR class I of the same species. Initiating research based on this fact might help other researchers find the best CPR pairs.

However, the underlying mechanisms of how a CPR prefers a specific CYP have not yet been fully elucidated. Therefore, more CPRs and CYPs from different plant families need to be studied in order to gain a deeper understanding of CPR class preferences toward CYPs. *In planta* and *in vitro* enzyme studies are necessary to further analyze the mechanisms of CYP-CPR interactions. Nevertheless, the observation that legume CPR classes support different CYPs in the heterologous production of triterpenoids in transgenic yeast may facilitate research on improving heterologous production of other beneficial compounds by co-expressing the proper CPR with the target CYPs.

## Data Availability Statement

Raw RNA-Seq reads of *G. uralensis* obtained in this study have been submitted to the DNA Data Bank of Japan (DDBJ) Sequence Read Archive (DRA) under the accession numbers of DRA012266 and run accession numbers as listed in Supplementary Table 10.

## Author Contributions

PI, EF, and TM designed the experiments. PS constructed the PSIII yeast strain. KT, AC, HSu, and HSe conducted experiments on RNA-sequencing. PI conducted the rest of the experiments, analyzed the results, and wrote the whole manuscript. EF, SY, HSe, and TM conceived and supervised the study. PS, SY, HSe, and TM made the manuscript revisions.

## Conflict of Interest

The authors declare that the research was conducted in the absence of any commercial or financial relationships that could be construed as a potential conflict of interest.

## Publisher’s Note

All claims expressed in this article are solely those of the authors and do not necessarily represent those of their affiliated organizations, or those of the publisher, the editors and the reviewers. Any product that may be evaluated in this article, or claim that may be made by its manufacturer, is not guaranteed or endorsed by the publisher.

## Footnotes

1
www.P450.kvl.dk

2
https://www.ncbi.nlm.nih.gov/

3
http://www.cbs.dtu.dk/services/TMHMM/

4
http://meme-suite.org
