# Supplementary material for: Comparative Analysis of NADPH-Cytochrome P450 Reductases From Legumes for Heterologous Production of Triterpenoids in Transgenic Saccharomyces cerevisiae
Source: Front Plant Sci. 2021 Dec 16;12:762546. doi: 10.3389/fpls.2021.762546 (PMC8716914; doi:10.3389/fpls.2021.762546)
Supplement: Supplementary file 1 [file Data_Sheet_1.pdf]

Supplementary Data

Supplementary Method

List of tables

Table S1. List of accession numbers of CPR genes used for phylogenetic analysis

Table S2. Primer sequences used in this study

Table S3. Similarity matrix of amino acid sequences of different CPR classes from different plant families

Table S4. Co-expression analysis of CPR class I and II of *M. truncatula*, *L. japonicus*, and *G. uralensis*

Table S5. Co-expression analysis of closely correlated genes with CPR class I and II in different tissues of a) *M. truncatula*, b) *L. japonicus*, and c) *G. uralensis*

Table S6. Correlation strength between different CPR class and CYP families in *L. japonicus* based on Gifu v2.0 genome version

List of figures

Figure S1. Transmembrane helix prediction of MtCPR1 and MtCPR2

Figure S2. Motif analysis of FMN domain of legume CPR class I and II

Figure S3. Mass spectra of target compounds and authentic standards

Figure S4. Triterpenoid production in yeast INV*Sc*1 strain supplemented with erythrodiol

Figure S5. GC-MS chromatogram of triterpenoids extracted from β-amyrin-producing INV*Sc1* yeast harboring *MtCPRs* and *LjCPRs* paired with A) *CYP716A12* and B) *CYP716A51*

Figure S6. GC-MS chromatogram of triterpenoids extracted from β-amyrin-producing INV*Sc1* yeast harboring *MtCPRs* and *LjCPRs* paired with *CYP716A12* and *CYP716A51*

Figure S7. GC-MS chromatogram of triterpenoids extracted from β-amyrin-producing INV*Sc1* yeast harboring *MtCPRs* and *LjCPRs* paired with *CYP72A63*

Figure S8. GC-MS chromatogram of triterpenoids extracted from β-amyrin-producing INV*Sc1* yeast harboring *MtCPRs* and *LjCPRs* paired with *CYP72A61*

Figure S9. GC-MS chromatogram of triterpenoids extracted from β-amyrin-producing PSIII yeast harboring *MtCPRs* and *LjCPRs* paired with *CYP716A12*

Figure S10. GC-MS chromatogram of triterpenoids extracted from β-amyrin-producing PSIII yeast harboring *MtCPRs* and *LjCPRs* paired with *CYP716A51*

Figure S11. GC-MS chromatogram of triterpenoids extracted from β-amyrin-producing PSIII yeast harboring *MtCPRs* and *LjCPRs* paired with *CYP72A63*

Figure S12. GC-MS chromatogram of triterpenoids extracted from β-amyrin-producing PSIII yeast harboring *MtCPRs* and *LjCPRs* paired with *CYP72A61*

Figure S13. GC-MS chromatogram of triterpenoids extracted from β-amyrin-producing PSIII yeast harboring *MtCPRs*, *LjCPRs*, and *GuCPRs* paired with *CYP88D6*

Figure S14. GC-MS chromatogram of triterpenoids extracted from INV*Sc1* yeast strain harboring *MtCPRs*, *LjCPRs*, and *GuCPRs* paired with *CYP716A12 and CYP716A51* supplemented with 10μM erythrodiol

Supplementary Method

1. RNA sequencing of *Glycyrrhiza uralensis*

1.1. *G. uralensis* sample preparations

1.1.1. Treatment of tissue-cultured stolons with drought

Tissue-cultured stolons of *G. uralensis* (Hokkaido-iryodai strain) were maintained in Murashige and Skoog (MS) medium (Duchefa Biochemie) supplemented with 6% sucrose and 0.01 mM 1-naphthaleneacetic acid (NAA) as reported previously (Kojoma *et al*. 2010). Tissue-cultured stolons were cultured for 2 weeks in MS medium supplemented with 6% sucrose without NAA before treatment (L6 and L7). For drought treatment, the culture stolon was removed from the liquid medium, and the medium on the surface of the cultured stolon was lightly wiped with paper towel, then incubated for 3 – 48 hours (L8-L12).

1.1.2. Treatment of tissue-cultured stolons with plant hormones or yeast extract elicitor

Tissue-cultured stolons were cultured for 2 weeks in MS medium supplemented with 6% sucrose without NAA before treatment. Tissue-cultured stolons were treated with plant hormones such as Methyl jasmonate (MeJA, L13-L20), Salicylic acid (SA, L21-L24), Gibberellin (GA3, L29-L32) and Yeast extract (YE, L25-L28) at final concentrations of 100 μM and 0.1%, respectively. Untreated tissue-cultured stolons were used as the 0-h time point in each series of experiments (L6 and L7).

1.1.3. Regenerated plants from stolons

G. uralensis plants were regenerated from tissue-cultured stolons of *G. uralensis* (Hokkaido-iryodai strain) following method as described in Kojoma *et al*. (2010). Leaves (L33) and stems (L34) were harvested one month after acclimatization. Lateral roots (L35) and main roots (L36 and L37) were harvested three month after acclimatization.

1.2 RNA extraction

The detailed of each sample numbers and sampling time for each stress treatments is described in Table S2. Total RNA was extracted from frozen plant tissues (0.1–0.5 g of tissue-cultured stolons treated with drought, plant hormones, or yeast extract elicitor). RNA was extracted using PureLink® Plant RNA Reagent (Thermo Fisher Scientific, Waltham, MA, USA) and treated with recombinant DNaseI (RNase-free) (Takara Bio, Shiga, Japan), then purified using the RNeasy® Plant Mini Kit (Qiagen, Hilden, Germany) following the RNA clean-up protocol.

1.3 Library construction, Illumina sequencing, and de novo assembly

A 10-μg aliquot of total RNA was used to construct a cDNA library using Agilent SureSelect Stand-Specific RNA Library Prep Kit, NEBNext Ultra RNA Library Prep Kit for Illumina or

Illumina TruSeq RNA Sample Prep Kit v2 (Illumina, San Diego, CA, USA) according to the manufacturer's protocol. The resulting cDNA library was sequenced using HiSeq 1500 or Miseq (Illumina). Total reads were assembled using Trinity ver 2.8.3 (Grabherr *et al*., 2011) after adaptor sequences and low-quality reads were removed by Trimmomatic ver. 0.38 (Bolger *et al*., 2014). A *de novo* assembly was performed by Trinity using 4 samples (L1-L4) from Ramilowski *et al*. (2013), 1 sample (L5) from Tamura *et al*. (2017), and 32 samples (L6-L37) from this study, obtaining a total of 37 total leads (Table S4). The expression level (TPM) of each contig in each read was obtained using the Trinity program. We submitted the raw RNA-Seq reads obtained in this study to the DNA Data Bank of Japan (DDBJ) Sequence Read Archive (DRA) under the accession number of DRA012266.

2. PSIII strain construction

The vector pESC-*Trp*-p*gal10*-t*HMG1*-*T2A*-*upc2-1* (Srisawat et al., 2020) was digested using *Xho*I/*Sal*I (New England Biolabs). The sequence of *Lotus japonicus* β-amyrin synthase was amplified from pYES3-ADH-OSC1 (Seki et al., 2008) using the 774/775 primer pair. The DNA fragments were then purified and constructed into the *Xho*I/*Sal*I sites of pESC-*Trp*-p*gal10*-t*HMG1*-*T2A*-*upc2-1* using In-Fusion Cloning Kit (Clontech). Then, the resulting plasmid, pESC-*Trp*-p*gal10*-t*HMG1*-*T2A*-*upc2-1*-p*gal1*-Lj*OSC1*, was transformed into yeast PSI (Srisawat et al., 2020). The resulting strain was named as PSIII.

774	TATAGGGCCCGGGCGATGTGGAAGCTGAAGGTAGCA
775	ACCAAGCTTACTCGATTAAACTGCAGTGGAAGGCAA

PSIII PSI/ pESC-*Trp*-p*gal10*-t*HMG1*-*T2A*-*upc2-1*-p*gal1*-Lj*OSC1*

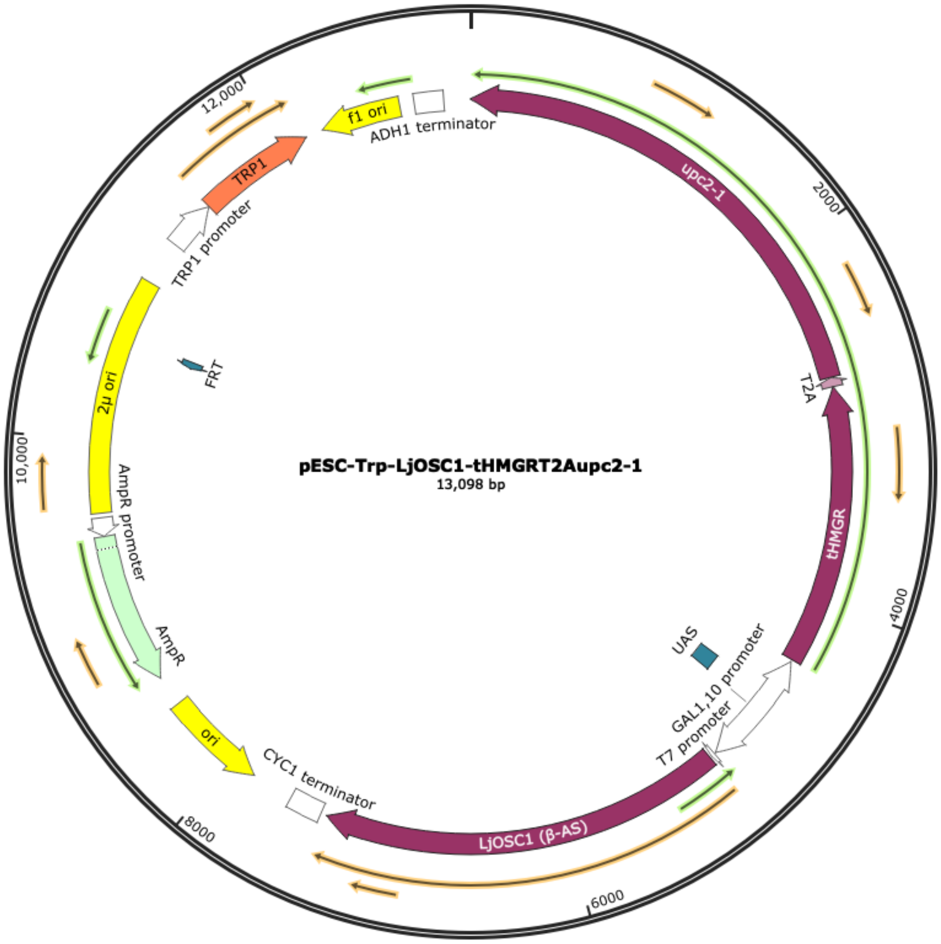

Table S1. List of accession numbers of CPR genes and amino acid sequences used for phylogenetic analysis and multiple sequence alignment in this study. Yellow color shows putative genes mined in this study.

Gene	Gene accession number	Protein accession number	Source
Arabidopsis thaliana CPR1 (ATR1)	NM_118585.4	NP_194183.1	NCBI
Arabodipsis thaliana CPR2 (ATR2)	NM_119167.4	NP_194750.1	NCBI
Medicago truncatula CPR1	XM_003602850.3	XP_003602898.1	NCBI
Medicago truncatula CPR2	XM_003610061.4	XP_003610109.1	NCBI
Lotus japonicus CPR1	Lj1g3v1548790.1	-	lotus.au.dk
Lotus japonicus CPR2	AB433810.1	BAG68945.1	NCBI
Glycyrrhiza uralensis CPR1	KY798117.1	AUG98241.1	NCBI
Glycyrrhiza uralensis CPR2	MH401048.1	QCZ35624.1	NCBI
Cicer arietinum CPR1	XM_004501597.3	XP_004501654.1	NCBI
Cicer arietinum CPR2	XM_004507801.3	XP_004507858.1	NCBI
Chenopodium quinoa CPR1	XM_021904070.1	XP_021759762.1	NCBI
Chenopodium quinoa CPR2	XM_021867713.1	XP_021723405.1	NCBI
Spinacia oleracea CPR1	XM_021999727.1	XP_021855419.1	NCBI
Spinacia oleracea CPR2	XM_022003966.1	XP_021859658.1	NCBI
Solanum lycopersicum CPR1	XM_004237953.3	XP_004238001.1	NCBI
Solanum lycopersicum CPR2	XM_004242883.4	XP_004242931.1	NCBI
Solanum tuberosum CPR1	XM_006337990.2	XP_006338052.1	NCBI
Solanum tuberosum CPR2	PGSC0003DMT400035801	-	www.plantgdb.org
Artemisia annua CPR1	PKPP01006895.1 (Whole genome sequence)	PWA55016.1	NCBI
Artemisia annua CPR2	EF104642.1	EF104642.1	NCBI
Catharantus roseus CPR2	X69791.1	CAA49446.1	
Oryza sativa CPR2a	CM000134.1	EAZ10065.1	NCBI
Oryza sativa CPR2b	AP008214.2	BAF23260.1	NCBI
Oryza sativa CPR2c	AL606690.3	CAE03554.2	NCBI
Triticum aestivum CPR2a	AJ303373.1	CAC83301.1	NCBI
Triticum aestivum CPR2c	AF123610.1	AAG17471.1	NCBI

Zea Mays CPR2b 1	EU955593.1	ACG27711.1	NCBI
Zea Mays CPR2b 2	EU956822.1	ACG28940.1	NCBI
Zea Mays CPR2c	BT061122.1	ACN25819.1	NCBI
Pseudotsuga menziesii CPR	CAA89837.3	Z49767.3	NCBI
Taxus chinensis CPR	AAX59902.1	AY959320.1	NCBI
Taxus cuspidata CPR	AAT76449.1	AY571340.1	NCBI
Physcomitrella patens CPR	EDQ49310.1	DS545408.1	NCBI
Selaginella moellendorffii CPR	XP_002978784.2	XM_002978738.2	NCBI
Chlamydomonas reinhardtii CPR	XP_042928682.1	XM_043058768.1	NCBI
Human CPR	NM_001395413.1	NP_001382342.1	NCBI

Table S2. Primer sequences for CPR genes

Gene	Primer Sequence	Target sequence
*MtCPR1*_for	CACCATGACTTCTTCCAATTCC GATTTAGTCCG	Amplification of *MtCPR1* for TOPO cloning
*MtCPR1*_rev	TCACCAGACATCCCTAAGGTAG CGTCCATCC	Amplification of *MtCPR1* for TOPO cloning
*MtCPR2*_for	CACCCCATGCAAGATTCAAGCT CAATG	Amplification of *MtCPR2* for TOPO cloning
*MtCPR2*_rev	GCCCGGTTCATCATTACCATAC ATCACG	Amplification of *MtCPR2* for TOPO cloning
*LjCPR1*_FOR	CACCATGACTTCGAATTCCGAT TTGGTTCG	Amplification of *LjCPR1* for TOPO cloning
*LjCPR1*_REV	TCACCAGACATCCCTGAGGTAA CGTCC	Amplification of *LjCPR1* for TOPO cloning
*LjCPR2*_for	CACCATGGAAGAATCAAGCTC CATGAAG	Amplification of *LjCPR2* for TOPO cloning
*LjCPR2*_rev	TCACCATACATCACGCAAATAC CTACC	Amplification of *LjCPR2* for TOPO cloning
*GuCPR1*_FOR	CACCATGACTTCGAATTCCGAT TTGGTTCG	Amplification of *GuCPR1* for TOPO cloning
*GuCPR1*_REV	TCACCAGACATCCCTGAGGTAA CGTCC	Amplification of *GuCPR1* for TOPO cloning
*GuCPR2*_FOR	CACCATGCAGGATTCAAACTCC ATGAAG	Amplification of *GuCPR2* for TOPO cloning
*GuCPR2*_REV	TCACCATACATCACGCAAATAC CTGCCA	Amplification of *GuCPR2* for TOPO cloning
*GuCPR1*_Inf_F w	GCCGCCCCCTTCACCATGACTT CGAATTCCGATT	Amplification of *GuCPR1* for HiFi-DNA Infusion TOPO cloning
*GuCPR1*_Inf_R v	GGCGCGCCCACCCTTTCACCAG ACATCCCTG	Amplification of *GuCPR1* for HiFi-DNA Infusion TOPO cloning
*GuCPR2*_Inf_F w	GCCGCCCCCTTCACCATGCAGG ATTCAAACTC	Amplification of *GuCPR2* for HiFi-DNA Infusion TOPO cloning
*GuCPR2*_Inf_R v	GGCGCGCCCACCCTTTCACCAT ACATCACGCA	Amplification of *GuCPR2* for HiFi-DNA Infusion TOPO cloning
*MtCPR1*_for1	ACTGACAATGCCGCAAGATT	Sequencing primer for *MtCPR1*
*MtCPR1*_for2	AGGGACCGGCGTAACATAC	Sequencing primer for *MtCPR1*
*MtCPR1*_for3	CACGTAACTTGTGCCCTGGT	Sequencing primer for *MtCPR1*
*MtCPR1*_rev1	ACCTAGGCCAAAAACACCAT	Sequencing primer for *MtCPR1*

*MtCPR1*_rev2	CCAACAACTTCCCAGCTTCT	Sequencing primer for *MtCPR1*
*MtCPR1*_rev3	GGGAATAGCATTCTTCATCCA	Sequencing primer for *MtCPR1*
*MtCPR2*_for1	TCTTAGCTACATATGGTGATGG TGA	Sequencing primer for *MtCPR2*
*MtCPR2*_for2	TCAGATCGTTCTTGCACTCA	Sequencing primer for *MtCPR2*
*MtCPR2*_for3	CATCATCTCCAAGAGTGGCA	Sequencing primer for *MtCPR2*
*MtCPR2*_rev1	CGAATCTTCTTCCCCTTCAA	Sequencing primer for *MtCPR2*
*MtCPR2*_rev2	CGGATAAATTCTCACAGTAAAC ACC	Sequencing primer for *MtCPR2*
*MtCPR2*_rev3	CACACTCCTTGATGAATCCG	Sequencing primer for *MtCPR2*
*MtCPR2*_rev4	TTGGAATTGTCCAAAGAGCC	Sequencing primer for *MtCPR2*
*LjCPR1*_FOR1	CCAACTGACAATGCTGCAAG	Sequencing primer for *LjCPR1*
*LjCPR1*_FOR2	TGATATATCGGGGACTGGCA	Sequencing primer for *LjCPR1*
*LjCPR1*_REV1	CCAACAACTTCCCAGCTTCT	Sequencing primer for *LjCPR1*
*LjCPR1*_REV3	GGGAATAGCATTCTTCATCCA	Sequencing primer for *LjCPR1*
LjCPR_for1	CACTGGCACTTTTCTTCTTAGC	Sequencing primer for *LjCPR2*
LjCPR_for2	TTCATACTCCTGTGTCAGATCG TT	Sequencing primer for *LjCPR2*
LjCPR_for3	GATTTTATTCGATCTCATCATC TCC	Sequencing primer for *LjCPR2*
LjCPR_rev1	CTCCCTCCAGAAACCATTTG	Sequencing primer for *LjCPR2*
LjCPR_rev2	TAAACACCAACATGGTCCCC	Sequencing primer for *LjCPR2*
LjCPR_rev3	ATGAATCCTACCAGTGGGCA	Sequencing primer for *LjCPR2*
LjCPR_rev4	CTGCTCTTGCAAAATTGTGTG	Sequencing primer for *LjCPR2*
*GuCPR1*_FOR1	CCAACTGACAATGCTGCAAG	Sequencing primer for *GuCPR1*
*GuCPR1*_FOR2	TGATATATCGGGGACTGGCA	Sequencing primer for *GuCPR1*
*GuCPR1*_REV2	CCAACAACTTCCCAGCTTCT	Sequencing primer for *GuCPR1*
*GuCPR1*_REV3	GGGAATAGCATTCTTCATCCA	Sequencing primer for *GuCPR1*
*GuCPR2*_FOR1	GGAGACACTCGCACTTTTCTTT	Sequencing primer for *GuCPR2*
*GuCPR2*_FOR2	TCTGTGTCGGATCGTTCTTG	Sequencing primer for *GuCPR2*
*GuCPR2*_REV2	GAAAACACCAACATGGTCCC	Sequencing primer for *GuCPR2*
*GuCPR2*_REV3	ATGAATCCTACCAGTGGGCA	Sequencing primer for *GuCPR2*
M1N_M2C_FO R	GGACTTCTCGTTTTTCTATGGC GTAGATCCAATTCTCAAAAACC	Amplification of fragments for N-terminal switching of MtCPRs

M1N_M2C_ backbone_REV	ATTGGATCTACGCCATAGAAAAACGAGAAGTCCAATTATGACGG	Amplification of fragments for N-terminal switching of MtCPRs
M2N_M1C_FOR	CGTCGTCGTTTTAATTTGGAAGAAATCTTCGGATCGGAGC	Amplification of fragments for N-terminal switching of MtCPRs
M2N_M1C_ backbone_REV	CCGAAGATTTCTTCCAAATTAAAACGACGACGCAACCG	Amplification of fragments for N-terminal switching of MtCPRs

Table S3. Similarity matrix of amino acid sequences of different CPR classes from different plant families.

CPR comparison		Average Similarity (%)	Max (%)	Min (%)
Fabales CPR-I	Fabales CPR-I	84	93	73
Fabales CPR-II	Fabales CPR-II	84	91	79
Fabales CPR-I	Fabales CPR-II	63	66	56
Amaranthaceae CPR-I	Amaranthaceae CPR-I	92	93	91
Amaranthaceae CPR-II	Amaranthaceae CPR-II	92	93	91
Amaranthaceae CPR-I	Amaranthaceae CPR-II	65	66	64
Fabales CPR-I	Amaranthaceae CPR-I	76	79	66
Fabales CPR-II	Amaranthaceae CPR-II	74	76	73
Fabales CPR-I/2	Amaranthaceae CPR-II/1	65	67	57
Fabales CPR-I	Other species CPR-I	75	79	64
Fabales CPR-II	Other species CPR-II	71	75	38
Fabales CPR-I/II	Other species CPR-II/I	62	66	51

Table S4.1. Co-expression analysis of CPR class I and II of *M. truncatula*

Medicago truncatula Gene Expression Atlas
(https://ljgea.noble.org/v2)

**PROBESET ID**	Mtr.10548.1.S1_at	Mtr.16806.1.S1_at	Mtr.43018.1.S1_at	Mtr.46721.1.S1_x_at	Mtr.8618.1.S1_at

No	Sample	Expression Level				
		MtCPR1	MtCPR2	CYP716A12	CYP72A63	CYP93E2
1	A17 LimN	549.865	2505.06	10063.3	367.644	5802.61
2	A17 rhizobia LimN LimN2	535.463	3164.05	13176.8	701.735	6040.38
3	A17 rhizobia SuffN	555.716	3595.01	5322.18	505.529	3616.44
4	A17 SuffN	572.883	2891.24	5815.79	332.803	3079.9
5	CS MJ 24h	1009.79	2066.66	3986.6	1154.52	5089.56
6	CS MJ 24h control	866.262	1251.75	94.6437	1033.19	6.94984
7	CS MJ 2h	719.5	983.063	59.4497	533.175	8.46596
8	CS MJ 2h control	725.08	952.834	79.2117	750.164	7.97788
9	CS YE 0h control	748.491	842.218	64.8876	890.197	7.77461
10	CS YE 24h	830.847	1218.48	98.5555	636.254	8.08189
11	CS YE 24h control	829.282	988.337	80.5682	630.807	7.25895
12	CS YE 2h	950.045	3491.71	96.5549	1314.4	7.72229
13	CS YE 2h control	699.142	872.37	84.7429	852.067	8.23441
14	Flower	710.282	1780.19	1877.73	1264.43	226.703
15	GallTissue-surrounding GiantCell (-)	289.866	246.937	410.711	180.305	1349.53
16	GiantCell	162.313	198.742	351.959	56.8172	1070.89
17	HairyRoot DMI3 Myc control	1034.68	1682.66	9774.39	160.724	5802.69
18	HairyRoot DMI3 Myc Infected	1018.94	1891.8	13790.9	144.053	5259.83
19	HairyRoot WT Myc control	1030.98	1319.42	3975.1	172.744	4785.81
20	HairyRoot WT Myc Infected	1021.84	1735.94	8100.62	179.263	5362.39
21	Hypocotyl A17 10C 100C-day1	787.485	2752.8	431.894	205.264	3018.35
22	Hypocotyl A17 10C 100C-day2	1090.29	4867.97	25.7109	1285.78	122.902
23	Hypocotyl A17 10C 35C-day1	1504.78	4900.35	24.9043	943.523	166.901
24	Hypocotyl A17 10C 35C-day2	627.309	2652.76	215.825	201.13	2999.97
25	Hypocotyl A17 10C 50C-day1	731.131	3035.31	118.845	418.48	1402.72
26	Hypocotyl A17 10C 50C-day2	690.975	3111.73	178.59	523.25	1210.83
27	Hypocotyl A17 20C 100C-day1	988.472	3442.69	71.2971	776.758	193.644
28	Hypocotyl A17 20C 100C-day2	467.699	3433.3	71.1957	857.508	336.323
29	Hypocotyl A17 20C 35C-day1	721.723	1967.08	831.971	172.004	2843.94
30	Hypocotyl A17 20C 35C-day2	727.636	1978.93	955.335	149.192	2369.05
31	Hypocotyl A17 20C 50C-day1	676.946	1968.41	472.759	247.25	1761.94
32	Hypocotyl A17 20C 50C-day2	859.1	2057.44	296.654	362.84	1744.52
33	Hypocotyl F83 10C 100C-day1	1397.43	5116.31	40.1844	156.156	93.4592
34	Hypocotyl F83 10C 100C-day2	1608.28	4807.43	34.9175	280.737	22.7766
35	Hypocotyl F83 10C 35C-day1	928.782	2615.94	1059.73	75.8633	3557.42
36	Hypocotyl F83 10C 35C-day2	1091.82	2745.83	1683.09	77.8814	3748.79
37	Hypocotyl F83 10C 50C-day1	1188.4	3096.49	138.098	111.107	1294.71

38	Hypocotyl F83 10C 50C-day2	740.699	2899.72	204.007	133.341	1761.39
39	Hypocotyl F83 20C 100C-day1	1114.23	3312.21	90.7535	84.1744	471.514
40	Hypocotyl F83 20C 100C-day2	1149.34	2912.19	90.1431	73.5206	630.392
41	Hypocotyl F83 20C 35C-day1	933.891	2191.83	1180.19	78.423	3880.37
42	Hypocotyl F83 20C 35C-day2	545.418	2295.78	1356.8	71.4978	4480.21
43	Hypocotyl F83 20C 50C-day1	835.534	2313.59	854.684	85.1256	2517.47
44	Hypocotyl F83 20C 50C-day2	626.545	2316.11	1214.11	79.3721	3507.17
45	Leaf GUS-ox	756.854	4077.76	1089.48	60.834	885.472
46	Leaf MtLAP1	899.155	4226.06	123.74	58.459	857.688
47	Leaf	789.463	2867.62	1234.81	95.4797	3162.99
48	Leaf 2HA 1wk	1092.28	1046.17	3483.47	237.207	2110.1
49	Leaf 2HA 1wk NAA	1110.51	1738.53	1118.23	704.331	831.409
50	Leaf 2HA 2wk NAA BAP	1115.55	967.996	9218.36	380.393	6125.45
51	Leaf CYO716A12	1140.43	2777.72	609.113	95.0358	1554.32
52	Leaf CYO716A12 R108	1043.6	2497.16	343.589	86.7302	3332.57
53	Leaf IRG1 R108	671.492	1201.86	1088.32	83.2136	2401.88
54	Leaf irg1-1	667.236	1533.54	122.441	125.774	2296.54
55	Leaf irg1-2	698.757	1425.11	78.9439	123.791	2567.28
56	Leaf irg1-5	709.333	1394.31	167.602	154.318	2231.79
57	Leaf Jemalong 2wk NAA BAP	1074.86	1354.18	3233.17	507.537	1965.46
58	Nod 10dpi	938.304	2032.22	3821.73	155.573	1510.65
59	Nod 14dpi	799.052	1870.53	8079.94	171.09	3791.42
60	Nod 16dpi+NO3	1030.24	2362.16	4103.54	313.773	1765.64
61	Nod 28dpi	804.081	1802.52	7534.37	440.136	4398.01
62	Nod 4dpi	836.382	2815.74	12467.2	113.125	5921.4
63	Nod Naut1 SalsB	298.174	2635.99	22.4752	19.452	69.1896
64	Nod Naut1 SalsC	163.04	2559.33	14.208	18.3153	32.7627
65	Nod phosphinothricin 0h	762.24	1314.04	9760.65	632.027	5655.08
66	Nod phosphinothricin 24h	726.243	2064.33	1586.93	633.353	2627.35
67	Nod phosphinothricin 4h	736.43	2106.1	7281.3	1120.05	3953.18
68	Nod phosphinothricin 8h	901.318	3015.45	1805.4	1213.81	2964.55
69	Nod R108	1210.07	1660.16	6631.25	35.3051	4431.05
70	Nod Sals4 SalsB	345.491	3119.45	15.2906	101.853	10.2792
71	Nod Sals4 SalsC	294.59	2725.96	21.3576	144.27	13.099
72	Nod zone2	308.056	205.695	102.441	170.901	737.104
73	Nod NOOT NF2717	1222.69	1734.96	4971.41	43.9582	5813.72
74	Nod NOOT TNK507	1215.28	1957.15	5042.44	40.8221	6231.88
75	Petiole	804.517	2266.21	1672.1	139.869	3246.22
76	Pod	647.49	1232.28	4936.35	243.736	17.6873
77	Root	898.32	3195.64	2859.45	459.78	3798.12
78	Root 0dpi	984.247	2930.64	11070.2	510.171	8113.11
79	Root 2d Sdl culture 180mM NaCl 0h	904.974	2492.66	8880.84	160.837	1899.64
80	Root 2d Sdl culture 180mM NaCl 6h	1087.1	4334.12	1112.43	2161.71	3358.42

81	Root 2d Sdl culture 180mM NaCl 24h	1106.99	4450.87	1360.21	1865.58	1413.56
82	Root 2d Sdl culture 180mM NaCl 48h	1151.58	3809.86	162.43	1665.97	447.698
83	Root 1cm adj tip	1030.27	1768.11	1409.97	472.85	6262.49
84	Root 2wk Sdl Hydroponic 200mM NaCl 0h	1000.74	4340.77	3211.65	718.24	3698.52
85	Root 2wk Sdl Hydroponic 200mM NaCl 10h	1171	6199.04	1250.73	1963.56	4273.83
86	Root 2wk Sdl Hydroponic 200mM NaCl 1h	783.385	4331.2	2970.45	1006.28	3773.41
87	Root 2wk Sdl Hydroponic 200mM NaCl 24h	1092.85	6606.46	858.029	1422	895.707
88	Root 2wk Sdl Hydroponic 200mM NaCl 2h	1065.86	5897.92	1267.35	2255.94	6161.74
89	Root 2wk Sdl Hydroponic 200mM NaCl 5h	1190.35	5982.04	2187.29	2854.63	7407.97
90	Root 3mm tip	1158.92	1807.77	9004.97	302.111	2021.46
91	Root 5wk LimtNH4 NH4C	953.909	3070.97	5570.44	276.092	4740.45
92	Root 5wk LimtNO3 NO3C	882.043	2454.52	6039.19	259.987	4838.48
93	Root 5wk NoNO3 NO3ONE	856.201	2949.66	7385.15	260.368	4076.27
94	Root 5wk SuffNH4 NH4S	1081.83	2884.43	4631.12	268.594	3082.04
95	Root 5wk SuffNO3 NO3S	993.98	3102.93	4458.51	277.425	3546.97
96	Root A17 1dpi (Aphanomyes)	1035.4	3145.05	2134.01	297.991	1159.85
97	Root A17 buffer 1d	1155.06	3728.46	2678.01	319.284	6163.05
98	Root A17 buffer 7d	988.26	3671.23	2276.39	390.644	3781.92
99	Root A17 mock	937.925	1462.44	3561.94	302.567	3444.98
100	Root A17 NPA 1d	1136.39	2821.07	200.819	347.929	3801.7
101	Root A17 NPA 7d	974.032	4648.07	1482.79	273.596	3796.05
102	Root A17 TIBA 1d	989.011	3098.84	1233.14	1004.7	4247.27
103	Root A17 TIBA 7d	903.008	3401.72	2861.33	499.601	5584.22
104	Root Border cell	1016.77	2470.71	6995.28	267.429	6857.72
105	Root CRR 0hpi	1079.04	2882.82	1157.79	134.896	4287.37
106	Root CRR 72hpi	945.351	3633.34	963.475	296.298	3370.11
107	Root CRR 96hpi	772.606	4724.36	290.329	532.277	110.598
108	Root (denodulated) 28dpi	814.09	4011.3	8267.51	426.451	6229.26
109	Root DMI3 MF control 6h	1272.36	2391.14	425.039	398.398	1773.43
110	Root DMI3 nsMyc-LCOs 6h	1257.17	2267.46	622.397	410.186	2342.18
111	Root DMI3 sMyc-LCOs 6h	1235.17	2278.91	505.137	475.909	1791.63
112	Root DMI3 s-nsMyc-LCOs 6h	1269.81	2456.06	236.079	470.148	931.851
113	Root drought 10d	945.042	2641.74	6758.72	377.28	9430.08
114	Root drought 14d	1223.36	2481.45	3734.69	320.941	8717.32
115	Root drought 14d rewatered 1d	988.962	3074.89	4468.09	633.501	4796.31
116	Root drought 2d	860.389	2878.79	6476.74	292.883	7192.21
117	Root drought 3d	827.122	2961.5	4711.92	376.44	7631.13
118	Root drought 4d	843.641	2954.01	2611.9	366.623	7644.58
119	Root drought 7d	926.043	2671.59	6103.66	267.409	9113.63
120	Root J5 10mM KNO3	568.69	2023.54	6712.28	849.008	2526.51
121	Root J5 1mM KNO3	753.819	2136.76	6069.62	1174.01	1890.08

122	Root LCM adjacent	629.695	1034.94	2573.03	234.687	3357.1
123	Root LCM arbuscular	138.453	773.941	1994.78	425.695	2465.67
124	Root LCM cortical	574.187	313.603	3177.48	59.0581	3026.49
125	Root Macrophomina infected 0hpi	787.475	1524.38	2093.97	373.842	4751.7
126	Root Macrophomina infected 24hpi	839.174	1776.85	2198.72	435.194	4198.1
127	Root Macrophomina infected 36hpi	754.843	1974.9	2002.34	446.87	2555.22
128	Root Macrophomina infected 48hpi	725.363	2016.04	1979.97	433.495	2880.53
129	Root Myc control	928.551	4187.59	8589.3	237.809	5066.75
130	Root Myc infection	919.876	4065.06	9676.48	280.6	5392.57
131	Root Myc 3wk infection	434.617	319.947	500.073	218.75	1954.81
132	Root NFP MF control 6h	1267.74	2423.61	562.44	431.284	2634.75
133	Root NFP nsMyc-LCOs 6h	1184.86	2167.77	680.176	360.587	2942.25
134	Root NFP sMyc-LCOs 6h	1240.92	2275.94	836.226	383.718	3963.05
135	Root NFP s-nsMyc-LCOs 6h	1278.78	2413.48	373.779	362.252	2083.8
136	Root nfp-2 1dpi (Aphanomyes)	977.432	2964.65	1746.35	224.075	413.662
137	Root nfp-2 mock	936.801	1550.11	3878.89	274.829	4735.25
138	Root Nod 5wk LimtN2 N2C	720.57	3645.14	6530.96	393.534	3596.98
139	Root Nod 5wk SuffN2 N2S	943.589	5187.08	2006.56	572.849	1410.62
140	Root R108	1374	3352.48	3429.29	81.9637	6083.54
141	Root tip	1230.61	2318.94	14216.1	275.115	943.091
142	Root Tip A17 0hpi (Ralstonia)	555.193	1827.32	10160	247.655	3746.29
143	Root Tip A17 12hpi (Ralstonia)	686.751	2184.03	13336.7	448.743	4287.51
144	Root Tip A17 72hpi (Ralstonia)	615.297	2649.29	5120.52	893.731	7146.71
145	Root Tip F83005.5 0hpi (Ralstonia)	891.184	1751.08	4107.42	73.0557	6098.42
146	Root Tip F83005.5 12hpi (Ralstonia)	1138.46	2371.14	4487.16	83.985	5703.59
147	Root Tip F83005.5 72hpi (Ralstonia)	1031.17	3453.57	1323.79	49.65	10947.8
148	Root Tip sickle 0hpi (Ralstonia)	633.9	2274.49	10757.5	274.638	3955.42
149	Root Tip sickle 12hpi (Ralstonia)	883.626	2942.73	15765.3	438.291	2627.73
150	Root Tip sickle 72hpi (Ralstonia)	727.561	2728.29	8100.7	304.546	5685.83
151	Root tr185 10mM KNO3	773.113	2521.02	7605.12	546.326	1200.31
152	Root tr185 1mM KNO3	537.095	2354.99	6642.2	781.468	1508.32
153	Root transgenic GUS (TT2 control)	786.677	3760.53	6896.94	803.413	3442.43
154	Root transgenic TT2	786.4	4270.25	8015.76	887.026	2594.18
155	Root watered 4d	1040.11	3021.46	3674.13	332.945	4438.52
156	Root watered d2	940.482	2758.01	3652.87	246.138	3927.11
157	Root Whole	942.498	3382.42	8874.25	256.599	9373.18
158	Root WT MF control 24h	1061.52	2108.12	2350.9	237.031	5649.13
159	Root WT MF control 6h	1168.51	2112.32	625.292	371.626	3278.08
160	Root WT NF control 24h	1079.08	2132.42	2733.7	293.94	5167.17
161	Root WT NF control 6h	1191.5	2272.11	714.431	475.604	2429.91
162	Root WT Nod-LCOs 24h	1076.99	2116.83	2444.22	268.722	5038.31
163	Root WT Nod-LCOs 6h	1202.56	2397.59	639.813	432.278	1658.56
164	Root WT nsMyc-LCOs 24h	983.224	2019.17	2175.43	220.323	6264.77

165	Root WT nsMyc-LCOs 6h	1104.98	2005.21	1057.92	382.782	2978.24
166	Root WT sMyc-LCOs 24h	1019.53	2123.93	2256.73	219.79	5856.16
167	Root WT sMyc-LCOs 6h	1160.53	2108	715.615	351.718	3428.93
168	Root WT s-nsMyc-LCOs 24h	1056.53	2068.54	1989.15	220.425	4109.85
169	Root WT s-nsMyc-LCOs 6h	1123.97	2100.32	923.655	369.98	3001.44
170	Seed 10dap	980.069	895.13	12.9588	271.766	6.76733
171	Seed 12dap	876.553	898.807	10.6306	161.428	11.7309
172	Seed 16dap	662.388	969.184	17.1374	136.623	567.176
173	Seed 20dap	856.335	1355.62	4896.32	369.12	3597.73
174	Seed 24dap	1044.28	1534.47	13829.2	569.655	2248.1
175	Seed 36dap	1532.39	633.173	4875.77	411.721	2367.67
176	Seed Coat	880.599	1378.56	372.884	787.172	300.654
177	Seeds transgenic R108 (control)	757.069	1051.71	2297.29	113.525	2366.92
178	Seeds transgenic R108 GuarMannSynt	976.942	924.078	4137.84	77.6112	2854.38
179	Shoot 5wk LimtN2 N2PAE	1013.01	3051.73	2255.7	397.388	4266.85
180	Shoot 5wk LimtNH4 NH4PAE	580.445	1327.94	1782.1	297.96	4658.96
181	Shoot 5wk LimtNO3 NO3PAE	717.989	1408.78	1500.56	252.557	4344.99
182	Shoot 5wk SuffN2 N2PAT	917.495	3237.05	1709.34	406.736	2587.52
183	Shoot 5wk SuffNH4 NH4PAT	515.019	1117.26	1338.65	204.941	4110.01
184	Shoot 5wk SuffNO3 NO3PAT	662.877	1275.8	1310.2	204.03	4352.79
185	Shoot drought 10d	937.352	3548.34	1258.05	363.845	6956.82
186	Shoot drought 14d	1024.12	3352.57	1111.58	324.342	6815.4
187	Shoot drought 14d rewatered 1d	618.904	1849.72	2870.43	91.3732	4188.23
188	Shoot drought 2d	771.93	2815.67	2313.27	108.892	3707.99
189	Shoot drought 3d	750.528	2682.86	1634.27	129.496	4328.01
190	Shoot drought 4d	883.125	3451.89	222.621	251.915	5163.38
191	Shoot drought 7d	872.567	2937.32	1191.56	344.762	7226.46
192	Shoot R108	613.69	1149.37	7617.96	129.319	2981.23
193	Shoot stenofolia	681.535	1268.13	7392.06	212.814	3108.05
194	Shoot watered 4d	776.811	2740.04	3307.95	113.743	3834.91
195	Shoot watered 2d	710.72	2439.1	2469.85	95.7753	3164.53
196	Stem	725.349	2007.94	1292.47	157.906	3700.12
197	Stem internode1	870.856	1411.27	3753.97	70.8201	5289.68
198	Stem internode2	792.933	1554.98	430.382	138.803	3215.23
199	Stem internode4	693.796	3893.03	13.8758	451.88	2424.39
200	Stem internode6	693.922	4153.42	12.6888	295.011	4793.87
201	Stem internode8	762.402	4217.32	22.5654	320.147	6566.87
202	Stem stp	912.938	4507.23	17.1907	141.055	3945.34
203	Stem WT-STP R108	907.962	4026.92	45.9049	97.82	4777.33
204	Stem nStem1-2	798.275	2946.65	41.9918	68.3813	5432.71
205	Stem nStem1-p	762.099	3269.38	54.3105	41.9828	4764.28
206	Stem R108-NStem1-2	810.932	3225.97	24.2969	271.561	4819.12
207	Stem R108-NStem1-P	890.284	3539.24	48.2339	128.754	4930.79

208	sunn LimN	732.663	2549.41	8907.82	763.229	2366.7
209	sunn SuffN	695.269	2377.68	7340.17	618.278	2971.87
210	Vegetative Buds	677.251	1907.57	4379.54	57.8472	4523.7
211	Nod 3dpi	846.03	2690.41	11235.6	249.358	8378.21
212	Nod 6dpi	692.469	1295.14	12205.6	104.944	4409.18
213	Nod 10dpi C	774.363	1809.96	7788.05	335.842	3431.79
214	Nod 14dpi C	862.839	1691.06	7976.52	148.24	5020.14
215	Nod 20dpi	868.638	1147.71	10209.5	960.479	5175.33
216	Root 3dpi (control)	1027.96	1717.84	8001.3	291.361	3955.6
217	Root 6dpi (control)	1055.62	1774.47	8039.88	282.107	3057.91
218	Root 10dpi (control)	970.852	1991.43	5175.34	214.653	3313.73
219	Root 14dpi (control)	881.424	1786.4	6594.21	358.283	1987.13
220	Root 20dpi (control)	849.509	1596.62	8503.46	274.999	5527.26
221	Root meristem	908.165	1521.21	10031.1	199.498	1006.88
222	Root (28dpi) Myc (G. intraradices) 6wk 20 uM P	778.337	2005.15	5944.5	334.11	6188.06
223	Root (28dpi) Myc (G. mosseae) 6wk 20 uM P	726.542	2418.46	7858.3	460.933	4332.01
224	Root non-Myc (control) 6wk 20 uM P	937.579	2822.59	7494.4	774.212	6586.56
225	Root non-Myc 6wk 2 mM P	815.479	1988.39	5116.79	598.694	7171.74
226	Shoot 10wk (41dpi)	1168.55	2159.11	3713.5	255.624	5393.1
227	Flower 12wk (56dpi)	856.657	777.836	2056.13	1023.59	1125.42
228	Pod 12wk (56dpi)	1103.13	1065.15	443.799	1182.96	35.374
229	Nodule (20d)	762.24	1314.04	9760.65	632.027	5655.08
230	Nodule (20d) - 4h PPT	736.43	2106.1	7281.3	1120.05	3953.18
231	Nodule (20d) - 8h PPT	901.318	3015.45	1805.4	1213.81	2964.55
232	Nodule (20d) - 24h PPT	726.243	2064.33	1586.93	633.353	2627.35
233	Root sunn-2 skl 5d seedling - 4h Nod factor	1006.08	2479.47	13637.6	507.073	5749.88
234	Root sunn-2 skl 5d seedling - 24h Nod factor	1028.78	1868.81	9747.65	296.808	4508.03
235	Root sunn-2 skl 5d seedling - 4h Mock	958.505	2704.15	13206	535.819	6919.42
236	Root sunn-2 skl 5d seedling - 24h Mock	1017.61	2279.23	11918.2	394.509	6623.48
237	Root A17 5d seedling - 24h Nod factor	954.779	2527.86	9617.93	354.387	5781.23
238	Root A17 5d seedling - 24h Mock	927.313	2514.66	9508.1	358.738	6231.48
239	Root YA1/HAP2 5d seedling - 24h Nod factor	954.504	2406.16	9174.32	338.745	5355.15
240	Root ya1/hap2 5d seedling - 24h Mock	949.022	2465.81	9188.05	377.721	6014.77
241	Root ya1/hap2 5d seedling - (T0) non-inoculated (N-starved)	1136.59	2346.37	11000.9	416.899	8731.68
242	Root efd 5d seedling - N-starved	1177.39	2296.44	11139	400.622	8733.77
243	Root A17 5d seedling - N-starved	1097.99	2353.08	11995.7	403.898	9192.53
244	Nodule A17 (6dpi)	893.677	1945.48	10552.2	278.489	6385.47
245	Nodule ya1/hap2 (6dpi)	841.58	2547.38	13849.9	169.45	7940.85
246	Nodule efd (6dpi)	816.768	2624.07	12665.3	242.305	8891
247	Root A17 5d seedling - 1dpi Sinorhizobium	1115.93	2212.92	13149.4	381.038	8942.06
248	Root ya1/hap2 5d seedling - 1dpi Sinorhizobium	1153.92	2250.77	13055.3	411.299	9595.8

249	Root A17 5d seedling - 3dpi Sinorhizobium	1113.65	2326.66	14054.5	377.519	10098.5
250	Root ya1/hap2 5d seedling - 3dpi Sinorhizobium	1093.23	2473.84	14463.7	423.509	9790.55
251	Root A17 control	1030.98	1319.42	3975.1	172.744	4785.81
252	Root DMI3 control	1034.68	1682.66	9774.39	160.724	5802.69
253	Root A17 inoculated with Gigaspora (early contact)	1021.84	1735.94	8100.62	179.263	5362.39
254	Root DMI3 inoculated with Gigaspora (early contact)	1018.94	1891.8	13790.9	144.053	5259.83
255	root hairs 1 dpi Sm1021 nodD1ABC deletion mutant	996.277	2529.55	2477.55	449.119	4505.67
256	root hairs 3 dpi Sm1021 nodD1ABC deletion mutant	1020.73	2214.71	1756.05	491.749	4804.42
257	root hairs 5 dpi Sm1021 nodD1ABC deletion mutant	1045.19	2196.62	324.683	604.667	4830.12
258	root hairs 1 dpi Sm1021	1067.74	2444.72	2004.64	489.971	3829.87
259	root hairs 3 dpi Sm1021	1113.09	1603.5	1641.4	429.489	3562.4
260	root hairs 5 dpi Sm1021	1040.23	1794.27	294.524	473.723	3892.16
261	root hairs 5 dpi Sm1021 nodD1ABC deletion mutant sickle-1	895.525	1669.82	102.846	186.754	6092.15
262	root hairs 5 dpi Sm1021 sickle	735.149	981.809	707.946	189.644	1871.37
263	root hairs 24h Nod factor control	1161.27	2390.09	1777.87	691.987	4005.86
264	root hairs 24h 10 nM Nod factors	965.016	2108.35	1282.84	376.947	1953.79
265	Epidermal cells from root areas containing mature mycorrhizal structures	14.93	9.06	4.29	17.88	1332.57
266	Cortical cells from mycorrhizal roots containing fungal hyphae	7.06	8.57	284.05	3.43	2418.67
267	Cortical cells from mycorrhizal roots containing arbuscules	11.79	11.55	26.72	3.46	427.57
268	Epidermal and cortical cells from non-colonized areas of mycorrhizal roots	30.27	34.06	230.72	10.63	2628.46
269	Uninfected cells from Medicago truncatula root nodules	294.07	3.32	1296.13	15.78	77.17
270	Infected cells from Medicago truncatula root nodules	210.84	4.17	172.45	25.63	73.52
271	Epidermal and adjacent cortical cells from areas with visible appressoria	48.17	35.02	64.89	9.38	2876.3
272	Proximal infection zone cells from Medicago truncatula root nodules	424.61	4.47	709.18	13.18	32.9
273	Meristem cells from Medicago truncatula root nodules	481.04	3.48	1067.48	9.25	40.5
274	Distal infection zone cells from Medicago truncatula root nodules	427.57	4.44	754.83	9.65	41.64

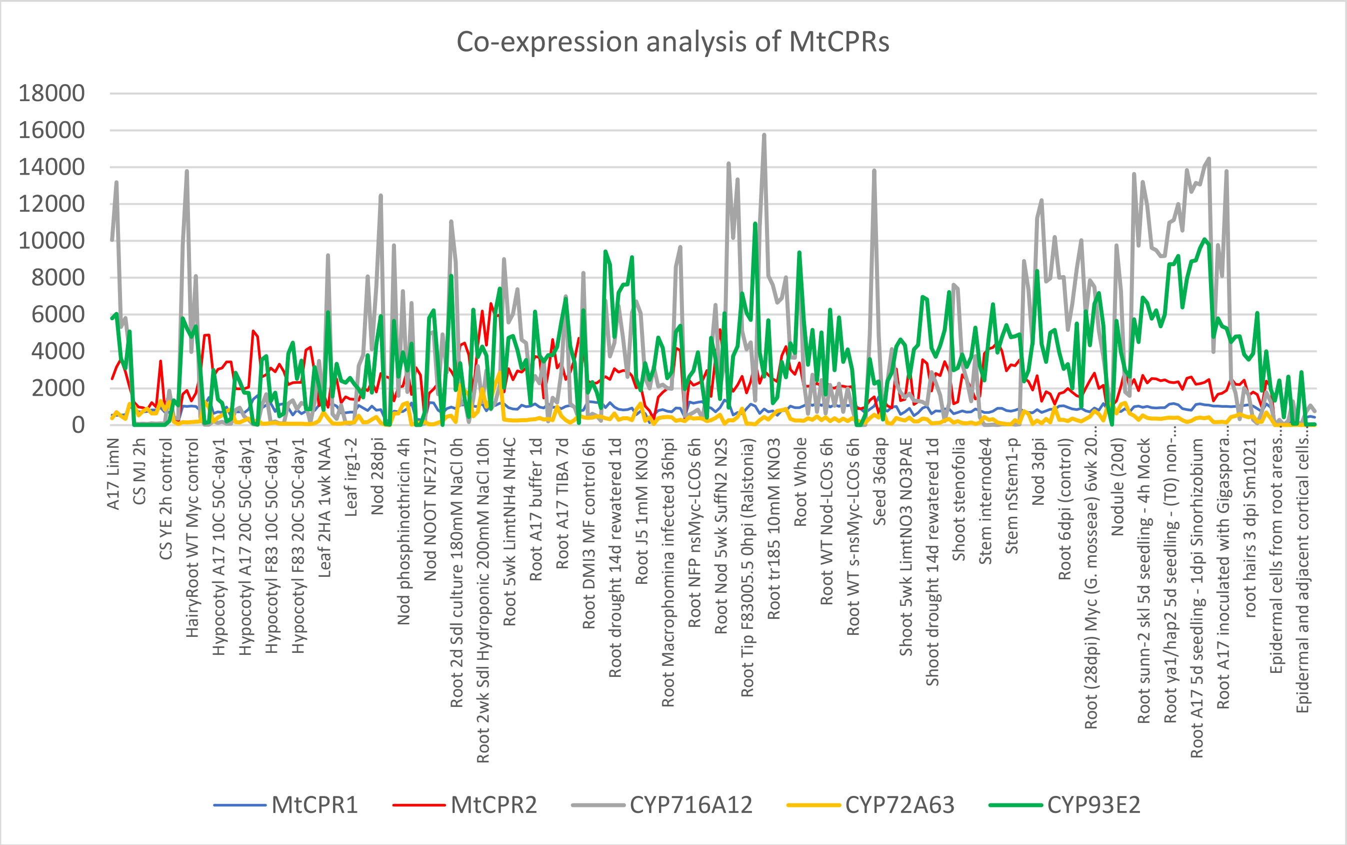

Pearson Correlation Coefficient Table

	MtCPR1	MtCPR2	CYP716A12	CYP72A63	CYP93E2
**MtCPR1**	1.00	0.41	0.12	0.22	0.25
**MtCPR2**	0.41	1.00	-0.03	0.41	0.17
**CYP716A12**	0.12	-0.03	1.00	-0.02	0.53
**CYP72A63**	0.22	0.41	-0.02	1.00	-0.06
**CYP93E2**	0.25	0.17	0.53	-0.06	1.00

Table S4.2. Co-expression analysis of CPR class I and II of *L. japonicus*

https://lotus.au.dk/expat			Miyakojima MG20 v 3.0		
**GENE ID**	Lj1g3v1548790	Lj0g3v0139899			
**PROBESET ID**	Ljwgs_006504.2_at	Ljwgs_068084.1_at	Ljwgs_061908.1_at	chr3.TM0797.5_at	Ljwgs_008809.1_at

No	Sample	Expression Level				
		LjCPR1	LjCPR2	CYP716A51	CYP72A61	CYP93E1
1	WT_control1	669.54	2171.74	51.83	6972.63	6773.77
2	WT_Drought1	639.66	1731.79	28.71	5418.49	8667.44
3	Ljgln2_2_Control1	561.19	1915.56	39.63	8716.81	12206.9
4	Ljgln2_2_Drought1	686.9	1684.87	30.96	7851.82	10711.5
5	root_4dpicontrol1B	777.03	174.24	2436.09	6558.59	8934.18
6	root_28dpicontrol1A	778.5	211.78	4687.2	8407.51	11076.7
7	root_4dpimycorrhized1D	800.85	180.45	3752.38	3218.58	4183.06
8	root_28dpimycorrhized1C	712.65	175.88	2631.32	2696.27	3353.37
9	WT_root_tip_3w_uninocul_1	1250.82	1290.42	59.95	5681.32	6535.7
10	WT_root_3w_uninocul_1	990.96	1156.2	2207.88	8157.18	11526.5
11	WT_root_3w_5mM_nitrate_1	1013.1	888.23	638.41	1666.99	1042.05
12	WT_root_6w_5mM_nitrate_1	917.06	608.74	1234.99	1437.3	685.11
13	WT_shoot_3w_5mM_nitrate_1	691.39	2811.11	55.62	4949.89	6581.59
14	WT_shoot_3w_uninocul_1	629.65	2410.43	163.22	2904.18	3001.13
15	WT_shoot_3w_inocul3_1	735.69	2469.43	167.11	3341.18	4182.67
16	WT_leaf_6w_5mM_nitrate_1	789.19	1827.43	22.79	5212.29	7668.76
17	WT_stem_6w_5mM_nitrate_1	823.13	1228.23	515.24	8591.87	12138
18	har1_root_3w_uninocul_2	922.86	737	2682.78	4842.06	5417.32
19	har1_root_3w_inocul3_2	1027.61	609.97	1545.2	1291.39	1712.66
20	har1_shoot_3w_inocul3_1	728.44	3005.95	289.41	4502.84	4716.7
21	WT_root_3w_nodC_inocul1_1	922.47	1005.53	1901.01	1215.53	1498.83
22	WT_root_3w_inocul1_1	999.07	941.21	1542.77	2581.09	3999.42
23	WT_root_3w_inocul3_1	1037.97	1278.44	1902.25	2956.12	4241.22
24	WT_nodule_3w_inocul14_1	831.53	736.66	528.07	4480.81	2032.94
25	WT_nodule_3w_inocul21_1	814.8	681.86	478.7	1962.98	1901.95
26	WT_root_nodule_3w_inocul21_1	903.64	1087.86	1600.52	265.81	146.24
27	WT_rootSZ_3w_inocul1_1	894.07	2020.8	599.95	1205.35	844.32
28	WT_rootSZ_3w_Nod_inocul1_1	663.13	1182.15	1654.69	1543.77	1040.2
29	nfr5_rootSZ_3w_uninocul_1	829.3	3140.91	1183.31	2232.13	1393.07
30	nfr5_rootSZ_3w_inocul1_1	859.95	1939.23	406.54	2614.88	1310.9
31	nfr1_rootSZ_3w_uninocul_1	776.27	1138.66	823.64	2295.09	1668.98
32	nfr1_rootSZ_3w_inocul1_1	911.44	1530.67	467.41	2387.2	1021.17
33	nup133_rootSZ_3w_uninocul_1	942.86	1018.66	437.77	2700.35	1813.02

34	nup133_rootSZ_3w_inocul1_1	980.85	1595.51	430.03	1829.34	1350.89
35	nin_rootSZ_3w_uninocul_1	869.1	1910.19	1313.88	7803.27	9991.03
36	nin_rootSZ_3w_inocul1_1	881.11	2020.7	1037.73	2089.9	1541.07
37	sen1_root_3w_uninocul_1	1000.08	975.73	1489.4	1921.97	1297.72
38	sen1_nodule_3w_inocul21_1	808.9	1125.44	498.45	1179.51	519.48
39	sst1_root_3w_uninocul_1	1026.72	1054.78	1538.64	2547.28	1850.2
40	sst1_nodule_3w_inocul21_1	795.33	950.66	439.75	785.47	415.04
41	Shoot_0mM_sodiumChloride_1	644.72	1419.61	65.93	2326.9	1837.2
42	Shoot_25mM_sodiumChloride_Initial_1	603.54	1912.66	36.37	3191.95	2395.22
43	Shoot_50mM_sodiumChloride_Initial_1	699.68	2281.72	42.53	3769.22	2441.84
44	Shoot_75mM_sodiumChloride_Initial_1	683.85	2104.55	27.46	9434.24	11603.2
45	Shoot_50mM_sodiumChloride_Gradual_1	689.74	2044.1	29.11	10491.3	10819.2
46	Shoot_100mM_sodiumChloride_Gradual_1	637.55	1671.52	67.49	3641	4137.58
47	Shoot_150mM_sodiumChloride_Gradual_1	671.26	1941.62	51.59	4127.7	4745.98
48	Lburttii_Ctrol_A	982.65	2206.82	37.87	7394.33	11535
49	Lburttii_Salt_A	818.58	2496.55	29.88	5471.92	7596.59
50	Lcorniculatus_Ctrol_A	694.5	1735.92	38.03	8404.65	13160.2
51	Lcorniculatus_Salt_A	782	2129.92	34.18	2316.32	2489.77
52	Lfilicaulis_Ctrol_A	787.89	1405.03	113.89	2798.22	3153.32
53	Lfilicaulis_Salt_A	801.06	1663.64	54.41	5162.69	6517.18
54	Lglaber_Ctrol_A	852.04	2704.95	49.9	7056.56	9854.9
55	Lglaber_Salt_A	831.95	2737.46	39.85	6154.09	9601.44
56	Ljaponicus_Gifu_Ctrol_A	759.01	2282.72	30.81	5014.88	7771.07
57	Ljaponicus_Gifu_Salt_A	788.02	3341.68	25.6	5090.25	7629.26
58	Ljaponicus_MG20_Ctrol_A	650.8	1876.4	27.46	6582.08	11153.8
59	Ljaponicus_MG20_Salt_A	716.18	1993.23	25.89	6335.76	10653
60	Luliginosus_Ctrol_A	769.38	1241.57	38.39	5970.82	9355.83
61	Luliginosus_Salt_A	719.13	1302.98	29.01	5203.14	7442.12
62	Fl_1	769.42	2499.52	17.16	8355.16	9308.57
63	Pod20_1	702.99	575.44	32.21	6172.35	6301.8
64	Seed10d_1	800.58	1247.04	77.93	1810.25	1325.82
65	Seed12d_1	795.79	1238.01	65.6	2039.34	1626.93
66	Seed14d_1	747.1	1003.78	42.54	2491.14	2191.85
67	Seed16d_1	707.75	953.88	27.06	1533.29	856.13
68	Seed20d_1	635.65	387.96	23.31	4984.22	5508.79
69	Leaf_1	831.88	5161.46	17.53	10735.4	9804.87
70	Pt_1	867.93	2065.67	20.71	5871.47	8830.46

71	Stem_1	802.52	1932.78	30.7	7748.95	11105.2
72	Root_1	1079.27	1443.08	590.96	2822.5	2044.87
73	Root0h_1	1245.94	1637.02	448.61	2280.15	1402.27
74	Nod21_1	734.69	519.08	780.65	177.8	62.44

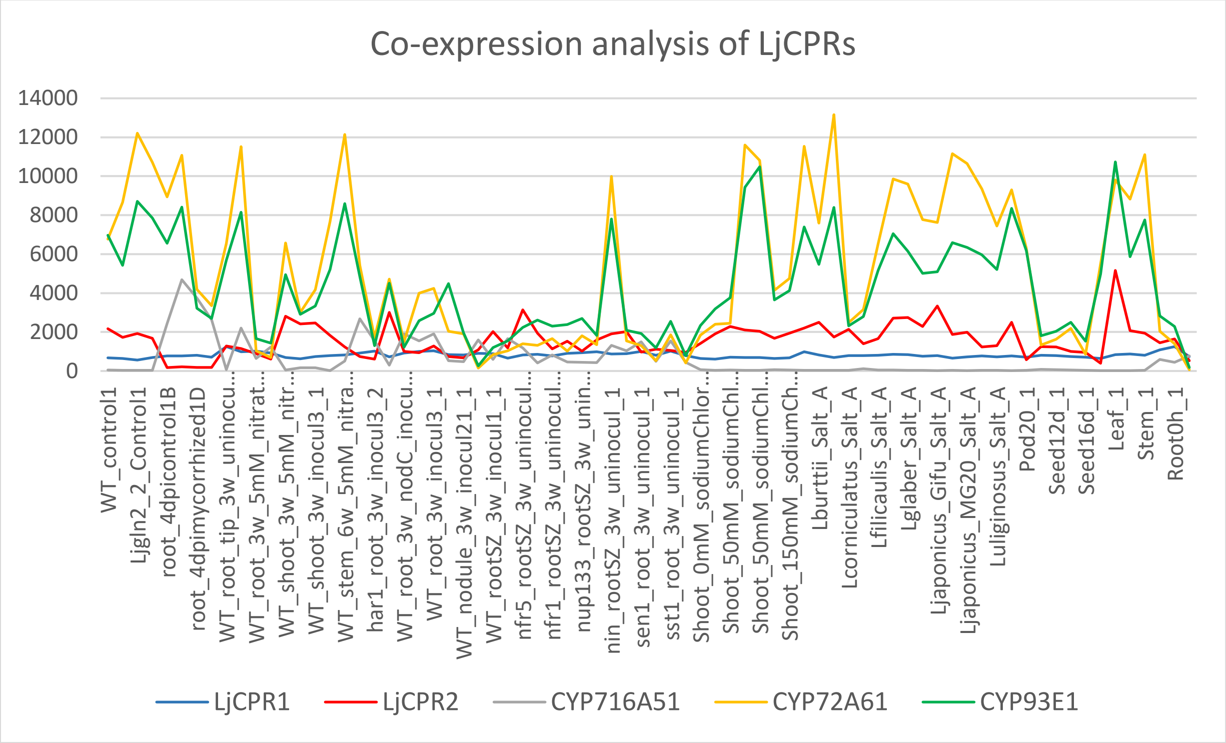

Pearson Correlation Coefficient Table

	LjCPR1	LjCPR2	CYP716A51	CYP72A61	CYP93E1
LjCPR1	1.00	-0.16	0.28	-0.25	-0.25
LjCPR2	-0.16	1.00	-0.52	0.39	0.32
CYP716A51	0.28	-0.52	1.00	-0.15	-0.13
CYP72A61	-0.25	0.39	-0.15	1.00	0.95
CYP93E1	-0.25	0.32	-0.13	0.95	1.00

Table S4.3. Co-expression analysis of CPR class I and II of *G. uralensis*

		**TRINITY ID**	TRINITY_DN18227_c7_g1	TRINITY_DN21433_c2_g4	TRINITY_DN19674_c0_g1	TRINITY_DN18189_c2_g1	TRINITY_DN19088_c1_g2	TRINITY_DN21774_c5_g1
	**Library No.**	**Sample**	**Expression Level**					
			**GuCPR1**	**GuCPR2**	**CYP716A179**	**CYP72A154**	**CYP93E3**	**CYP88D6**
Ramilowski et al., 2013	L1	308-19 strain roots harvested in June: consisted of thickened root of the high-glycyrrhizin-producing strain harvested when the aerial parts were growing	18.91	36.93	0.06	448.7	133.44	590.68
	L2	308-19 strain roots harvested in December: consisted of thickened root of the high-glycyrrhizin-producing strain harvested when the aerial parts were dormant	18.06	72.36	0.07	152.26	0.12	1.28
	L3	87-458 strain roots harvested in June: consisted of thickened root of the low-glycyrrhizin-producing strain 87-458 harvested when the aerial parts were growing	18.65	38.77	0	45.12	19.17	42.88
	L4	308-19 strain leaves harvested in June: consisted of leaves of the high-glycyrrhizin-producing strain harvested when the aerial parts were growing	15.05	52.94	0	12.74	2.89	0.87
Tamura et al., 2017	L5	Tissue-cultured stolon (cultured with NAA containing media)	42.5	72.33	54.46	81.04	216.35	0.78
	L6	Tissue-cultured stolon (cultured with NAA-free media, Sample 1)	43.36	60.96	29.83	107.66	264.65	4.7
	L7	Tissue-cultured stolon (cultured with NAA-free media, Sample 2)	41.1	68.17	36.08	53.03	58.42	0.5
	L8	Tissue-cultured stolon: Drought treatment 3h	45.41	126.34	26.26	164.86	530.86	0.64
	L9	Tissue-cultured stolon: Drought treatment 6h	46.52	84.02	29.73	161.86	273.27	2.79
	L10	Tissue-cultured stolon: Drought treatment 12h	32.27	47.7	22.18	61.81	117.09	0.7
	L11	Tissue-cultured stolon: Drought treatment 24h	44.98	63.09	26.59	34.45	28.7	0.83
	L12	Tissue-cultured stolon: Drought treatment 48h	36.67	49.62	39.15	70.36	50.94	1.28
	L13	Tissue-cultured stolon: Methyl jasmonate (MeJA) 0.5h	41.39	59.25	6.22	76.26	238.07	3.85
	L14	Tissue-cultured stolon: MeJA 1h	43.51	82.49	9.54	67.25	309.2	0.69
	L15	Tissue-cultured stolon: MeJA 2h	51.24	148.57	4.29	95.88	906.07	0.58

L16	Tissue-cultured stolon: MeJA 3h	52.26	170.17	5.41	159.09	1154.23	1.22
L17	Tissue-cultured stolon: MeJA 6h	51.21	138.34	8.93	120.34	553.17	0.51
L18	Tissue-cultured stolon: MeJA 12h	46.81	115.06	15.12	105.56	651.83	0.43
L19	Tissue-cultured stolon: MeJA 24h	53.8	139.33	6.08	289.51	1594.29	1.94
L20	Tissue-cultured stolon: MeJA 48h	50.45	109.88	20	199.15	984.21	3.67
L21	Tissue-cultured stolon: Salicylic acid (SA) 6h	49.32	98.24	22.42	86.05	163.59	4.47
L22	Tissue-cultured stolon: SA 12h	54.27	101.13	10.4	44.3	70.5	0
L23	Tissue-cultured stolon: SA 24h	44.57	59.18	13.65	91.4	194.19	0.44
L24	Tissue-cultured stolon: SA 48h	51.25	53.68	23.5	67.87	89.41	0.34
L25	Tissue-cultured stolon: Yeast extract elicitor (YE) 6h	52.14	117.15	6.62	49.73	142.72	0.11
L26	Tissue-cultured stolon: YE 12h	44.62	71.08	32.13	45.16	150.09	0.9
L27	Tissue-cultured stolon: YE 24h	43.37	61.05	19.5	73.31	223.67	0.28
L28	Tissue-cultured stolon: YE 48h	47.24	64.36	19.74	44.69	176.99	0.32
L29	Tissue-cultured stolon: Gibberellic acid (GA) 6h	46.02	67.53	5.82	58.49	38.43	0.1
L30	Tissue-cultured stolon: GA 12h	45.37	63	6.65	49.37	24.94	0
L31	Tissue-cultured stolon: GA 24h	50.37	63.13	8.41	41.4	14.28	0.13
L32	Tissue-cultured stolon: GA 48h	52.03	72.24	10.57	38.89	3.91	0
L33	Leaves (One-month after acclimatization)	33.25	107.09	0	18.67	1.27	0
L34	Stems (One-month after acclimatization)	31.82	69.39	7.02	65.38	13.23	0.37
L35	Lateral roots (Three-month after acclimatization)	27.02	200.07	81	224.91	403.2	26.36
L36	Main root (Three-month after acclimatization)-Sample 1	30.54	184.31	184.13	831.36	820.47	240.71
L37	Main root (Three-month after acclimatization)-Sample 2	25.01	63.32	137.51	903.45	287.34	309.44

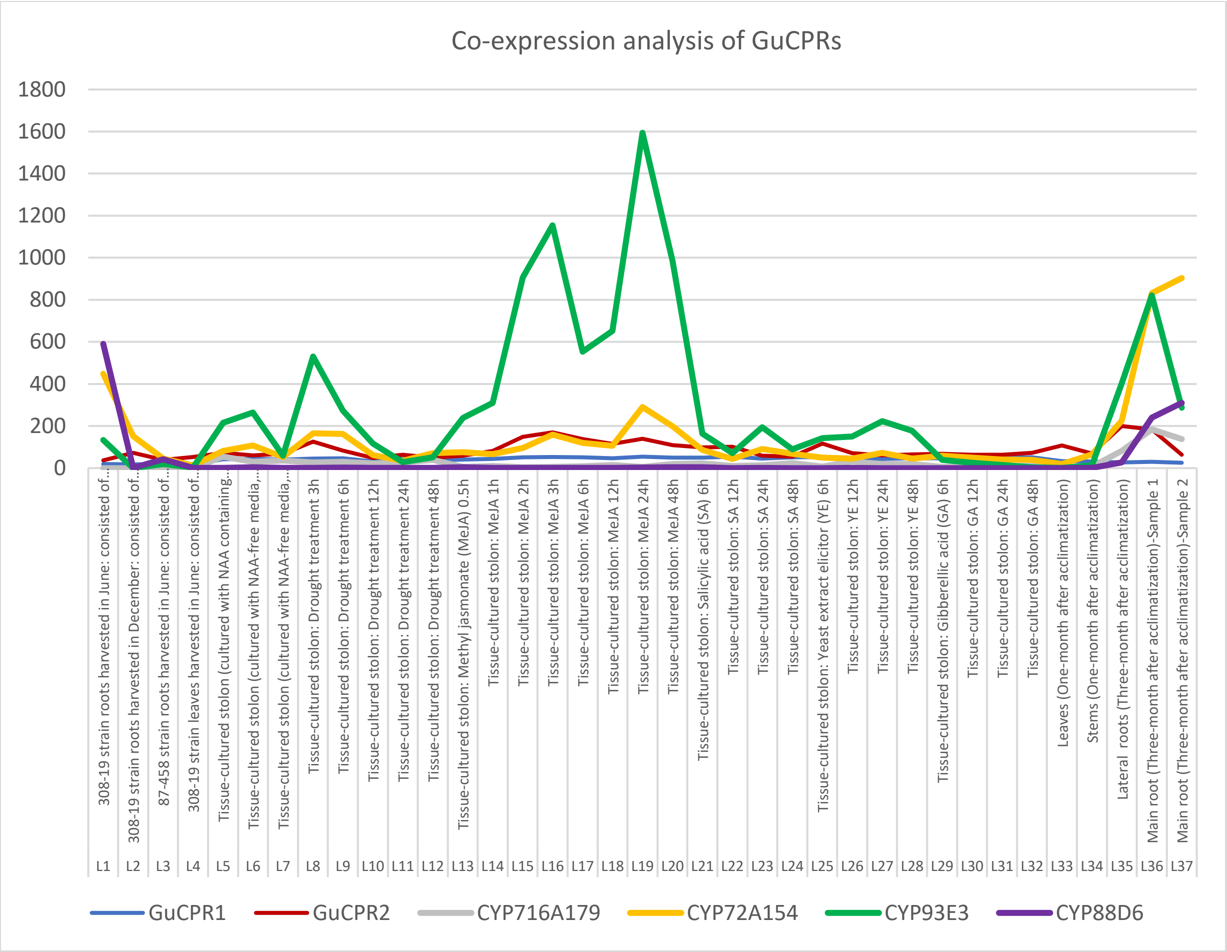

Pearson Correlation Coefficient Table

	**GuCPR1**	**GuCPR2**	**CYP716A179**	**CYP72A154**	**CYP93E3**	**CYP88D6**
**GuCPR1**	1.00	0.25	-0.23	0.73	-0.33	-0.23
**GuCPR2**	0.25	1.00	0.33	-0.08	0.29	0.33
**CYP716A179**	-0.23	0.33	1.00	0.80	0.16	0.38
**CYP72A154**	-0.33	0.29	0.80	1.00	0.36	0.73
**CYP93E3**	0.34	0.70	0.16	0.36	1.00	0.02
**CYP88D6**	-0.48	-0.08	0.38	0.73	0.02	1.00

Table S5. Quality of RNA-seq analysis using 37 *Glycyrrhiza uralensis* RNA-seq data

Number of total reads used in assembly	351,138,706
Number of contigs	226,599
N50 of contigs (bp)	2,438
Average length of contigs (bp)	1,479
Minimum length of contigs (bp)	201
Maximum length of contigs (bp)	17,186

Table S6. Co-expression analysis of CPR class I and II of *M. truncatula*, *L. japonicus*, and *G. uralensis*

**Species**	***Medicago truncatula***			***Lotus japonicus***			***Glycyrrhiza uralensis***		
**Expression Level**	**Treatment**	**Expression level**		**Treatment**	**Expression level**		**Treatment**	**Expression level**	
		MtCPR1	***MtCPR2***		***LjCPR1***	***LjCPR2***		***GuCPR1***	***GuCPR2***
**CPR1 > CPR2**	Infected root nodules	210.84	4.17	Nod21_1	734.69	519.08	In this dataset, CPR2 expression is always higher than CPR1		
	Uninfected root nodules	294.07	3.32	Seed20d_1	635.65	387.96			
	Proximal infection zone root nodules	424.61	4.47	har1_root_3w_inocul3	1027.61	609.97			
	Distal infection zone root nodules	427.57	4.44	root_4dpimycorrhized1D	800.85	180.45			
	Meristem root nodules	481.04	3.48	root_4dpicontrol1B	777.03	174.24			
**CPR2 > CPR1**	Nod SalsC infection	163.04	2559.33	Leaf_1	831.88	5161.46	Roots	27.02	200.07
	Stem A17 20C 100C	467.699	3433.3	Ljaponicus Gifu Salt A	788.02	3341.68	Main root	30.54	184.31
	A17 rhizobia SuffN	555.716	3595.01	har1_shoot_3w_inocul3	728.44	3005.95	308-19 strain roots harvested in winter	18.06	72.36
	Root CRR 96hpi infection	772.606	4724.36	WT_shoot_5mM_nitrate	691.39	2811.11	308-19 strain leaves harvested in summer	15.05	52.94
	Root 2wk Hydroponic 200mM NaCl 24h	1092.85	6606.46	WT_shoot_3w_uninocul	629.65	2410.43	MeJA 3h	52.26	170.17

Table S7. Co-expression analysis of closely correlated genes with CPR class I and II in different tissues of a) *M. truncatula*, b) *L. japonicus*, and c) *G. uralensis.*

a) *M. truncatula*

No.	***MtCPR1* (**Mtr.10548.1.S1_at)			***MtCPR2* (**Mtr.16806.1.S1_at)		
	**Probeset**	**PCC value**	**GO annotation**	**Probeset**	**PCC value**	**GO annotation**
1	Mtr.1520.1.S1_at	0.92	GO:0008150 (biological_process), GO:0003674 (molecular_function)	Mtr.34798.1.S1_at	0.87	GO:0016567 (protein ubiquitination), GO:0005488 (binding), GO:0004842 (ubiquitin-protein ligase activity)
2	Mtr.30028.1.S1_at	0.89	GO:0005515 (protein binding), GO:0006499 (N-terminal protein myristoylation), GO:0008026 (ATP-dependent helicase activity)	Mtr.8676.1.S1_s_at	0.86	GO:0005575 (cellular_component), GO:0008150 (biological_process), GO:0006952 (defense response), GO:0005524 (ATP binding), GO:0005515 (protein binding)
3	Mtr.8673.1.S1_at	0.89	GO:0008152 (metabolic process), GO:0008194 (UDP-glycosyltransferase activity), GO:0016757 (transferase activity, transferring glycosyl groups), GO:0035251 (UDP-glucosyltransferase activity), GO:0010294 (abscisic acid	Mtr.14547.1.S1_at	0.84	GO:0008150 (biological_process), GO:0003824 (catalytic activity), GO:0016208 (AMP binding), GO:0008152 (metabolic process), GO:0003824 (catalytic activity)

			glucosyltransferase activity)			
4	Mtr.14582.1.S1_at	0.88	GO:0019787 (small conjugating protein ligase activity), GO:0005515 (protein binding), GO:0005634 (nucleus), GO:0005737 (cytoplasm), GO:0008150 (biological_process), GO:0005515 (protein binding), GO:0008270 (zinc ion binding)	Mtr.40581.1.S1_at	0.84	GO:0009816 (defense response to bacterium, incompatible interaction), GO:0009817 (defense response to fungus, incompatible interaction), GO:0005515 (protein binding)
5	Mtr.44464.1.S1_at	0.87	GO:0006414 (translational elongation), GO:0005739 (mitochondrion), GO:0003746 (translation elongation factor activity), GO:0006414 (translational elongation), GO:0003746 (translation elongation factor activity), GO:0008135 (translation factor activity, nucleic acid binding)	Mtr.8656.1.S1_s_at	0.84	GO:0003837 (beta-ureidopropionase activity), GO:0006807 (nitrogen compound metabolic process), GO:0003837 (beta-ureidopropionase activity)
6	Mtr.3434.1.S1_at	0.87	GO:0008284 (positive regulation of cell proliferation),	Mtr.40166.1.S1_s_at	0.84	GO:0009699 (phenylpropanoid biosynthetic process),

			GO:0045941 (positive regulation of transcription)			GO:0045548 (phenylalanine ammonia-lyase activity)
7	Mtr.10292.1.S1_at	0.87	GO:0008150 (biological_process), GO:0005488 (binding), GO:0005515 (protein binding)	Mtr.33344.1.S1_at	0.83	GO:0006952 (defense response), GO:0005524 (ATP binding), GO:0005515 (protein binding), GO:0004888 (transmembrane receptor activity)
8	Mtr.51424.1.S1_at	0.86	GO:0019684 (photosynthesis, light reaction), GO:0010207 (photosystem II assembly), GO:0016168 (chlorophyll binding)	Mtr.37895.1.S1_at	0.83	GO:0009617 (response to bacterium), GO:0009620 (response to fungus), GO:0009816 (defense response to bacterium, incompatible interaction), GO:0009817 (defense response to fungus, incompatible interaction), GO:0005515 (protein binding)
9	Mtr.31523.1.S1_at	0.86	GO:0006350 (transcription), GO:0003899 (DNA-directed RNA polymerase activity)	Mtr.11218.1.S1_s_at	0.83	GO:0005524 (ATP binding), GO:0005576 (extracellular region), GO:0005886 (plasma membrane), GO:0006468 (protein amino acid phosphorylation), GO:0019199 (transmembrane receptor protein kinase activity), GO:0006499 (N-terminal protein myristoylation), GO:0006468 (protein amino acid

						phosphorylation), GO:0016301 (kinase activity)
10	Mtr.10754.1.S1_at	0.86	GO:0030612 (arsenate reductase (thioredoxin) activity), GO:0009793 (embryonic development ending in seed dormancy), GO:0003674 (molecular_function)	Mtr.27884.1.S1_at	0.83	GO:0004674 (protein serine/threonine kinase activity), GO:0005887 (integral to plasma membrane), GO:0009737 (response to abscisic acid stimulus), GO:0016301 (kinase activity)

b) *L. japonicus*

No.	***LjCPR1*** (Ljwgs_006504.2_at)			***LjCPR2*** (Ljwgs_068084.1_at)		
	**Probeset**	**PCC value**	**Initial annotation during chip design**	**Probeset**	**PCC value**	**Initial annotation during chip design**
1	TM1224.12_at	0.75	Lotus japonicus similar to At3g08580: adenylate translocator	Ljwgs_109412.1_at	0.68	Lotus japonicus similar to At3g51480: glutamate receptor like protein → defense against pathogens, reproduction, control of stomata aperture and light signal transduction

2	TM1224.12.1_at	0.75	Lotus japonicus similar to At3g08580: adenylate translocator	Ljwgs_016866.2_at	0.67	Lotus japonicus similar to At5g58870: cell division protein - like
3	chr1.TM0430.17.1_at	0.75	Lotus japonicus similar to At5g48900: pectate lyase	TC10072_at	0.66	homologue to UP|Q863B4 (Q863B4) Trefoil factor 3, partial (15%)
4	chr2.CM0056.38_at	0.72	Lotus japonicus similar to At1g60070: hypothetical protein	chr1.CM0591.55_at	0.66	Lotus japonicus similar to Q40983: (Q40983) METALLOENDOPEPTIDASE: amyloid precursor protein catabolic process
5	chr1.CM0105.95_at	0.72	Lotus japonicus similar to At3g54770: RNA binding protein - like	Ljwgs_051871.1_at	0.66	Lotus japonicus similar to At1g50360: myosin, putative
6	TM0759.8_at	0.71	Lotus japonicus similar to At2g26640: putative beta-ketoacyl-CoA synthase	Ljwgs_023382.1_at	0.65	Lotus japonicus similar to O04434: (O04434) PUTATIVE NADPH-CYTOCHROME P450 REDUCTASE
7	Ljwgs_081701.1_at	0.71	Lotus japonicus similar to At4g12420: pollen-specific protein - like predicted GPI-anchored protein	Ljwgs_043693.1_at	0.65	Lotus japonicus similar to At2g32400: ionotropic glutamate receptor (GLR5)

8	Ljwgs_022220.1_at	0.71	Lotus japonicus similar to At4g00710: unknown protein	Ljwgs_074438.1_at	0.65	Lotus japonicus similar to At1g30360: ERD4 protein (ERD4: Early-responsive to dehydration stress protein)
9	chr5.CM0328.80_at	0.71	Lotus japonicus similar to At5g08680: H+-transporting ATP synthase beta chain (mitochondrial) -like protein	chr1.CM0591.54_at	0.64	Lotus japonicus similar to At5g42390: pitrilysin
10	Ljwgs_089550.1_at	0.71	Lotus japonicus similar to At4g28650: receptor protein kinase-like protein	Ljwgs_058241.1_at	0.63	Lotus japonicus similar to At2g39190: ABC transporter like protein

c) *G. uralensis*

No.	***GuCPR1*** (TRINITY_DN18227_c7_g1)			***GuCPR2*** (TRINITY_DN21433_c2_g4)		
	**Unigene ID**	**PCC value**	**Gene description**[1]	**Unigene ID**	**PCC value**	**Gene description**[1]
1	TRINITY_DN22119 _c0_g2	0.88	Tudor2, AtTudor2, TSN2 | Arabidopsis thaliana TUDOR-SN protein 2, TUDOR-SN protein 2	TRINITY_DN25698 _c0_g1	0.86	PAP26, ATPAP26 | purple acid phosphatase 26, PURPLE ACID PHOSPHATASE 26
2	TRINITY_DN19174 _c0_g1	0.86	AVA-P3, VHA-C3, ATVHA-C3 | vacuolar-type H(+)-ATPase	TRINITY_DN17844 _c6_g2	0.85	4CL3 | 4-coumarate:CoA ligase 3

3	TRINITY_DN20160 _c1_g3	0.85	SHY3, ATKT2, KT2, KUP2, ATKUP2, TRK2 | potassium transporter 2	TRINITY_DN17669 _c0_g6	0.85	No symbol available | no full name available
4	TRINITY_DN13072 _c0_g1	0.85	No symbol available | no full name available | chr3:6325858-6327666	TRINITY_DN11745 _c0_g1	0.84	SG1 | SLOW GREEN 1
5	TRINITY_DN19072 _c0_g3	0.85	ATPAH2, PAH2 | PHOSPHATIDIC ACID PHOSPHOHYDROLASE 2, phosphatidic acid phosphohydrolase 2	TRINITY_DN22602 _c1_g3	0.84	EFE, ACO4, EAT1 | ethylene forming enzyme, ethylene-forming enzyme
6	TRINITY_DN19747 _c2_g2	0.85	SAPX | stromal ascorbate peroxidase	TRINITY_DN20044 _c5_g1	0.83	JAZ11, TIFY3A | jasmonate-zim-domain protein 11
7	TRINITY_DN23561 _c2_g1	0.84	Symbols: FUT12, FUCT2, ATFUT12, FUCTB | fucosyltransferase 12	TRINITY_DN19740 _c1_g1	0.83	METK1, SAM-1, AtSAM1, SAM1, MAT1 | S-adenosylmethionine synthetase 1, S-ADENOSYLMETHIONINE SYNTHETASE-1
8	TRINITY_DN21594 _c5_g2	0.83	Symbols: no symbol available | no full name available | chr1:25028538-25029857 REVERSE LENGTH=1320	TRINITY_DN24462 _c0_g1	0.81	No symbol available | no full name available |

9	TRINITY_DN15591_c0_g3	0.83	Symbols: ARO4 | armadillo repeat only 4 | chr3:9769666-9772112 FORWARD LENGTH=2447	TRINITY_DN18249_c1_g4	0.81	HSFA1B, HSF3, ATHSF3, ATHSFA1B | ARABIDOPSIS HEAT SHOCK FACTOR 3, CLASS A HEAT SHOCK FACTOR 1B, heat shock factor 3, ARABIDOPSIS THALIANA CLASS A HEAT SHOCK FACTOR 1
10	TRINITY_DN17892_c5_g1	0.82	Symbols: CSE, LysoPL2, AtMAGL3 | Caffeoyl Shikimate Esterase, lysophospholipase 2	TRINITY_DN22602_c1_g2	0.81	EFE, ACO4, EAT1 | ethylene forming enzyme, ethylene-forming

[1]Gene description was obtained from **by blastn query on Araport11 transcripts (DNA) sequences**. Query was performed by the The Arabidopsis Information Resource (TAIR) using DNA contig sequences from the highly correlated *G. uralensis* unigenes. For full BLAST options and parameters, refer to the NCBI BLAST Documentation. BLAST top hit with > 70% identity was chosen.

Table S8. Probeset ID used for co-expression analysis and PCC calculation

Gene	Probeset ID	Transcriptomic Database
MtCYP716A12	Mtr.43018.1.S1_at	mtgea.noble.org/v3
MtCYP72A63	Mtr.46721.1.S1_x_at	mtgea.noble.org/v3
MtCYP93E2	Mtr.8618.1.S1_at	mtgea.noble.org/v3
MtCPR1	Mtr.10548.1.S1_at	mtgea.noble.org/v3
MtCPR2	Mtr.16806.1.S1_at	mtgea.noble.org/v3
LjCYP716A51	Ljwgs_038251.2_at	lotus.au.dk
LjCYP72A61	chr3.TM0797.5_at	lotus.au.dk
LjCYP93E1	Ljwgs_008809.1_at	lotus.au.dk
LjCPR1	Ljwgs_006504.2_at	lotus.au.dk
LjCPR2-1 & LjCPR2-2	Ljwgs_068084.1_at	lotus.au.dk
GuCYP716A179	TRINITY_DN19674_c0_g1	In-house data
GuCYP72A154	TRINITY_DN18189_c2_g1	In-house data
GuCYP93E3	TRINITY_DN19088_c1_g2	In-house data
GuCYP88D6	TRINITY_DN21774_c5_g1	In-house data
GuCPR1	TRINITY_DN18227_c7_g1	In-house data
GuCPR2	TRINITY_DN21433_c2_g4	In-house data

Table S9. Correlation strength between different CPR class and CYP families in *L. japonicus* based on Gifu v2.0 genome version.

Gene ID	Gene	*LjCPR1*	*LjCPR2.2*	*LjCPR2.1*	*CYP716A51*	*CYP72A61*	*CYP93E1*
LotjaGi1g1v0345200	*LjCPR1*	1.00					
LotjaGi4g1v0301300	*LjCPR2.2*	0.07	1.00				
LotjaGi4g1v0301400	*LjCPR2.1*	0.01	**0.59**	1.00			
LotjaGi4g1v0438900	*CYP716A51*	**0.44**	-0.31	-0.39	1.00		
LotjaGi3g1v0557600	*CYP72A61*	-0.25	-0.18	-0.33	0.16	1.00	
LotjaGi1g1v0588600	*CYP93E1*	0.22	-0.37	-0.42	**0.52**	**0.77**	1.00

Table S10. DDBJ run accession numbers of 37 *Glycyrrhiza uralensis* RNA-seq data

Library No.	Sample	SRA Run Accession No.	Reference
L1	308-19 strain roots harvested in June: consisted of thickened root of the high-glycyrrhizin-producing strain harvested when the aerial parts were growing	DRR006519 and DRR006520	Ramilowski et al., 2013
L2	308-19 strain roots harvested in December: consisted of thickened root of the high-glycyrrhizin-producing strain harvested when the aerial parts were dormant	DRR006521 and DRR006522	Ramilowski et al., 2013
L3	87-458 strain roots harvested in June: consisted of thickened root of the low-glycyrrhizin-producing strain 87-458 harvested when the aerial parts were growing	DRR006523 and DRR006524	Ramilowski et al., 2013
L4	308-19 strain leaves harvested in June: consisted of leaves of the high-glycyrrhizin-producing strain harvested when the aerial parts were growing	DRR006525 and DRR006526	Ramilowski et al., 2013
L5	Tissue-cultured stolon (cultured with NAA containing media)	DRR066062	Tamura et al., 2017
L6	Tissue-cultured stolon (cultured with NAA-free media, Sample 1)	DRR302263	This study
L7	Tissue-cultured stolon (cultured with NAA-free media, Sample 2)	DRR302264	This study
L8	Tissue-cultured stolon: Drought treatment 3h	DRR302265	This study
L9	Tissue-cultured stolon: Drought treatment 6h	DRR302266	This study
L10	Tissue-cultured stolon: Drought treatment 12h	DRR302267	This study
L11	Tissue-cultured stolon: Drought treatment 24h	DRR302268	This study
L12	Tissue-cultured stolon: Drought treatment 48h	DRR302269	This study
L13	Tissue-cultured stolon: Methyl jasmonate (MeJA) 0.5h	DRR302270	This study
L14	Tissue-cultured stolon: MeJA 1h	DRR302271	This study
L15	Tissue-cultured stolon: MeJA 2h	DRR302272	This study
L16	Tissue-cultured stolon: MeJA 3h	DRR302273	This study
L17	Tissue-cultured stolon: MeJA 6h	DRR302274	This study
L18	Tissue-cultured stolon: MeJA 12h	DRR302275	This study
L19	Tissue-cultured stolon: MeJA 24h	DRR302276	This study
L20	Tissue-cultured stolon: MeJA 48h	DRR302277	This study
L21	Tissue-cultured stolon: Salicylic acid (SA) 6h	DRR302278	This study
L22	Tissue-cultured stolon: SA 12h	DRR302279	This study
L23	Tissue-cultured stolon: SA 24h	DRR302280	This study
L24	Tissue-cultured stolon: SA 48h	DRR302281	This study
L25	Tissue-cultured stolon: Yeast extract elicitor (YE) 6h	DRR302282	This study
L26	Tissue-cultured stolon: YE 12h	DRR302283	This study
L27	Tissue-cultured stolon: YE 24h	DRR302284	This study
L28	Tissue-cultured stolon: YE 48h	DRR302285	This study
L29	Tissue-cultured stolon: Gibberellic acid (GA) 6h	DRR302286	This study

L30	Tissue-cultured stolon: GA 12h	DRR302287	This study
L31	Tissue-cultured stolon: GA 24h	DRR302288	This study
L32	Tissue-cultured stolon: GA 48h	DRR302289	This study
L33	Leaves (One-month after acclimatization)	DRR302290	This study
L34	Stems (One-month after acclimatization)	DRR302291	This study
L35	Lateral roots (Three-month after acclimatization)	DRR302292	This study
L36	Main root (Three-month after acclimatization)-Sample 1	DRR302293	This study
L37	Main root (Three-month after acclimatization)-Sample 2	DRR302294	This study

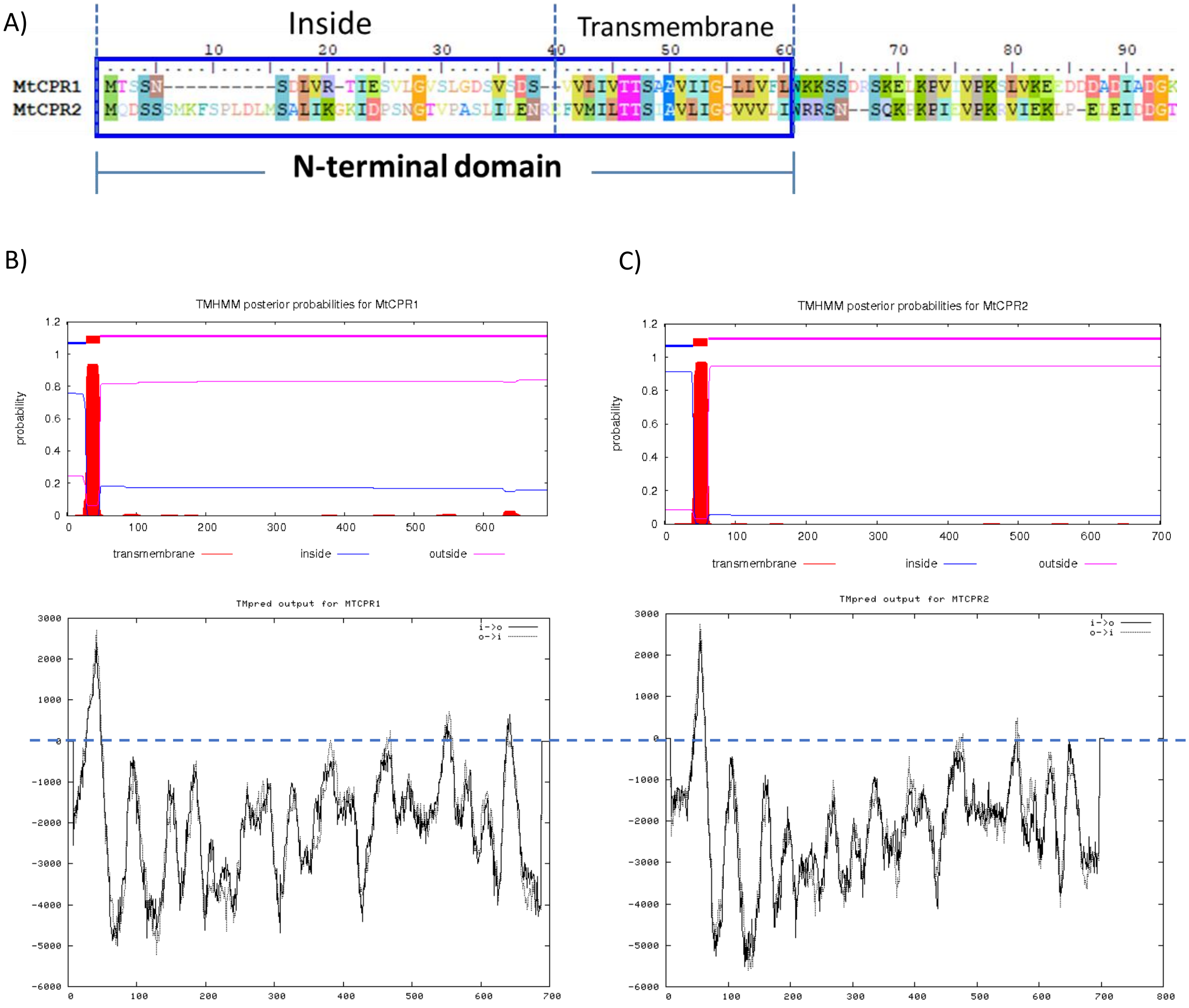

Figure S1. In silico transmembrane helix prediction of MtCPR1 and MtCPR2 (http://www.cbs.dtu.dk/services/TMHMM/). (A) The sequence of MtCPR1 and MtCPR2 position of protein helix inside, transmembrane, and outside of the endoplasmic reticulum (ER) respectively. (B) Amino acid of MtCPR1 number 1-26 is located inside the ER, 27-46 is the transmembrane helix, and 47-692 is located outside the ER. (C) Amino acid of MtCPR2 number 1-40 is located inside the ER, 41-60 is the transmembrane helix, and 61-701 is located outside the ER.

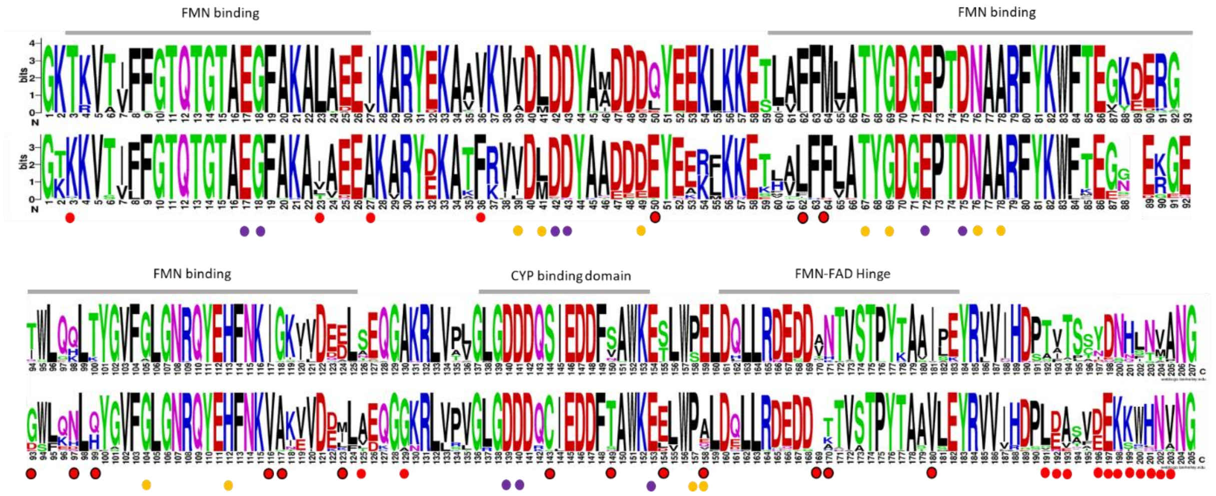

Figure S2. Motif analysis of 37 sequences of each CPR class I and II from 24 legume species. Red circles indicate the different residue that are conserved in each CPR class I and II. Purple circles indicate acidic residue formerly reported to be important in CYP:CPR interaction in human CPR (hCPR). Yellow circles indicate point mutations in hCPR that are reported to improve interaction with a specific CYP. Motif logo was created by WebLogo online software (https://weblogo.berkeley.edu/logo.cgi).

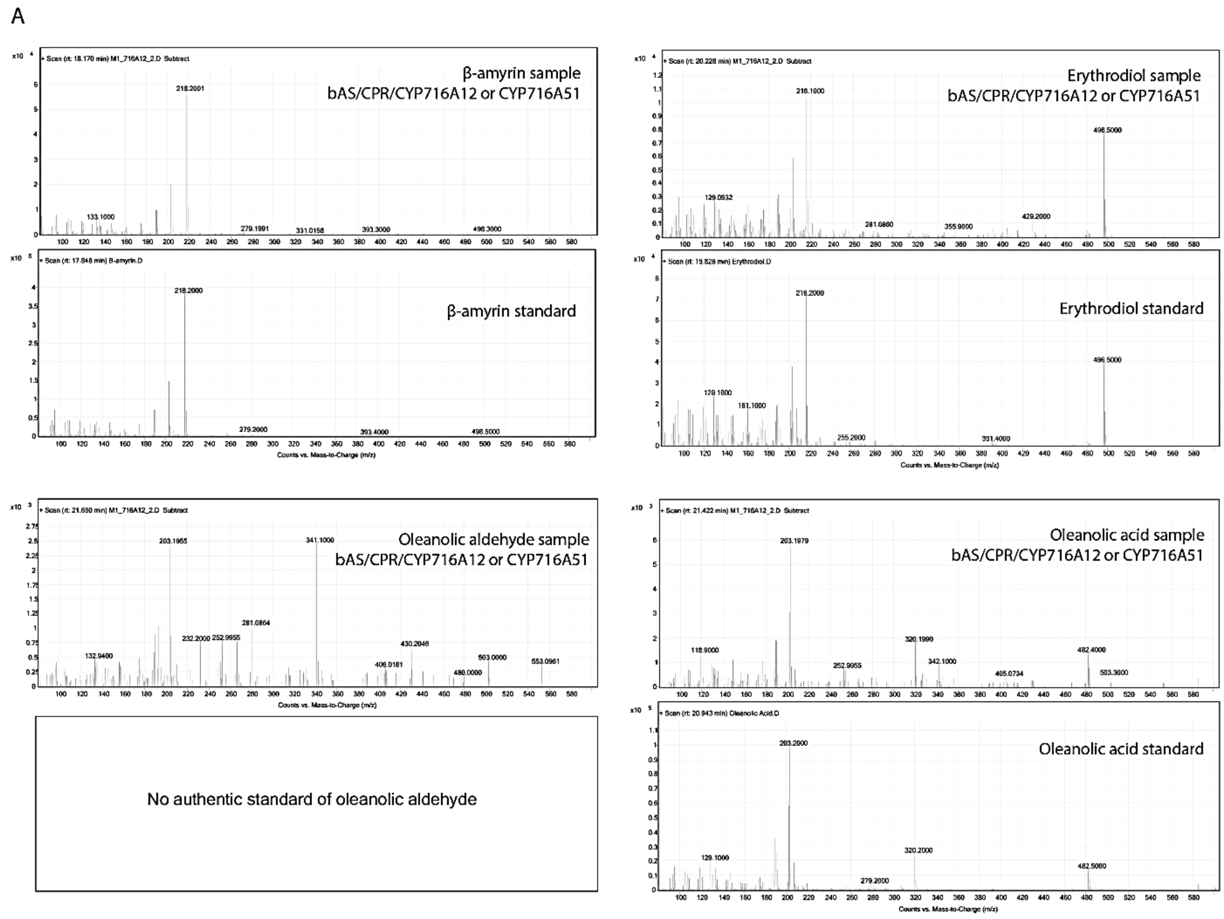

Figure S3. Mass spectra of target compounds and authentic standards of β-amyrin derivatives at (A) C-28, (B) C-30, (C) C-24, (D) C-22, and (E) α-amyrin and (F) lupeol derivatives at C-28.

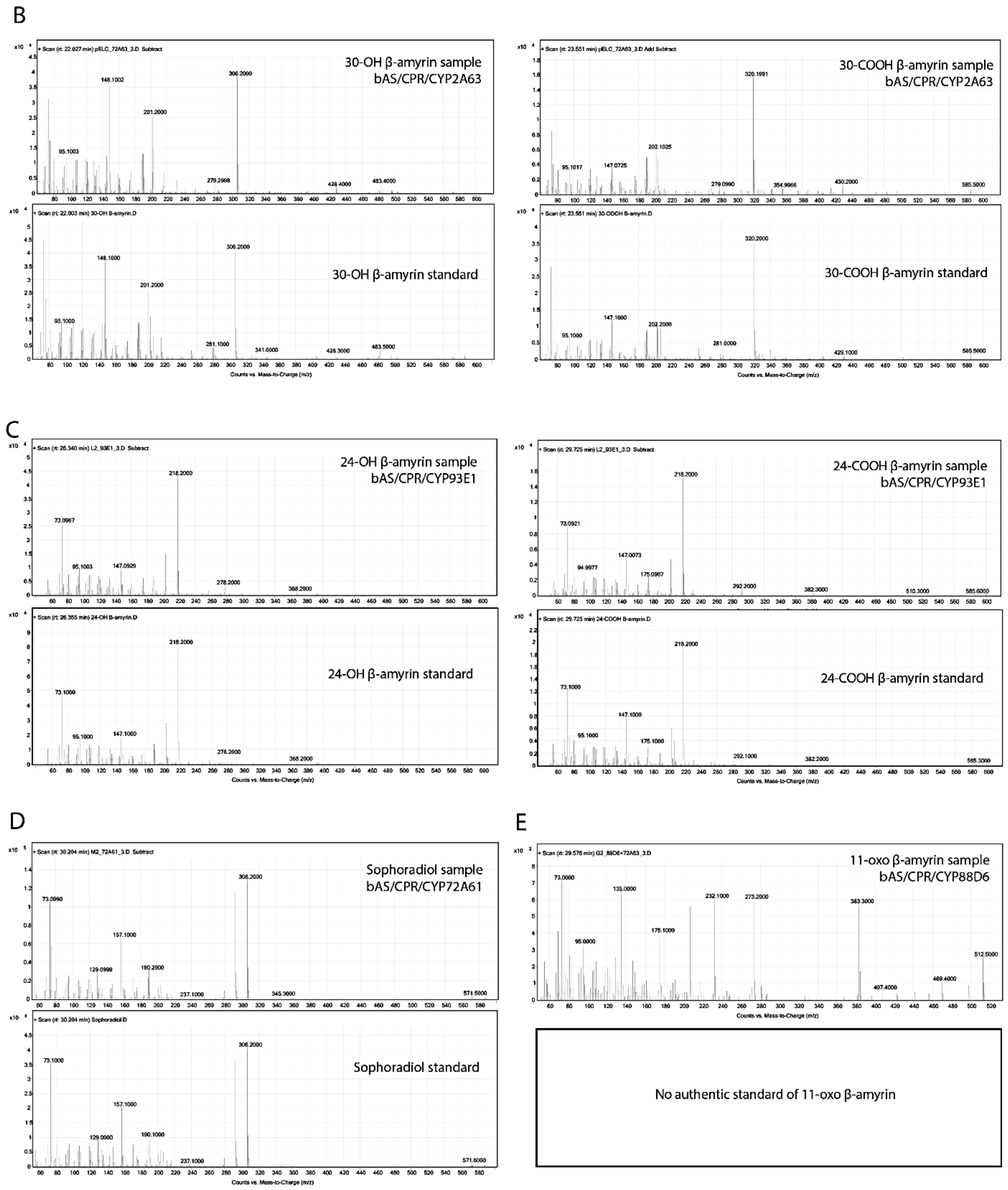

Figure S3. Cont.

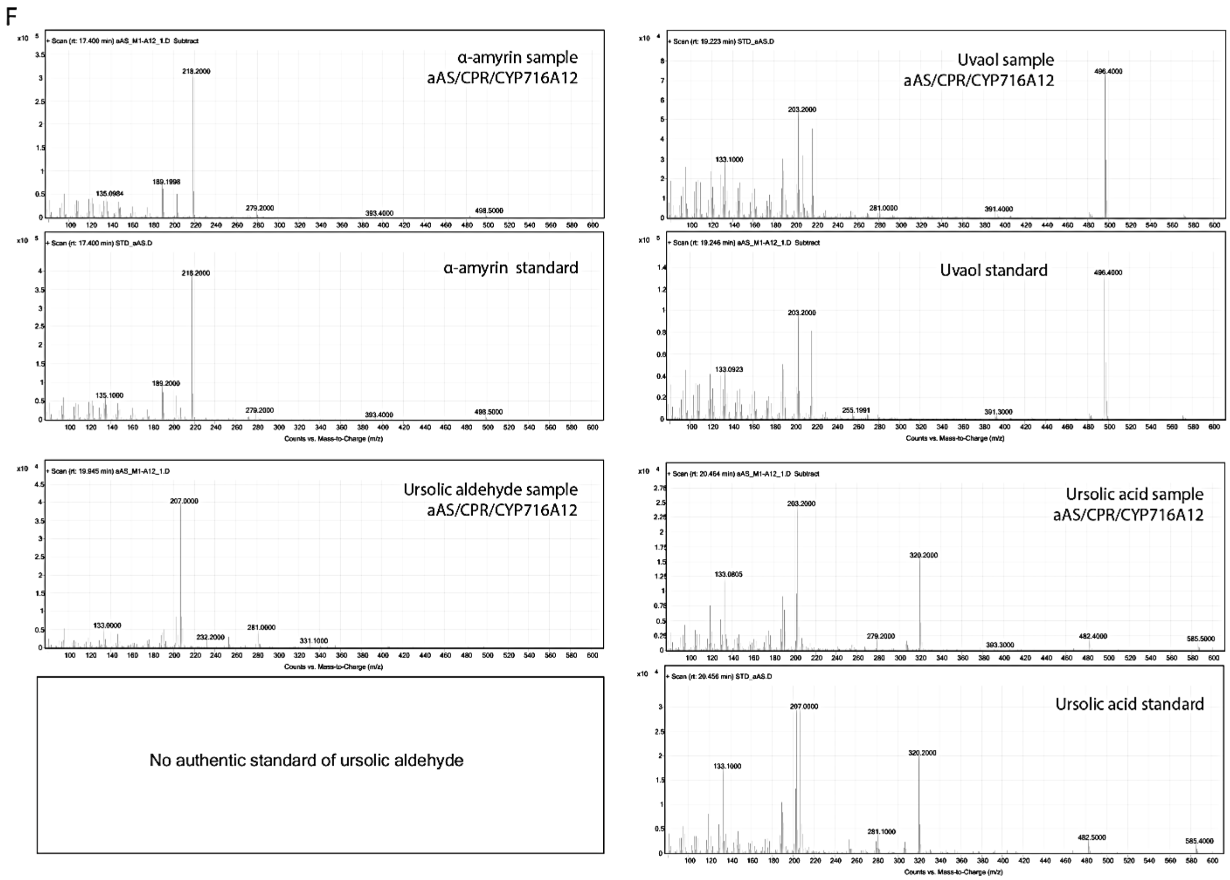

Figure S3. Cont.

G

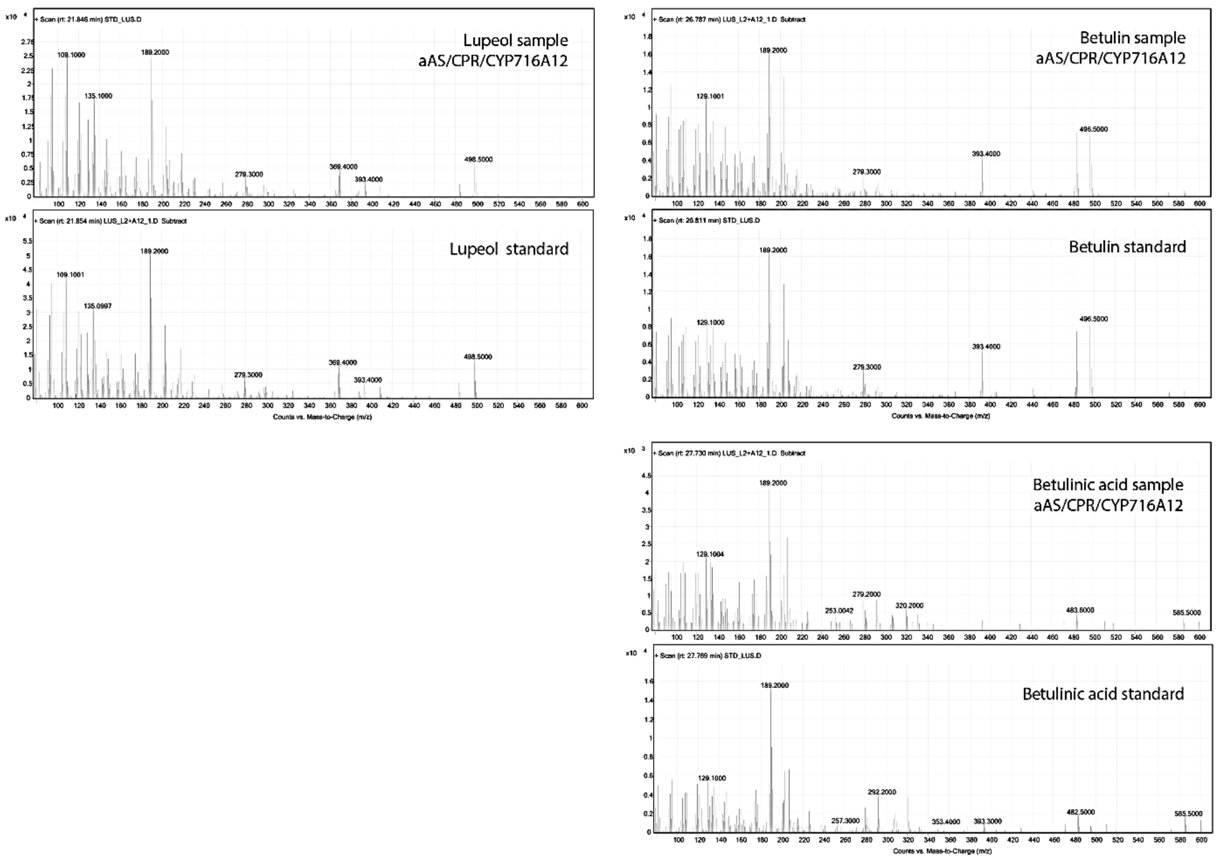

Figure S3. Cont.

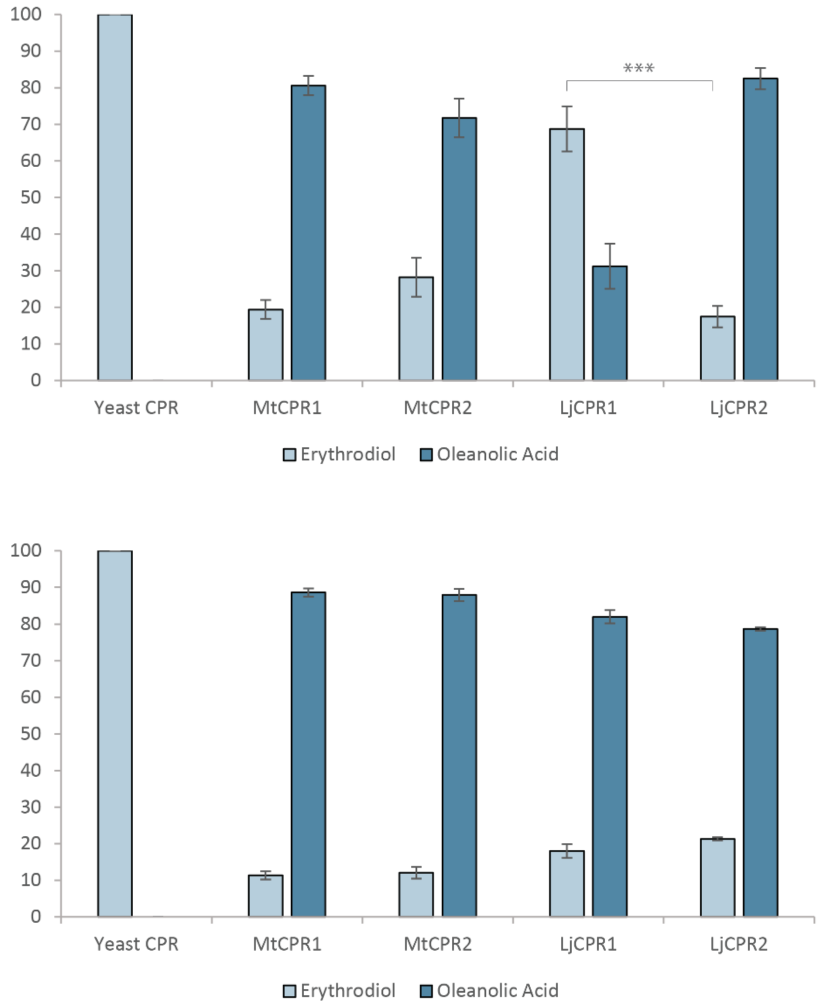

Figure S4. The relative amount of triterpenoid produced by co-expression of A) *MtCYP716A12* and B) *LjCYP716A51* and different CPRs from *M. truncatula* and *L. japonicus* in yeast feeding assay, by supplementing yeast INV*Sc1* strain with 10 μM of erythrodiol as substrate. Triterpenoids content were measured relative to uvaol as internal standard. Data have been presented as mean ± SE (n=3). nd, signal below detection limit. Single-factor ANOVA was used for statistical comparisons. Values were considered statistically significant at *P<0.05, **P<0.01, and ***P<0.001.

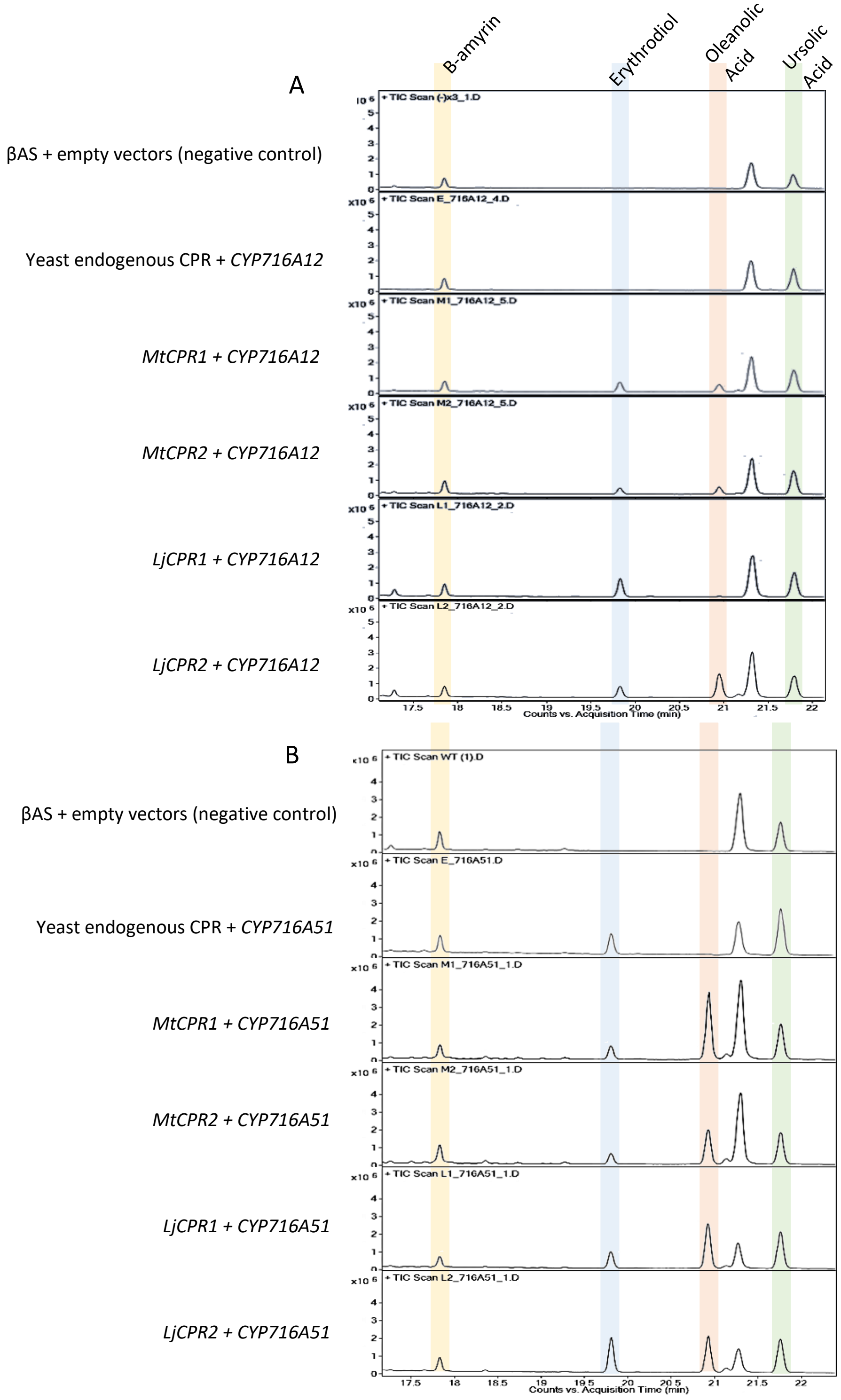

Figure S5. GC-MS chromatogram of triterpenoids extracted from β-amyrin-producing *INVSc1* yeast harboring *MtCPRs* and *LjCPRs* paired with A) *CYP716A12* and B) *CYP716A51*. All samples were cultured in different time, and ran in GCMS at the same time using HP 5-MS column with common method with ursolic acid as internal standard.

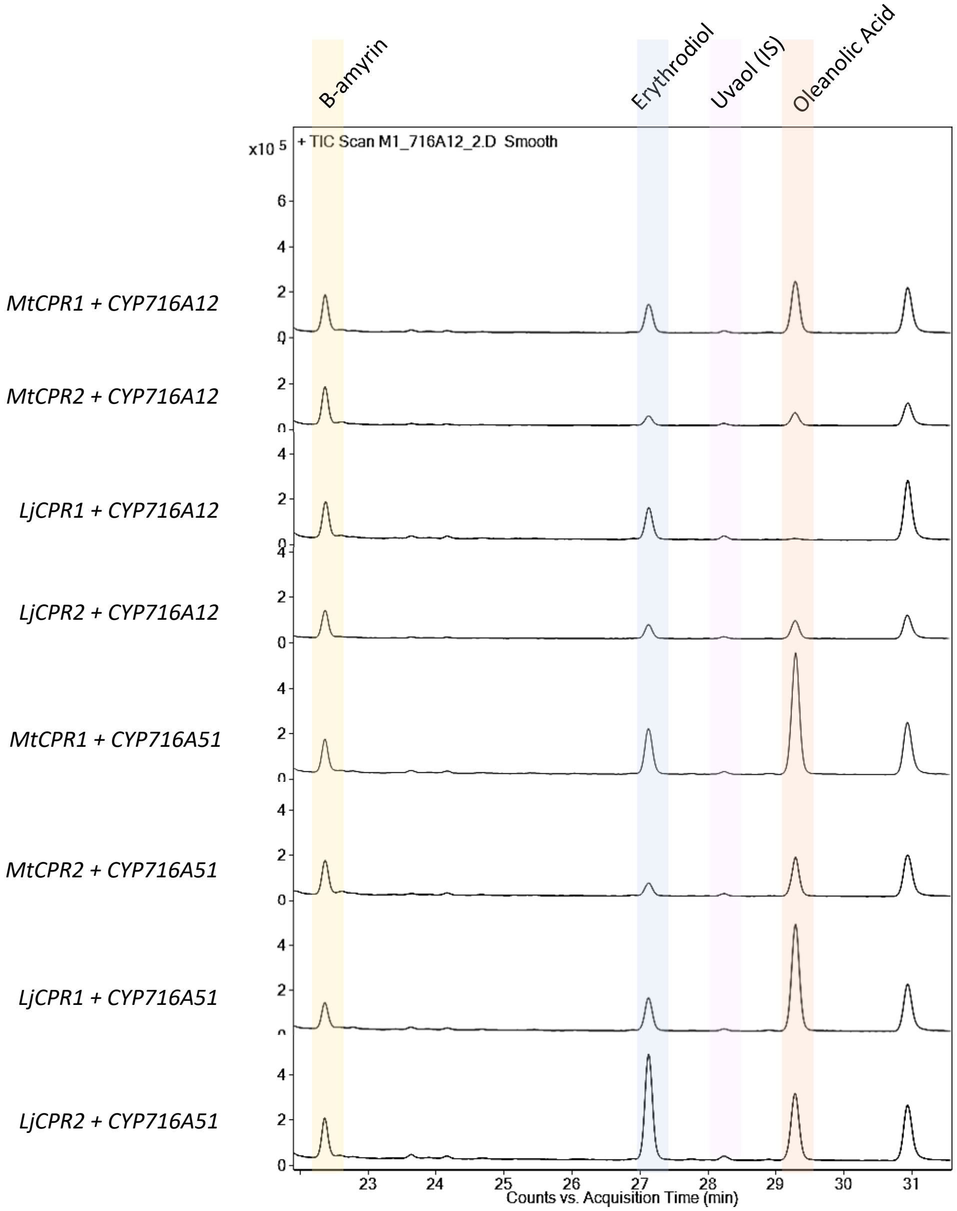

Figure S6. GC-MS chromatogram of triterpenoids extracted from β-amyrin-producing *INVSc1* yeast harboring *MtCPRs* and *LjCPRs* paired with *CYP716A12* and *CYP716A51*. All samples were cultured in and ran in GCMS at the same time using HP-5 MS columin with optimized method with uvaol as internal standard.

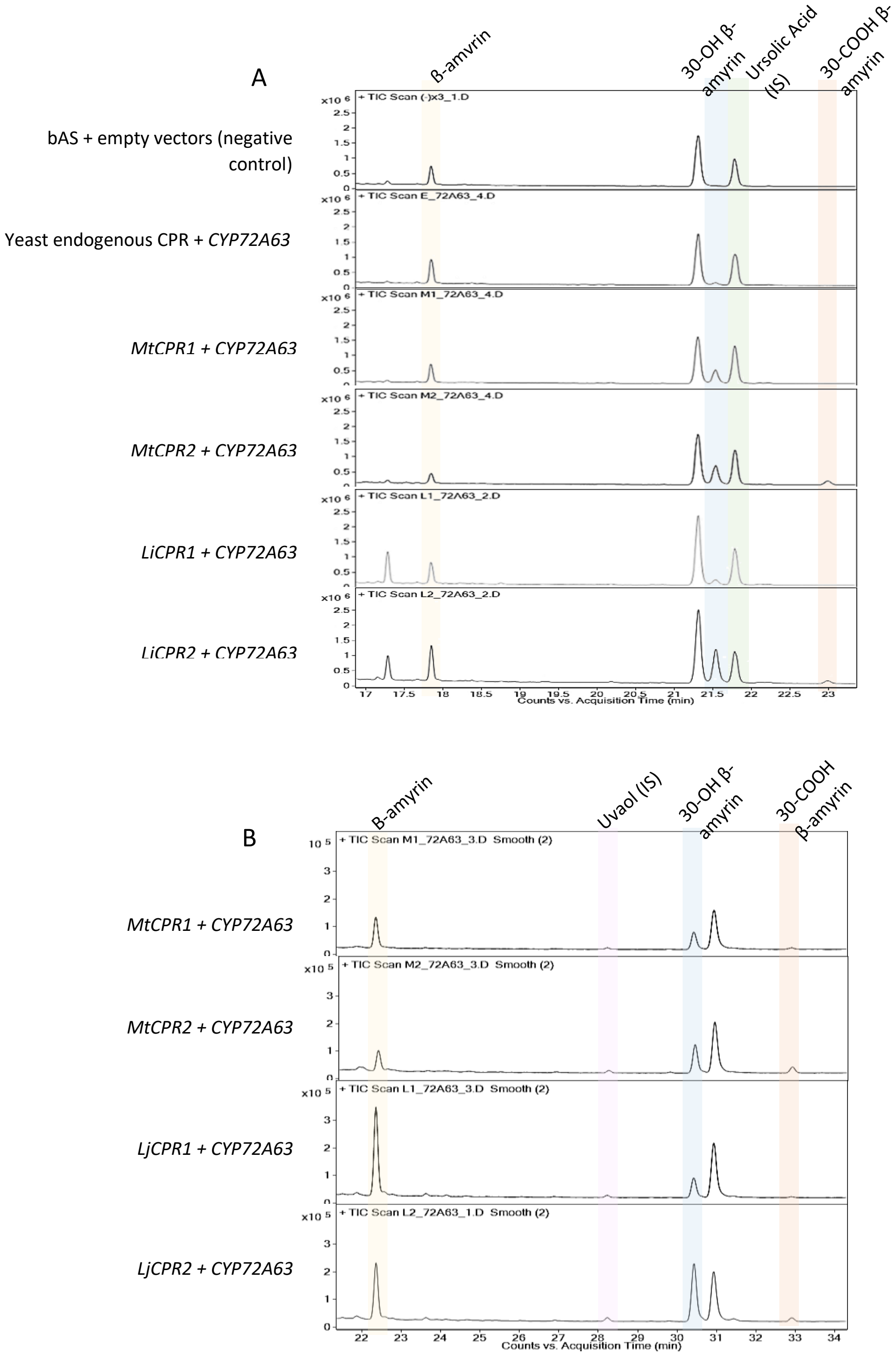

Figure S7. GC-MS chromatogram of triterpenoids extracted from β-amyrin-producing *INVSc1* yeast harboring *MtCPRs* and *LjCPRs* paired with *CYP72A63* using HP 5-MS column with (A) common method with ursolic acid as internal standard and (B) optimized method with uvaol as internal standard.

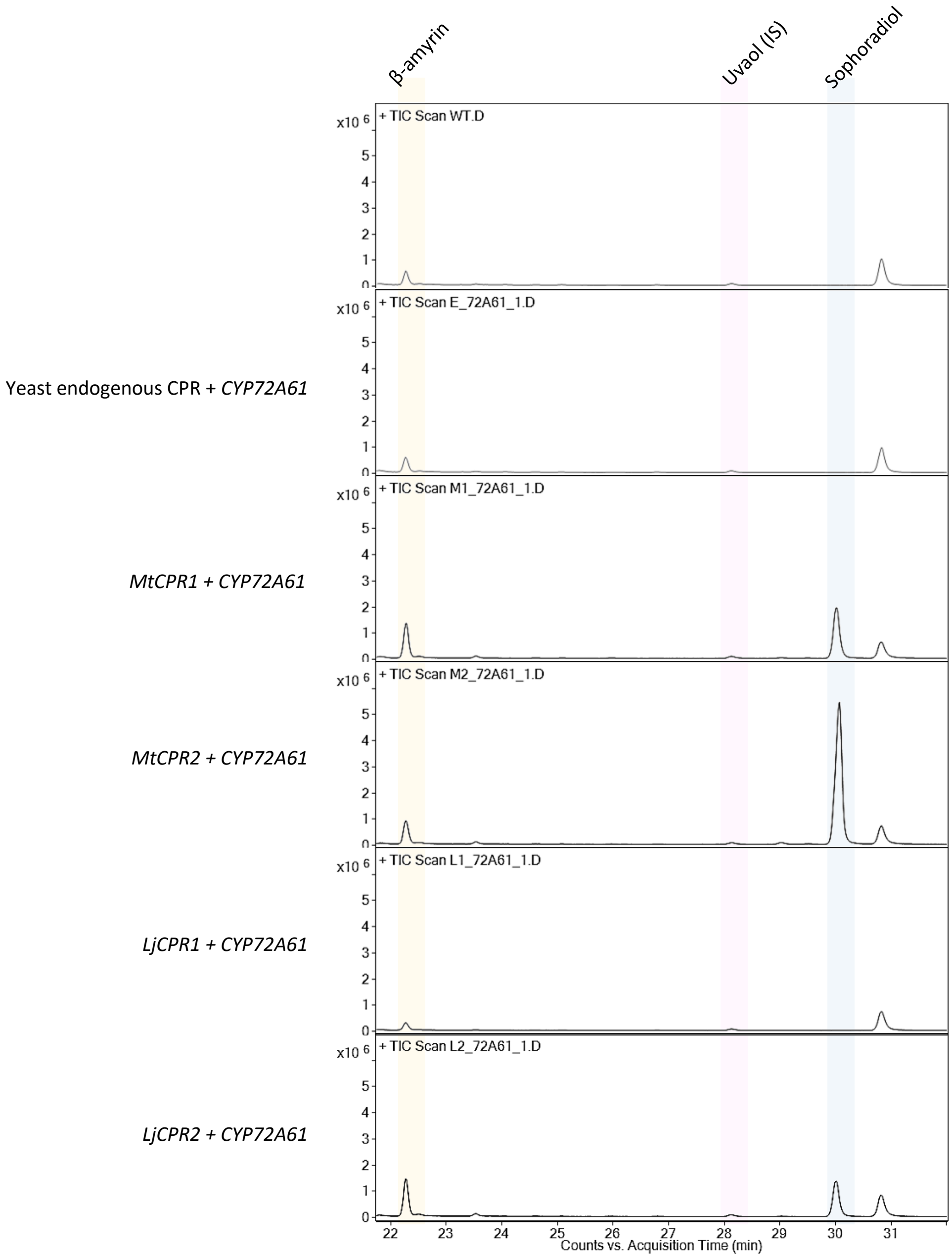

Figure S8. GC-MS chromatogram of triterpenoids extracted from β-amyrin-producing *INVSc1* yeast harboring *MtCPRs* and *LjCPRs* paired with *CYP72A61* using HP 5-MS column with optimized method with uvaol as internal standard.

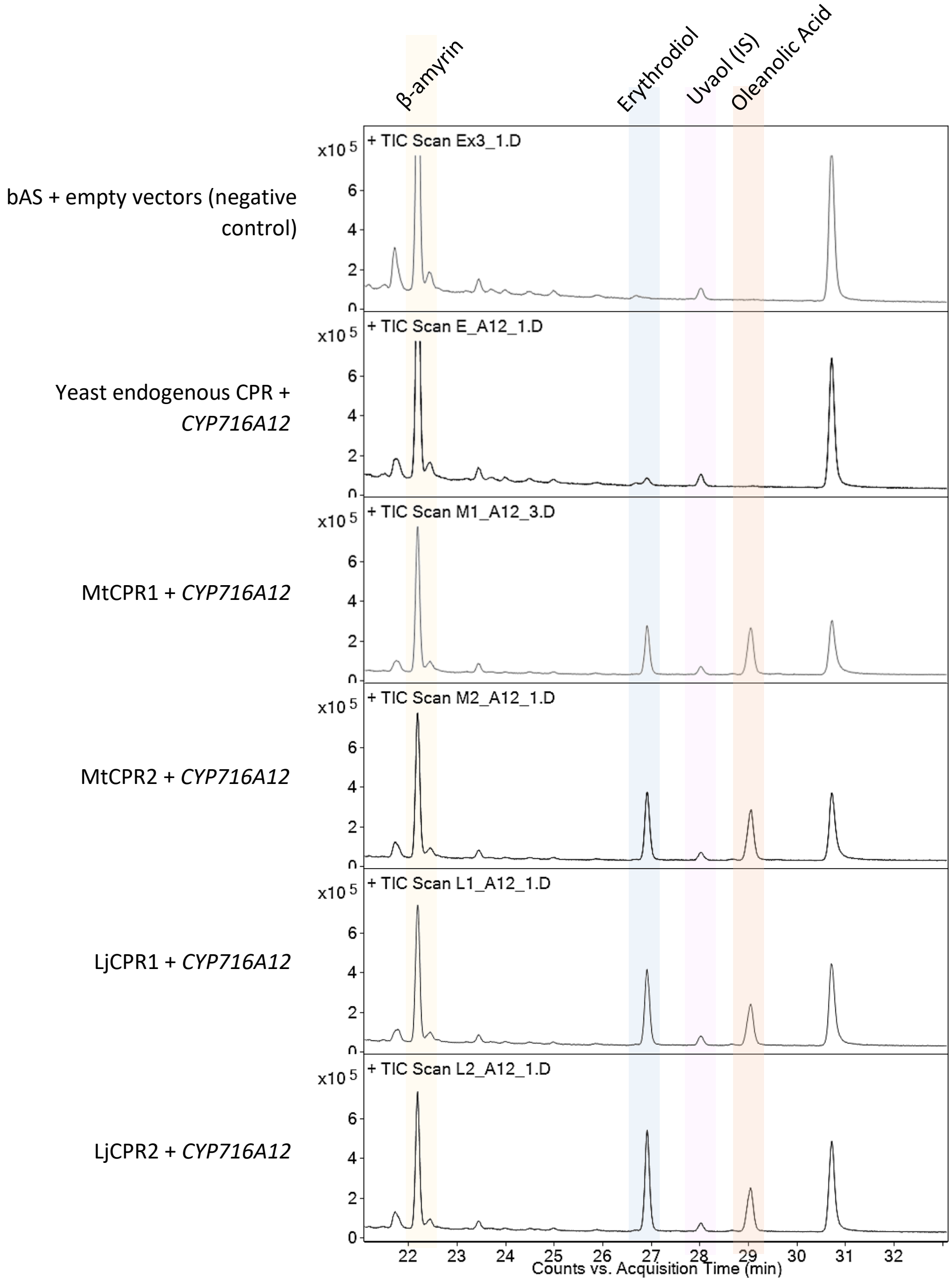

Figure S9. GC-MS chromatogram of triterpenoids extracted from β-amyrin-producing *PSIII* yeast harboring *MtCPRs* and *LjCPRs* paired with *CYP716A12* using HP 5-MS column with optimized method with uvaol as internal standard.

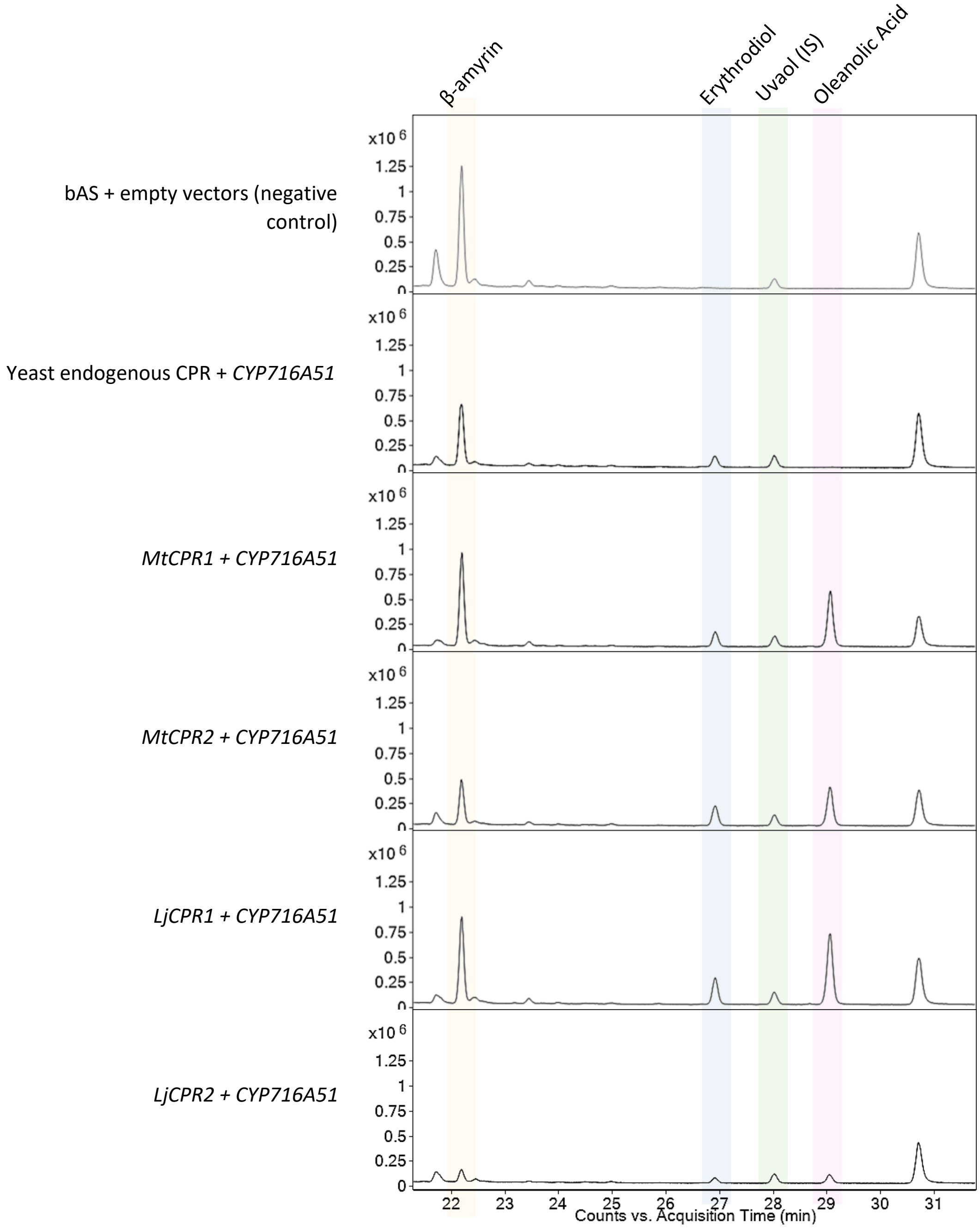

Figure S10. GC-MS chromatogram of triterpenoids extracted from β-amyrin-producing *PSIII* yeast harboring *MtCPRs* and *LjCPRs* paired with *CYP716A51* using HP 5-MS column with optimized method with uvaol as internal standard.

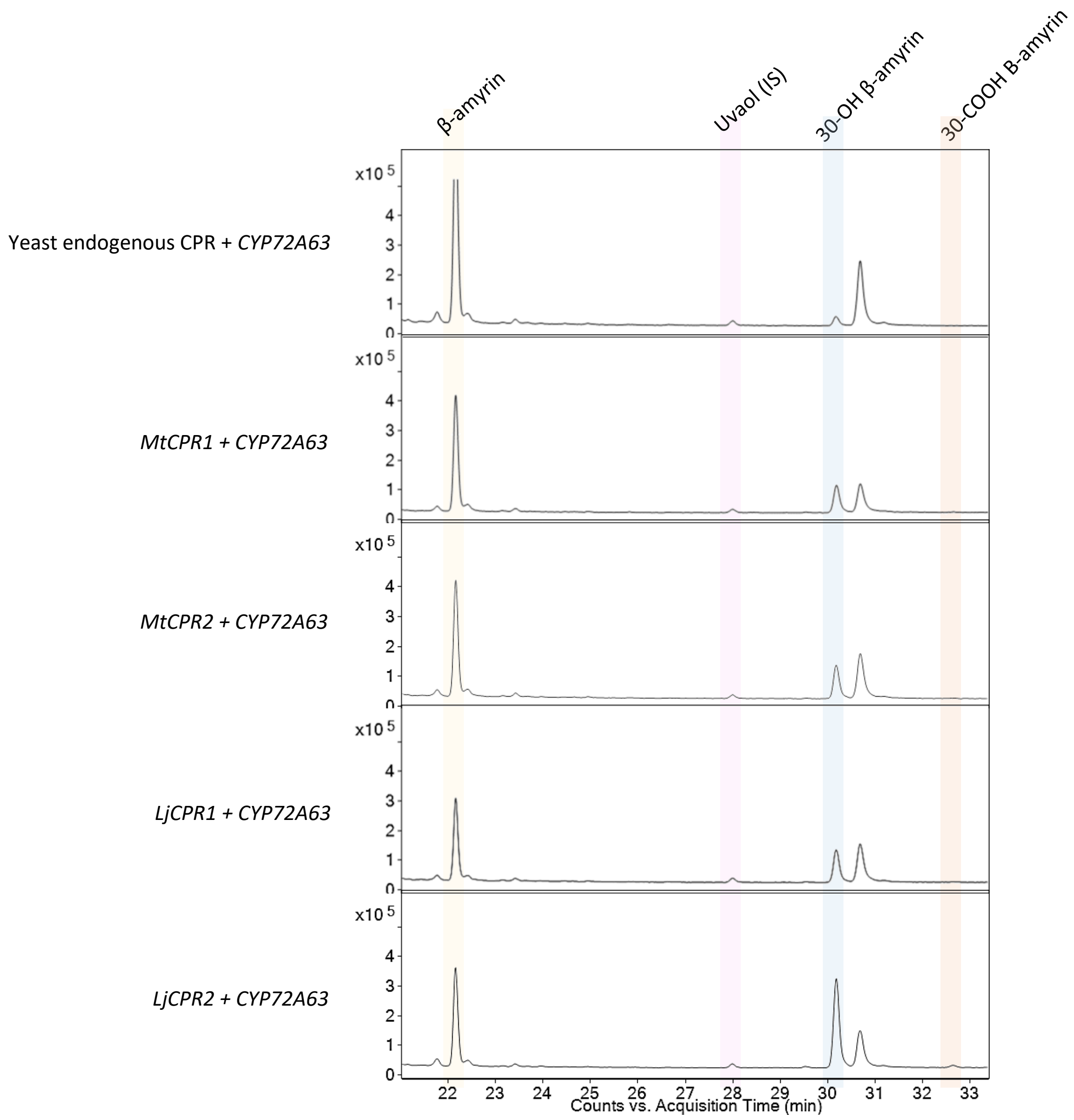

Figure S11. GC-MS chromatogram of triterpenoids extracted from β-amyrin-producing *PSIII* yeast harboring *MtCPRs* and *LjCPRs* paired with *CYP72A63* using HP 5-MS column with optimized method with uvaol as internal standard.

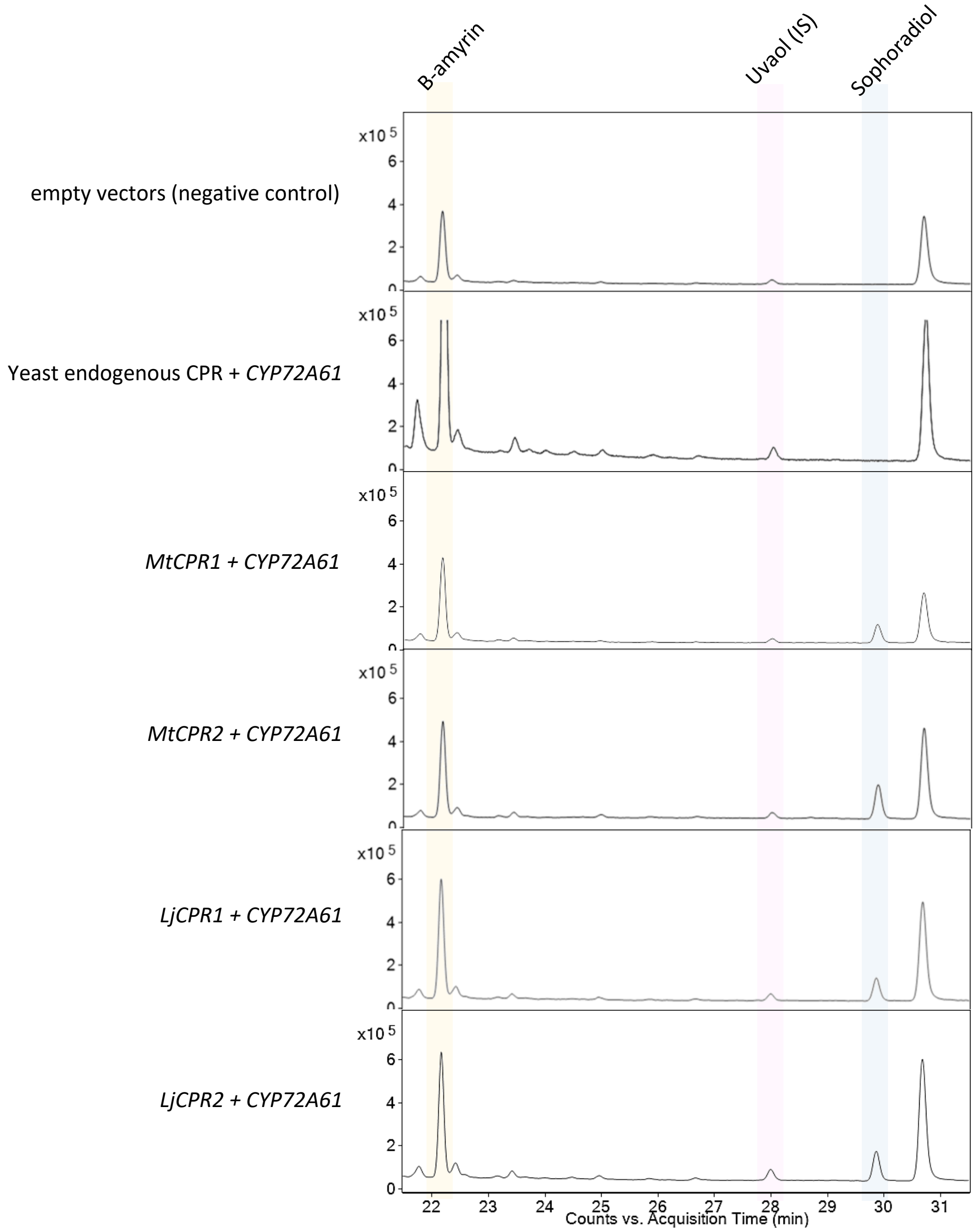

Figure S12. GC-MS chromatogram of triterpenoids extracted from β-amyrin-producing *PSIII* yeast harboring *MtCPRs* and *LjCPRs* paired with *CYP72A61* using HP 5-MS column with optimized method with uvaol as internal standard.

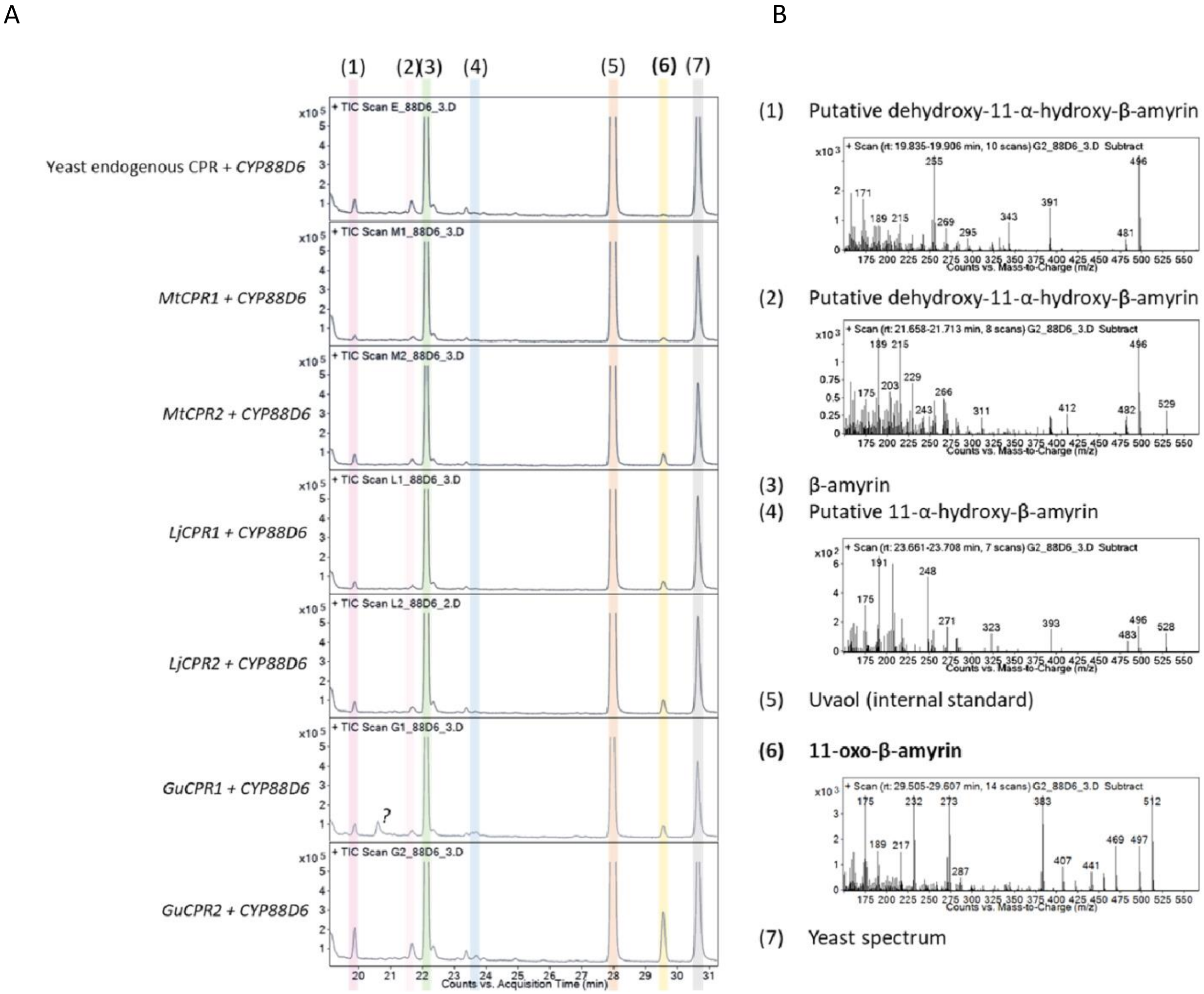

Figure S13. (A) GC-MS chromatogram of triterpenoids extracted from β-amyrin-producing *PSIII* yeast harboring *MtCPRs*, *LjCPRs*, and *GuCPRs* paired with *CYP88D6* using HP 5-MS column with optimized method with uvaol as internal standard. (B) Mass spectrum of each annotated peaks.

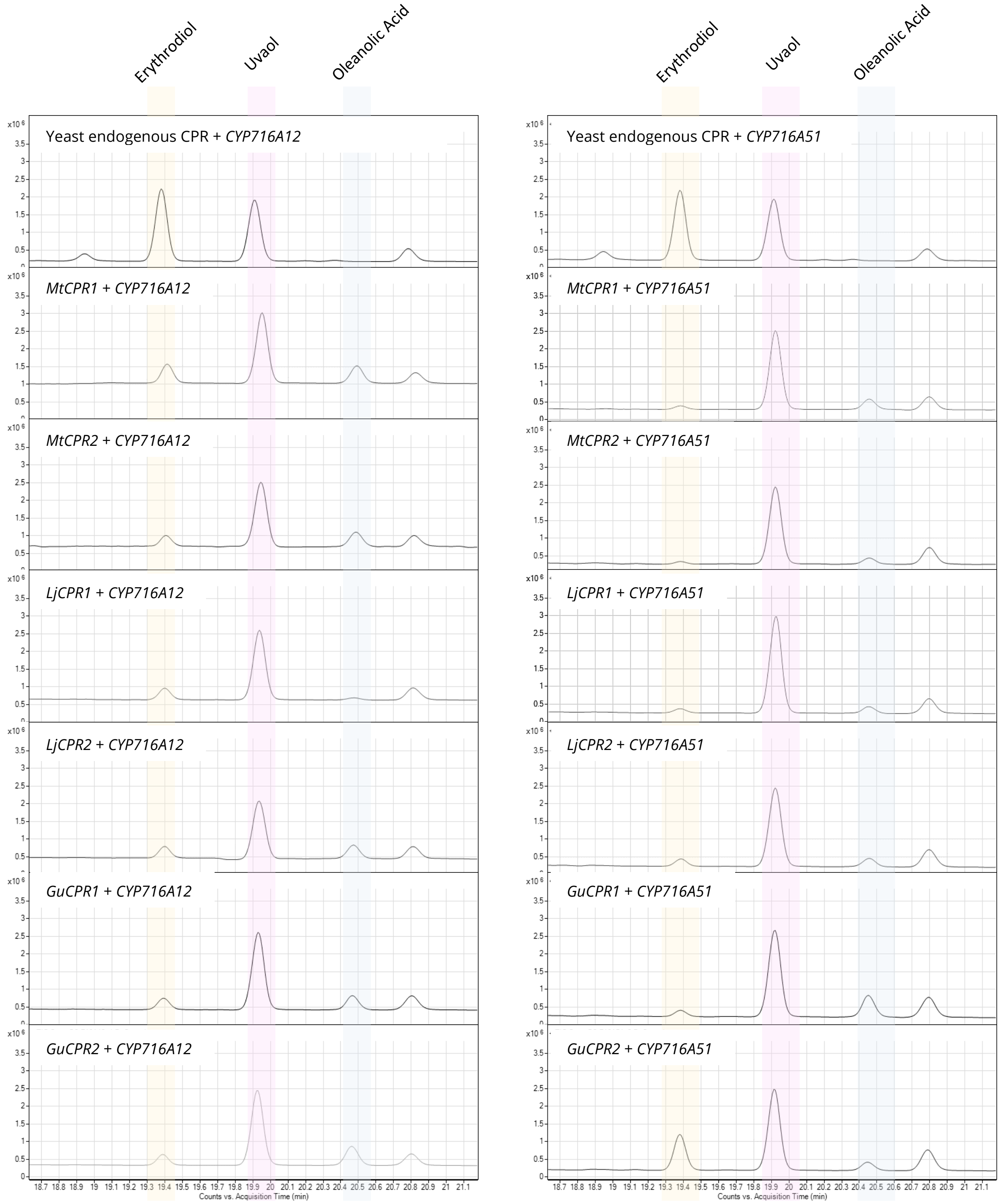

Figure S14. GC-MS chromatogram of triterpenoids extracted from INV*Sc1* yeast harboring *MtCPRs*, *LjCPRs*, and *GuCPRs* paired with *CYP71A612* and *CYP716A51* supplemented with erythrodiol as substrate using HP 5-MS column with uvaol as internal standard.
